# Slick Synthetic Approach to Various Fluoroalkyl Silsesquioxanes—Assessment of their Dielectric Properties

**DOI:** 10.3390/ma15248997

**Published:** 2022-12-16

**Authors:** Julia Duszczak, Katarzyna Mituła, Monika Rzonsowska, Paweł Ławniczak, Rafał Januszewski, Bartłomiej Szarłan, Beata Dudziec

**Affiliations:** 1 Faculty of Chemistry, Adam Mickiewicz University in Poznan, Uniwersytetu Poznanskiego 8, 61-614 Poznan, Poland; 2 Centre for Advanced Technologies, Adam Mickiewicz University in Poznan, Uniwersytetu Poznanskiego 10, 61-614 Poznan, Poland; 3 Institute of Molecular Physics Polish Academy of Sciences, Smoluchowskiego 17, 60-179 Poznan, Poland

**Keywords:** polyhedral oligomeric silsesquioxanes (SQs), double-decker silsesquioxane (DDSQ), hydrosilylation, platinum, fluorinated olefins, non-symmetric silanes, dielectric permittivity

## Abstract

We present a smart and efficient methodology for the synthesis of a variety of fluorinated silsesquioxanes (SQs) with diverse Si-O-Si core architecture. The protocol is based on an easy-to-handle and selective hydrosilylation reaction. An investigation on the placement of the reactive Si-HC=CH_2_ vs. Si-H in the silsesquioxane, as well as silane vs. olefin structure, respectively, on the progress and selectivity of the hydrosilylation process, was studied. Two alternative synthetic pathways for obtaining a variety of fluorine-functionalized silsesquioxanes were developed. As a result, a series of mono- and octa- T_8_ SQs, tri- ‘open-cage’ T_7_ SQs, in addition to di- and tetrafunctionalized double-decker silsesquioxane (DDSQ) derivatives, were obtained selectively with high yields. All products were characterized by spectroscopic (NMR, FTIR) techniques. Selected samples were subjected to the measurements revealing their dielectric permittivity in a wide range of temperatures (from −100 °C to 100 °C) and electric field frequencies (10^0^–10^6^ Hz).

## 1. Introduction

Novel insulating materials have become an urging field of research due to their importance in microelectronics [1]. Along with this development, new challenges for the size reduction of the individual components of these devices to correspond with the increase in the number of transistors have been appearing. This is correlated with some difficulties related to signal delay and the energy consumption that have become significant. The energy consumption is raised because the systems are functioning at higher frequencies. This dependence can be described by Equation (1):

P = αCfV^2^ (1)

where: P—energy consumption; α—conductor activity; C—capacitance of the output and input of transistors and the cable, f—current frequency and V—voltage of the power source. 

The requirements for the insulating materials also concern their appropriate mechanical properties (resistance to moisture, thermal stability) [2]. As a result of those needs, the use of silicon-based materials has gained a predominant significance [3]. It corresponds to the employment of not only simple poly-[4] or cyclosiloxanes [5] but organosilicons namely described as hybrid materials [6]. Within this group, a particularly important role possesses silsesquioxanes (POSS^®^, SQs). This is due to their physical properties and relatively simple and practically unlimited modification possibilities that affect their use as scaffolds for the modelling of new materials with a low dielectric constant (κ) under various conditions. This physical constant is also connected with a relative electric permittivity (ε) to describe their electrical properties, i.e., one of the crucial parameters when designing materials for the microelectronic industry [1]. Low- or ultra-low κ materials are of significant importance from the application point of view [7].

There are many materials tested in terms of their permittivity and they are often classified by their dielectric constant value as follows [8]:

- κ > 3.3–3.4—e.g., SiO_2_, fluorosilicate glass [1,8,9];
- 3.3 > κ > 2.7, e.g., organics: DLC (Diamond Like Carbon) and FDLC (Fluorinated Diamond-Like Carbon), SiCOH, parylenes, polynaphthalenes, silicon-substituted polyimides, polyquinoxalines, quinolines, norbornenes, benzocyclobutenes, indanes, perfluorocyclobutanes [8,10,11,12];
- 2.7 > κ > 2.2 and κ < 2.0—ultra-low κ materials, e.g., porous SiLK (Silicon Low κ), boron nitride nanotube (BNNT) material, porogen-free systems, aerogels, xerogels and f-xerogels, organosilicates) [8,13,14,15].

The above-mentioned silsesquioxanes are a group of organosilicon compounds of the general formula (RSiO_3/2_)_n_. The chemical formula does not reflect the multiplicity of their core architectures, varying from random, ladder, partial cage to cage, i.a. T_6_, T_8_ or T_10_ to even T_18_ discovered recently, and also so-called double-decker SQ (DDSQ) [16,17]. The variety of rigid core structures is also defined by the diversity of the amount of reactive/inert substituents anchored to the Si-O-Si core, from one to eight or more. For instance, the DDSQ compounds are known in the area of their double- and tetrafunctionalized derivatives [18,19,20]. These systems exhibit the organic–inorganic, i.e., hybrid nature of well-defined nano sizes (diameter of ca. 0.3–0.4 nm) and specific properties, i.a. oxidation resistance and non-toxicity, thermal stability, biocompatibility, etc. [16,21,22]. As a result, they may be applied in the chemistry of materials (as nanofillers or polymer modifiers), [23,24,25] in optoelectronics, [26,27] catalysis [28,29] or even medicine (e.g., drug delivery systems) [30,31,32]. The application potential of silsesquioxanes lies in the ease of their modifications and the adjustments of functional groups anchored to the inorganic Si-O-Si core. The crucial issue in this matter is the proper selection of the prefunctional moiety at SQs core for the selection of the respective reaction, usually catalytic, for its modification. Among the variety of catalytic processes, a few are of special importance due to their effectiveness and selectivity, i.e., hydrosilylation, cross-metathesis, coupling processes, e.g., Heck or Sonogashira, dehydrogenative coupling of Friedel-Crafts reaction [26,27,33,34,35,36,37].

Among the application studies is the introduction of polyhedral oligomeric silsesquioxane into polymers as their components and modifiers have been considered of significant importance. These materials have already shown potential applications, including low- κ insulators with dielectric constant values in a range of ca. 1.5–3 [38,39,40]. They may be obtained in several ways, e.g., hydrolytic condensation, hydrosilylation, thermal polymerization or curing and casting are some of these methods combinations [38,39,40,41,42,43,44,45]. Moreover, there are some reports on the dependence of dielectric constant value on the type and number of functional/inert groups in T_8_ and T_7_ open-cage SQs, e.g., alkyl, alkenyl, cycloalkyl, methacryl, ester or epoxide moieties, varying in the range of 2.4–3.0. The methods for their film formation include, e.g., chemical vapor deposition (CVD) also enhanced by plasma, spin-on deposition (SOD) or electrospun [46,47]. Interestingly, the SQs-based materials, in most cases, exhibited lower dielectric constants than the host polymers, and what is more, the dielectric constants can be tuned by adjusting the content of SQs. In general, there is a tendency for the dielectric constant value to decrease with the increasing content of SQs [42,48,49,50,51]. The fluorinated silsesquioxanes, mainly based on octasubstituted-T_8,_ but also DDSQs systems, are groups of compounds being building blocks of low-κ hybrid materials [42,44]. Within this group, modified polyimides, also with compounds containing fluorine, may be distinguished as being utilized for dielectric layers formation and hence appliance in the microelectronic industry [41,52,53,54,55,56,57,58].

However, the reports on the values of dielectric constant measured only for bare silsesquioxanes without any modification in their deposition are strongly limited. Therefore, these studies concern the elaboration of a smart methodology for the synthesis of a series of silsesquioxanes with a diverse amount of organofluorine substituents as well as the SQ’s core structure. Additionally, part of this research was the determination of the dielectric permittivity for selected samples measured in a wide range of temperature and field frequencies.

## 2. Materials and Methods

The experimental procedures, analytical data and NMR spectra of all isolated products are available in the ESI file.

### 2.1. Materials

The chemicals were purchased from the following sources: Sigma-Aldrich (St. Louis, MO, USA) for toluene, chloroform-*d*, chloroform, Karstedt’s catalyst—2% xylene solution [Pt_2_(dvs)_3_)] and silica gel 60. The following silsesquioxanes, **monoT_8_SiH, monoT_8_SiVi, triT_7_SiH, triT_7_SiVi, DDSQ-2SiH, DDSQ-2SiVi, DDSQ-2OSiVi, DDSQ-4OSiH, DDSQ-4OSiVi, octaT_8_SiH and octaT_8_SiVi,** were prepared according to the literature procedures [59,60,61] and so was the olefin CH_2_=CHCH_2_OCH_2_(CF_2_)_3_CHF_2_ (**F1**) and silane HSiMe_2_OSiMe_2_CH_2_CH_2_CH_2_OCH_2_(CF_2_)_3_CHF_2_ (**F5**) [62,63]. All solvents were dried over CaH_2_ before use and stored under argon over 4Å molecular sieves. All liquid substrates were also dried and degassed by bulb-to-bulb distillation. All syntheses were conducted under an argon atmosphere using standard Schlenk-line and vacuum techniques.

### 2.2. General Synthetic Procedures

#### 2.2.1. General Synthetic Procedure for Fluorinated Silsesquioxanes Obtained via Hydrosilylation of SQ-H with Olefins (F1, F2)

The procedure for the synthesis of **monoT_8_(F1)** is described as an example. To a two-neck round-bottom flask equipped with a condenser and magnetic stirrer, **monoT_8_SiH** (0.1009 g, 0.098 mmol) was placed in argon atmosphere along with anhydrous toluene (3.0 mL), 1.1 equiv. of olefin **F1** (0.0298 mL, 0.106 mmol) and 10^−4^ equiv. of [Pt_2_(dvs)_3_] (0.113 µL 9.8 × 10^−9^ mol). The reaction mixture was kept at 95 °C for 24 h. After cooling it to room temperature, the excess of olefin and solvent was evaporated under a vacuum and the crude product was purified using column chromatography (silica gel 60) using chloroform as an eluent. Evaporation of eluent gave an analytically pure sample of **monoT_8_(F1)** in 96% yield.

#### 2.2.2. General Synthetic Procedure for Fluorinated Silsesquioxanes Obtained via Hydrosilylation of SQ-Vi with Silanes (F3–F5)

The procedure for the synthesis of **monoT_8_(F5)** is described as an example. It is analogous to obtaining **monoT_8_(F1)**. The stoichiometry of respective reagents is as follows: [**monoT_8_SiVi**][**F5**][Pt_2_(dvs)_3_] = 1:1.2:10^−4^. **MonoT_8_(F5)** was obtained in 94% yield.

### 2.3. Measurements

#### 2.3.1. Nuclear Magnetic Resonance (NMR)

^1^H, ^13^C, and ^29^Si Nuclear Magnetic Resonance (NMR) were performed on Brucker Ultra Shield 600, 400 and 300 spectrometers, using CDCl_3_ as a solvent. Chemical shifts are reported in ppm with reference to the residual solvent (CHCl_3_) peaks for ^1^H and ^13^C and to TMS for ^29^Si NMR.

#### 2.3.2. FTIR Spectroscopy

Fourier-transform infrared (FTIR) spectra were recorded on a Nicolet iS5 (Thermo Scientific, Waltham, MA, USA) spectrophotometer equipped with a diamond ATR unit. In all cases, 16 scans at a resolution of 2 cm^−1^ were collected, to record the spectra in a range of 4000–650 cm^−1^.

#### 2.3.3. Elemental Analysis (EA)

Elemental analyses were performed using a Vario EL III instrument.

#### 2.3.4. Dielectric Relaxation Spectra (DRS)

Dielectric spectroscopy (DS) measurements were performed using an Alpha-A Novocontrol High-Performance Broadband Dielectric Spectrometer (Novocontrol GmbH, Montabaur, Germany) in a frequency range from 1 Hz to 10^6^ Hz and with voltage oscillations of ±1 volts. For dielectric measurements, the powder material was formed into the form of a pellet (*p* = 30 MPa, 293 K). The surfaces of the samples were covered with a silver paste (Hans Wolbring, Höhr-Grenzhausen, Germany).

To check the thermal stability of the powdered materials, the dielectric properties were studied using the following temperature procedure: in the beginning, the studied material was measured continuously at 20 °C for 5 h. Later, the sample was measured during cooling down to −100 °C with the continuous step of −0.45 K/min. After cooling, the sample was measured continuously during heating to 100 °C with a step of 0.45 K/min, and later, the measured material was cooled to 20 °C. After cooling, the sample was measured at a temperature of 20 °C for another hour. The temperature was controlled using a Quatro Cryosystem (Montabaur, Germany) with a stabilization temperature of ±0.05 K.

The dielectric measurements were also performed for the materials in the form of films of the selected compounds. The films were obtained by sputtering the solution of the material on the steel plates. Thin films in one layer were prepared by a spin-coating technique using a 5–10 wt% solution of compounds in DCM or DCM/THF (1:1) that was deposited on the steel plates (spin speed of 100 rpm for 40 s.) to form homogeneous surface. The film thickness was in the range of 500–1500 nm. The measurements were performed at 20 °C for 5 h, using the same equipment as in the case of the powder sample.

## 3. Results and Discussion

### 3.1. Synthetic Methodology to form Fluorinated Silsesquioxanes

**Scheme 1:**
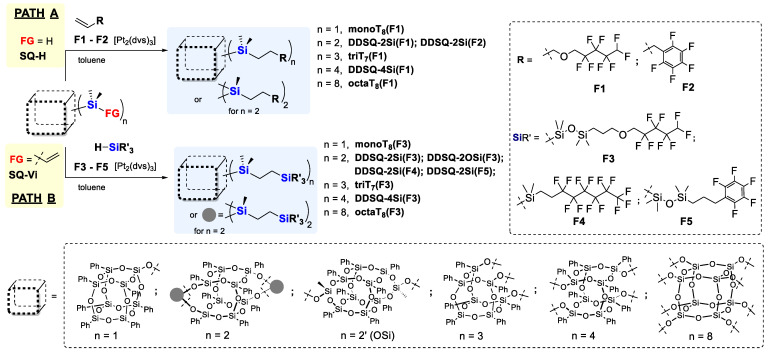
The reaction path for the synthesis of fluorinated silsesquioxanes via hydrosilylation of SQ-H with olefins (**F1**, **F2**) or SQ-Vi with silanes (**F3**–**F5**) leading to, respectively, substituted products varying in the SQ-core architecture and number of fluorinated substituents.

Silsesquioxanes are known to be used as components of materials exhibiting low permittivity (low-κ materials), especially using fluoro-organosilicon derivatives [8,46,47]. The synthetic strategy for these compounds may be not straight and is limited to the functional group attached to the SQ’s core. Therefore, these studies concern the elaboration of a smart methodology for the synthesis of a series of silsesquioxanes with a diverse amount of organofluorine substituents as well as the SQ’s core structure. The synthetic protocol is based on a hydrosilylation reaction performed in a two-way form (Scheme 1):

- PATH A—concerns the hydrosilylation of fluoroolefins with SQs possessing reactive Si-H (**SQ-H**) moiety varied from 1 to 8 (**F1**, **F2**);
- PATH B—is connected with the use of silsesquioxanes with Si-HC=CH_2_ group(s) (**SQ-Vi**) (from 1 to 8) to be hydrosilylated with silanes bearing fluorine atoms (**F3**–**F5**)

For the studies, the diverse type of SQs was applied, i.e., varying not only in the core type but also the amount of functional Si-H or Si-HC=CH_2_ groups from one (**monoT_8_**), two (**DDSQ-2Si** or **DDSQ-2OSi**), three (**triT_7_**), four (**DDSQ-4Si**) or eight (**octaT_8_**). Additionally, the reagents containing the fluorine atoms were also diverse, i.e., olefins: with aliphatic chain (**F1**) and aromatic group (**F2**), as well as respective silanes with analogous substituents (aliphatic: **F3**, **F5** and aromatics: **F4**). The synthetic procedure was based on Pt-Karstedt’s catalyst ([Pt_2_(dvds)_3_]) used in 10^−4^ mol of Pt loading per 1 mol of Si-H group and performed at 95 °C for 24 h. This enabled the >99% consumption of SQs applied in the studies, i.e., **SQ-H** and **SQ-Vi**, and its result was monitored using the FTIR and ^1^H NMR spectroscopy. The absence of band at FTIR spectra characteristics for stretching vibrations of Si-H bonds (at ῡ = 895 cm^−1^) and the respective doublet of doublets at ^1^H NMR spectra (δ in a range of ca. 5.5–6.5 ppm for CDCl_3_, depending on the SQs core type and amount of vinyl moieties). The reagents’ stoichiometry was maintained at equivalent. We noted previously that alkene excess may affect the reaction time, but mainly in its initial time and does not significantly reduce the total reaction time [64]. As it is known, hydrosilylation may occur with side reactions, e.g., hydrogenation of olefins, and dehydrogenative silylation. It also might not be selective and a mixture of *α*- and *β*-hydrosilylation products may be formed. It should be noted that the studied hydrosilylation reaction of **SQ-H** with 5-allyl-1,1,2,2,3,3,4,4-octafluoropentyl ether (**F1**) and 1-allyl-2,3,4,5,6-pentafluorobenzene (**F2**) were regioselective towards the formation of *β*-hydrosilylation products. The selectivity of the reaction was monitored via ^1^H NMR of the product and verification of CH_2_ moiety presence—characteristics of *β*-hydrosilylation compound and absence of CH and CH_3_ groups—the features of *α*-hydrosilylation products (see Supporting Information). This observation was verified for PATH A, i.e., the hydrosilylation of fluorinated olefins (**F1**, **F2**) by respective silsesquioxanes **SQ-H** (**monoT_8_H**, **DDSQ-2SiH**, **triT_7_H**, **DDSQ-4SiH**, **octaT_8_H**, Scheme 1).

**Figure 1:**
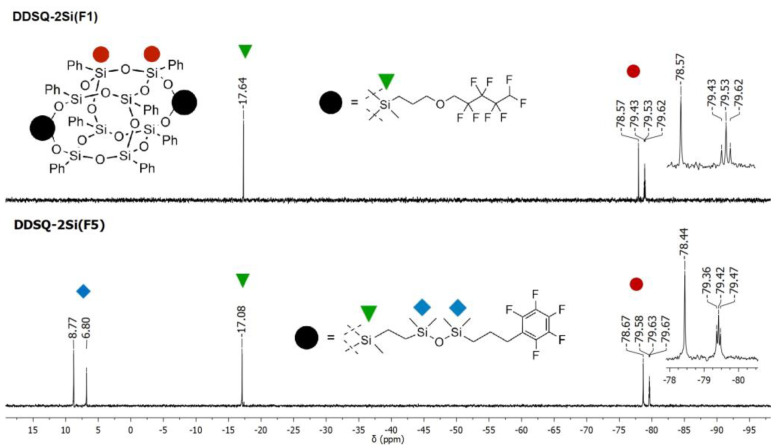
Selected range of stacked ^29^Si NMR spectra of **DDSQ-2Si(F1)** and **DDSQ-2Si(F5)**.

It is worth noting that the changes in the chemical surrounding of the functional Si atoms affect their resonance lines placement in ^29^Si NMR spectra. Interestingly, the amount of the fluorine atoms located in the fragment anchored to the SQs core insignificantly influences the shift of the Si peak, which is shown for **DDSQ-2Si(F1)** and **DDSQ-2Si(F5)** ^29^Si NMR spectra (Figure 1). The electronic impact of those moieties, regardless of the aliphatic of aromatic units present or even though possessing highly electronegative fluorine atoms, is rather limited. The functional Si^D^ atoms connected with the respective fluorosubstituted groups are placed at ca. −17.64 and −17.08 ppm, respectively. Additional resonance lines at 6.80 and 8.77 ppm correspond with the Si^M^ atoms in the additional siloxy moiety in the fluoroalkyl chain of **F5**.

**Figure 2:**
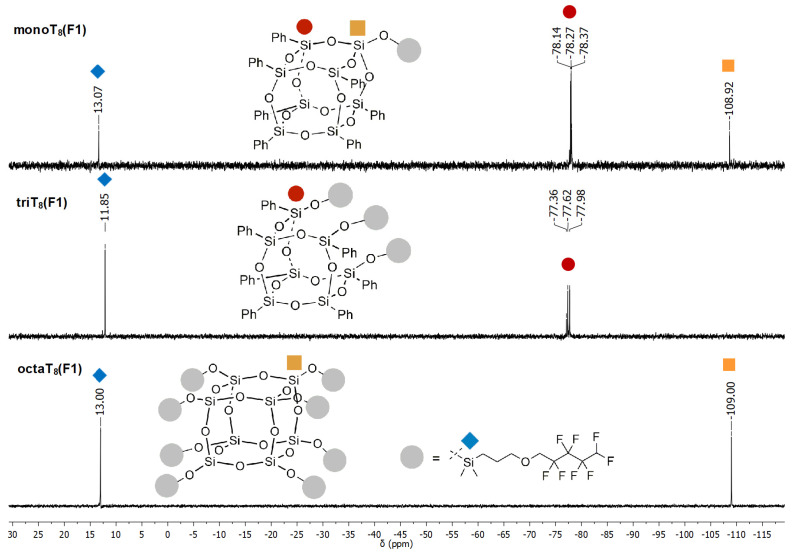
Selected range of stacked ^29^Si NMR spectra of **monoT_8_(F1)**, **triT_8_(F1)** and **octaT_8_(F1)**.

**Figure 3:**
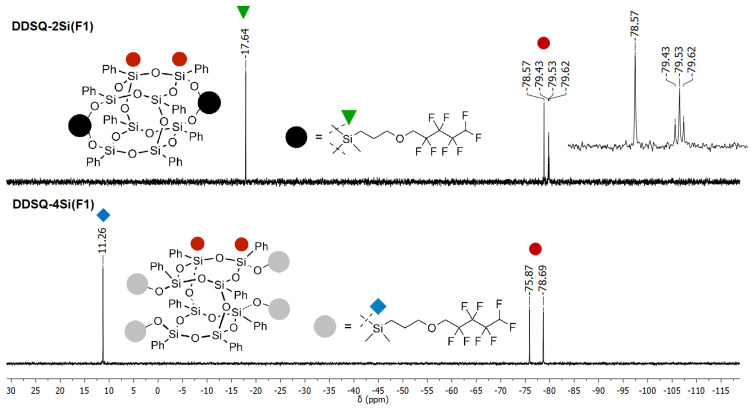
Selected range of stacked ^29^Si NMR spectra of **DDSQ-2Si(F1)** and **DDSQ-4Si(F1)**.

It should be emphasized that a more important aspect should be taken into consideration, i.e., the order of the silicon atoms. It may visualize how the placement of the Si^M^, Si^D^, Si^T^ and Si^Q^ atoms in ^29^Si NMR changes in the obtained products varying in the SQs core type (Figure 2 and Figure 3). In the case of **monoT_8_(F1)**, **triT_7_(F1)** and **octaT_8_(F1)** beside the typical placement of Si^T^ and Si^Q^ peaks, i.e., in the range of −77.36 to −78.37 ppm for **monoT_8_(F1)**, **triT_7_(F1)** and −109 vs. −108.92 ppm, respectively, **octaT_8_(F1)** and **monoT_8_(F1)**, the functional Si^M^ was located at ca. from 11.85 to 13.07 ppm (Figure 2).

More visible changes could be noticed in the case of DDSQs products, i.e., **DDSQ-2Si(F1)** and **DDSQ-4Si(F1)**, where the Si^T^ DDSQ-derived signals were present in a typical range of −75.87 to −79.62 ppm. Interestingly, and regardless of fluorosubtituent, the Si^D^ resonance lines were up-field shifted to −17.64 ppm for **DDSQ-2Si(F1)** when compared to Si^M^ down-field shifted to 11.26 ppm (Figure 3). 

Additionally, the methodology for the synthesis of the fluorinated silsesquioxanes was also verified in terms of the reverse Si-H and Si-CH=CH_2_ reactive groups placement, PATH B, i.e., hydrosilylation of SQ-Vi (**monoT_8_SiVi**, **triT_7_SiVi**, **DDSQ-2SiVi**, **DDSQ-2OSiVi**, **DDSQ-4OSiVi**, **octaT_8_SiVi**) by three types of silanes containing respective fluorosubstituents to olefins used before (**F3**–**F5**) (Scheme 1). The reaction conditions were also analogous to the previous ones. In this case, the process was also regioselective towards the formation of only *β*-hydrosilylation products. 

**Figure 4:**
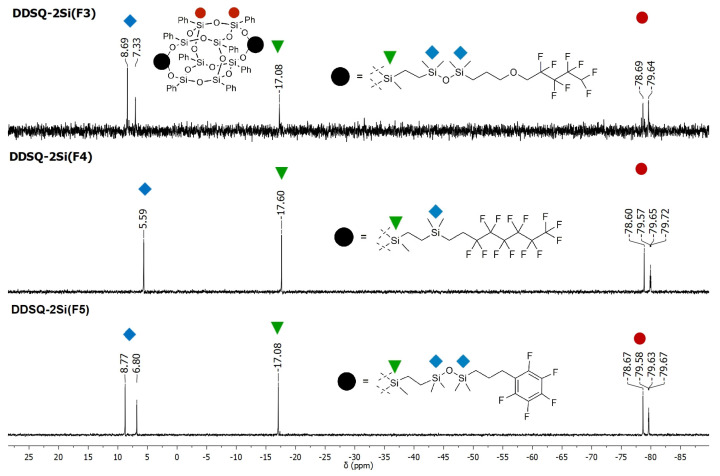
Selected range of stacked ^29^Si-NMR spectra of **DDSQ-2Si(F3)**, **DDSQ-2Si(F4)** and **DDSQ-2Si(F5)**.

Interestingly, in the case of **DDSQ-2SiVi** to react with **F4** and **F5** silanes, the mixture of *cis-* and *trans-*geometric isomers was isolated (e.g., DDSQ core resonance lines for **DDSQ-2Si(F4)**: δ = −78.60, −79.57 (cis), −79.64 (trans), −79.72 (cis) and **DDSQ-2Si(F5)**: δ = −78.67, −79.58 (cis), −79.63 (trans), −79.67 (cis)), which was observed in ^29^Si NMR analysis, respectively. When silane **F3**, i.e., with an additional Si-O-Si bridge between the Si-H reactive group and the fluorosubstituted moiety was applied, the *trans-*isomer of **DDSQ-2Si(F3)** was isolated and proved by the presence of respective DDSQ core resonance lines at ^29^Si NMR: δ = −78.69, −79.64 (trans) (Figure 4). It should be mentioned that, for this product, both *cis*- and *trans-*isomers are postulated to be formed. However, due to the different physicochemical properties of these isomeric structures, their separation may be performed [65,66]. Herein, the trans-isomer of **DDSQ-2Si(F3)** was isolated exclusively during purification on column chromatography, as the amount of cis-isomer was residual. 

The observations concerning the appearance of the ^29^Si NMR spectra and placement of peaks derived from the respective type of Si atoms in the obtained products are analogous to the compounds obtained via PATH A. In this way, the resonance lines corresponding with the Si^D^ atoms are up-field-shifted (**DDSQ-2Si(F3)**) when compared with the down-field placement of Si^M^ atoms in **DDSQ-2OSi(F3)**, **DDSQ-4Si(F3)** and **monoT_8_(F3)**, **triT_8_(F3)** and **octaT_8_(F3)**, respectively (Figures S44 and S45 in Supplementary Information). 

**Figure 5:**
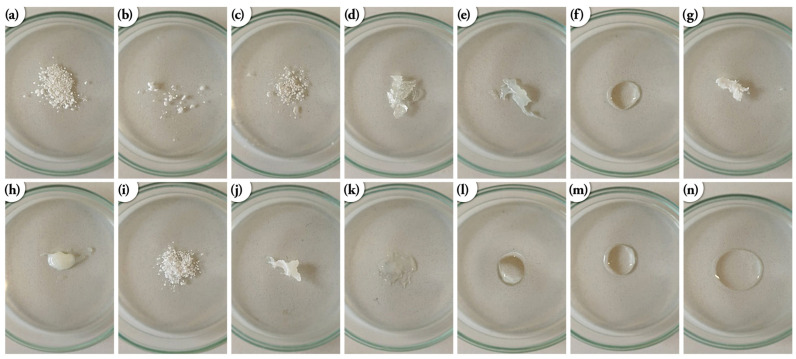
Images of physical state of the obtained compounds (**a**) **monoT_8_(F1)**, (**b**) **DDSQ-2Si(F1)**, (**c**) **DDSQ-2Si(F2)**, (**d**) **triT_7_(F1)**, (**e**) **DDSQ-4Si(F1)**, (**f**) **octaT_8_(F1)**, (**g**) **monoT_8_(F3)**, (**h**) **DDSQ-2Si(F5)**, (**i**) **DDSQ-2Si(F3)**, (**j**) **DDSQ-2Si(F4)**, (**k**) **DDSQ-2OSi(F3)**, (**l**) **triT_7_(F3)**, (**m**) **DDSQ-4Si(F3)**, (**n**) **octaT_8_(F3)**.

All products based on silsesquioxanes are air-stable solids or oils or waxy, white solids, which are depicted in Figure 5. They were obtained in very high yields in a range of 87–96%. They are soluble in organic solvents like DCM, CDCl_3_, THF and toluene but not in, e.g., methanol, or MeCN. They were isolated and characterized using spectroscopic methods (NMR, FTIR) and elemental analysis.

### 3.2. Dielectric Properties of Fluorinated Silsesquioxanes

**Figure 6:**
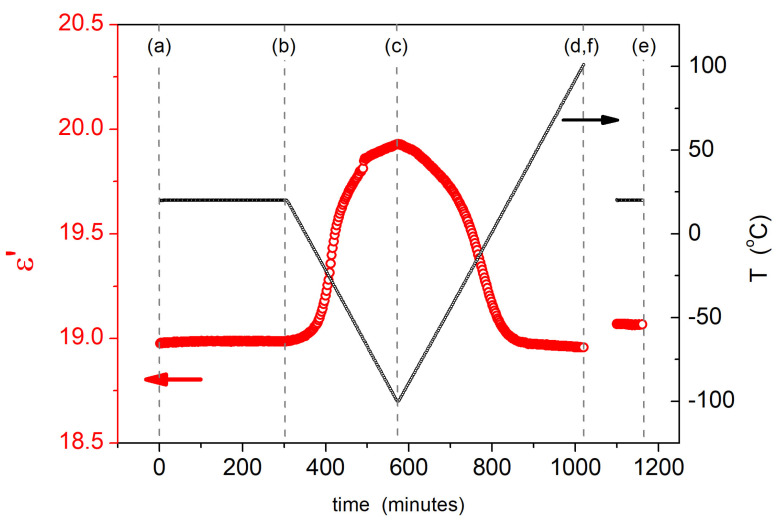
Typical time and temperature dependences of the real part of complex permittivity ε’ of **DDSQ-2SiVi** compound, measured at 100 Hz. Letters denote characteristics temperatures: (**a**) 20 °C (pristine sample, before thermal studies); (**b**) 20 °C (after 300 min, at T = 20 °C), (**c**) the minimum temperature at −100 °C, (**d**,**f**) maximum temperature at 100 °C and (**e**) 20 °C (after thermal treatment).

Fluorinated silsesquioxanes have been promising candidates for their use as interlayer dielectric or intermetal dielectric materials with great application potential in the microelectronic industry [47,58,67]. The dielectric properties of silsesquioxanes and their derivatives were measured in a frequency range of 1 Hz to 1 MHz and in a wide temperature range (from −100 °C to 100 °C). Figure 6 shows an example of the time and temperature dependence of the real part of permittivity of the **DDSQ-2SiVi** sample, measured at a fixed frequency of 100 Hz (more data are presented in the Supplementary Information). In the first cycle, measurements were carried out at RT temperature for 300 min and the value of dielectric permittivity was stable and had a value of ~18.95. During the next cycle, the sample was cooled to T = −100 °C and one could observe an increase of dielectric permittivity, which reached a maximum value of ~19.92 at T = −100 °C. The following third cycle presented heating of the measured material from −100 °C to 100 °C, and the permittivity decreased to the same value as observed at the beginning of the experiment. However, after coming back to the RT temperature (last temperature cycle), dielectric permittivity slightly increased to a value of ~19.07 (less than 1% change compared to the pristine sample).

**Figure 7:**
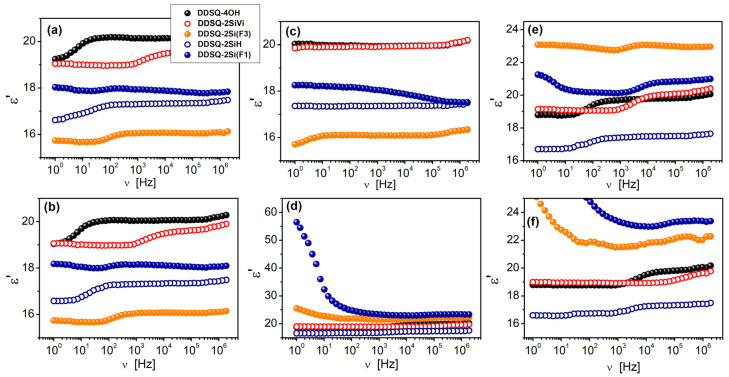
Frequency dependence of dielectric permittivity measured for respective compounds: **DDSQ-4OH**, **DDSQ-2SiVi**, **DDSQ2Si(F3)**, **DDSQ-2SiH**, **DDSQ-2Si(F1)** at different temperatures: (**a**) 20 °C (pristine sample, before thermal studies), (**b**) 20 °C (after 300 min, at T = 20 °C), (**c**) −100 °C (minimum temperature), (**d**) 100 °C (maximum temperature) and (**e**) 20 °C (after thermal treatment). Plot (**f**) shows parts of the data collected at T = 100 °C.

Figure 7 presents a comparison of the dielectric properties of the powdered samples of fluorinated silsesquioxanes for selected temperatures. Dielectric permittivity measured for the pristine initial material (Figure 7a) is the highest from the compared derivatives, with a broad plateau in the 20 < ν < 20 × 10^5^ Hz range and value of ~20.18 and reaches a maximum value at the highest frequency of 20.38 (at ν = 2 × 10^6^ Hz). The lowest value of ε’ for pristine material (before thermal treatment) is observed for the **DDSQ2Si(F3)** (16.1 at maximum and 15.68 at minimum). After 300 min of measuring at T = 20 °C (Figure 7b), the frequency dependences are the same, indicating the very good stability of the materials. Data, collected at T = −100 °C, show that changes caused by the cooling process are almost negligible (Figure 7c). The opposite effect is observed at the highest measured temperature, T = 100 °C (Figure 7d,e), as in the case of samples **DDSQ-2Si(F1)** and **DDSQ2Si(F3)** dielectric permittivity increases, and reaches the maximum value of 25.8 and 65.6 at 1Hz, respectively. After cooling back to 20 °C (Figure 7e), the dielectric permittivity of **DDSQ-2Si(F1)** and **DDSQ2Si(F3)** samples decreases. However, the measured values of ε’ are higher, approximately ~12% and 45% higher than for the pristine material. In contrast, the characteristics of the initial material (DDSQ) are almost the same at all stages of thermal studies.

The values of dielectric permittivity, which depended on the frequency for measurements performed at 20 °C, are collected in Table 1. The values of dielectric permittivity at −100 °C and 85 °C, respectively, are presented in Tables S2 and S3 in the Supplementary Information.

**Figure 8:**
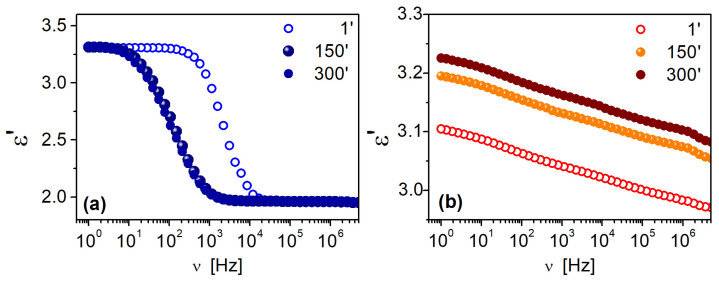
Frequency dependence of dielectric permittivity, measured for **DDSQ-2Si(F1)-f** sample (**a**) and **DDSQ-2Si(F3)-f** sample (**b**) in form of thin films. Presented data were collected after 1 min, 150 min and 300 min at T = 20 °C.

In addition to the studies of the powdered materials, measurements were also performed of the thin films of the selected **DDSQ-2Si(F1)-f** and **DDSQ2Si(F3)-f** materials for 300 min at temperature T = 20 °C. Results obtained at the beginning (1 min), at the middle (150 min) and at the end of the studies (300 min) are presented in Figure 8.

**Figure 9:**
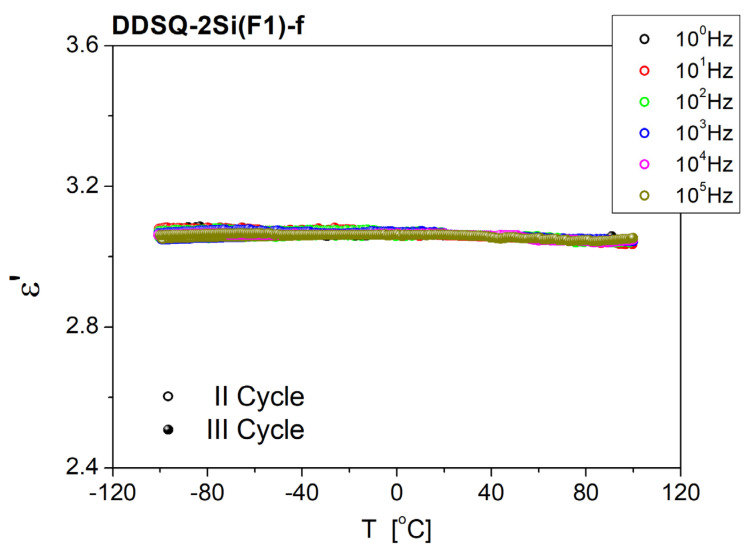
Temperature dependence of dielectric permittivity, measured for a thin film of **DDSQ-2Si(F1)-f**. II cycle—sample was cooled from RT to T = −100 °C; III cycle—sample heated from −100 °C to 100 °C.

**Figure 10:**
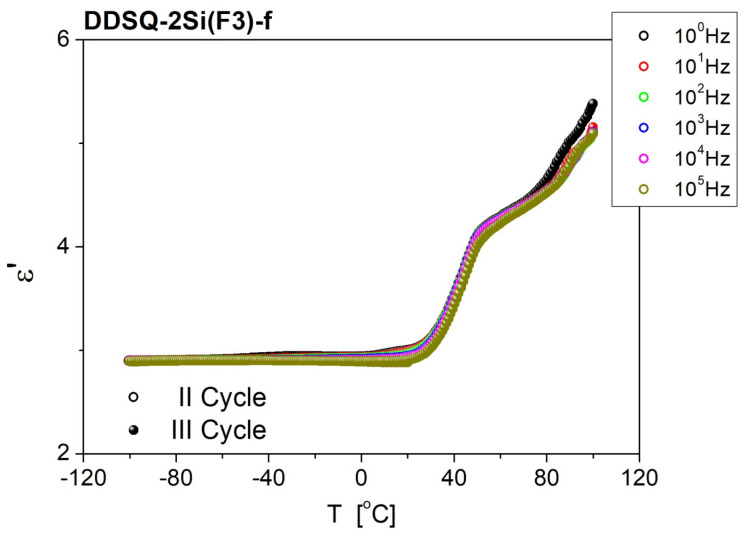
Temperature dependence of dielectric permittivity, measured for a thin film of **DDSQ-2Si(F3)-f.** II cycle—sample was cooled from RT to T = −100 °C; III cycle—sample heated from −100 °C to 100 °C.

Materials in the form of thin films show remarkably smaller values of ε’. The stability of the materials is satisfying. The frequency dependence of the **DDSQ-2Si(F1)-f** sample shows dispersion, as permittivity decreases with increasing frequency, to ~1.96 at ν = 2 × 10^4^ Hz and above at the first measured point (see Figure 8a). With time, the transition region from the high to low values of ε’ moves to lower frequencies (to 3 × 10^3^ Hz). In the case of sample **DDSQ-2Si(F3)-f** (Figure 8b), permittivity is frequency-dependent in all measuring frequency windows, although the difference between maximum and minimum is small, ~3.11 at ν = 1 Hz and ~2.97 at ν = 5 × 10^6^ Hz, respectively. With time, permittivity increases but the shape of the characteristics are the same. After 300 min, ε’ reaches the maximum value of 3.23 at ν = 1 Hz and the minimum of 3.09 at ν = 5 × 10^6^ Hz. The temperature dependence of dielectric permittivity, respective samples: **DDSQ-4OH**, **DDSQ-2SiVi**, **DDSQ-2SiH**, **DDSQ2Si(F3)**, **DDSQ-2Si(F1)**, are presented as charts in Figures S46–S50 in the Supplementary Information. What is worth noting, the values of dielectric permittivity of **DDSQ-4OH**, **DDSQ-2SiVi** and **DDSQ-2SiH** is decreasing with the temperature raise to maintain the plateau (Figures S46–S48). In contrast, the solid samples **DDSQ2Si(F3)** and **DDSQ-2Si(F1)** exhibited a reverse trend and a significant increase in dielectric permittivity, which is especially visible above 50 °C (Figures S49–S50). However, the temperature dependence of dielectric permittivity measured for the film samples **DDSQ-2Si(F1)-f** and **DDSQ-2Si(F3)-f** exhibit noteworthy behavior. For **DDSQ-2Si(F1)-f**, the value of ε’ is constant (ca. ε’ = 3.1) with the temperature raised from −100 °C to +100 °C in a whole range of field frequencies. The **DDSQ-2Si(F3)-f** displays a constant value of ε’ (ca. ε’ = 2.9) from −100 °C to ca. 30 °C, and its substantial increase may be noted further on with the temperature raise to +100 °C. It is graphically depicted in Figure 9 and Figure 10.

Commonly, dielectric permittivity decreases with increasing frequency, as in the case of **DDSQ-2Si(F1)** pellet and **DDSQ-2Si(F1)-f** and **DDSQ2Si(F3)-f** films. The observed effect in the **DDSQ-2Si(F3)** material is probably caused by some parasite effect, although before each measurement, the calibration was performed according to the proposed Novocontrol apparatus. One of the possible explanations for this effect can be the so-called “fringe effect”. The measured sample is placed between the plates of the sample holder and forms a capacitor. A voltage applied between these plates results in a dielectric field between them. Of course, this electric field exists not just directly between the plates but also extends some distance away. This extending field is known as a fringing field. This additional outside field may cause changes in capacitance (i.e., increasing the permittivity). This effect is not observed in the film, as the ratio of sample diameter and thickness is high. It is worth noticing, that the difference between the plateau region in the lower-frequency part and the high-frequency part where the permittivity increases are less than 3.5%, and it does not change the main observations and conclusions of the dielectric measurements. However, the issue of dielectric permittivity value dependence on the sample morphology or the method of its preparation seems to be very interesting [42,58,68]. It will be a subject of our further research.

## 4. Conclusions

Herein, we showed a hydrosilylation-based synthetic protocol for the formation of a variety of fluorinated silsesquioxanes (SQs). The elaborated conditions enable the regioselective formation of *β*-hydrosilylation products regardless of the placement of reactive Si-H vs. Si-HC=CH_2_ moiety at the SQ’s core. Diverse SQ core architectures, i.e., mono- and octa- T_8_, tri- ‘open-cage’ T_7_ SQs and di- and tetra- DDSQs, possessing varied amounts of reactive groups, were tested. As a consequence, a group of, respectively, fluorosubstituted silsesquioxane derivatives were obtained selectively and with high yields that were thoroughly examined using spectroscopic (NMR, FTIR) techniques. Interesting observations concerning the ^29^Si NMR analysis of the obtained products were made in terms of the tendency of resonance line shifts affected more by the type of the functional silicon atom (Si^M^_,_ Si^D^_,_ Si^T^) than the presence of fluorine atoms. Additionally, the samples susceptible to the form of pellets or thin films deposited on steel plates (obtained by spin-coating technique) were subjected to dielectric permittivity measurements. The analysis was also performed for respective substrates, i.e., **DDSQ-4OH**, **DDSQ-2SiVi** and **DDSQ-2SiH**. It may be noticed that fluorosubstituted groups insensibly decrease the value of dielectric permittivity for solid samples that are not changing highly in applied field frequencies from 10^0^ to 10^6^ Hz. Interestingly, for the samples analyzed as films, a significant decrease in the values of ε’ may be noted for the values of ca. ε’ = 3. It is visible, especially for the **DDSQ-2Si(F1)-f** sample, that this behavior is constant regardless of temperature and field frequency changes. This observation may be valuable for materials chemists in terms of the potential applications of the presented compounds in the modification of polymeric matrixes to be applied to microelectronics.

## Figures and Tables

**Table 1:** Values of dielectric permittivity, measured at 20 °C at 10^0^ × 10^6^ Hz.

Sample	ε at 20 °C						
	1 Hz	10 Hz	100 Hz	1 kHz	10 kHz	100 kHz	1 MHz
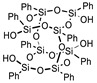 **DDSQ-4OH**	19.45	20.02	20.22	20.22	20.17	20.19	20.09
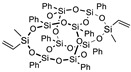 **DDSQ-2SiVi**	19.05	19.00	18.98	19.13	19.49	19.60	19.79
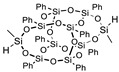 **DDSQ-2SiH**	16.62	16.91	17.27	17.31	17.33	17.35	17.43
**DDSQ-2Si(F1)**	18.04	17.88	17.97	17.94	17.88	17.81	17.81
**DDSQ-2Si(F3)**	15.74	15.68	15.89	16.06	16.07	16.05	16.09
**DDSQ-2Si(F1)-f** *	3.06	3.06	3.05	2.72	1.87	1.80	1.80
**DDSQ-2Si(F3)-f** *	2.88	2.86	2.84	2.82	2.80	2.78	2.76

For dielectric measurements, the powder material was formed into the form of a pellet. * Thin films in one layer prepared by a spin-coating technique using 5–10 wt% solutions of co-polymers in DCM (or DCM/THF (1:1)) deposited on the steel plate.

## Data Availability

Data are contained within the article and Supplementary Information file.

## Supplementary Materials

The following supporting information can be downloaded at: https://www.mdpi.com/article/10.3390/ma15248997/s1, list of compounds (Table S1), spectroscopic (NMR and FT-IR) spectra of obtained compounds (Figures S1–S45) as well as charts of dielectric permittivity for compounds (Figures S46–S50, Tables S2 and S3). Ref. [69] was cited in the Supplementary Information.
