# Supplementary material for: Slick Synthetic Approach to Various Fluoroalkyl Silsesquioxanes—Assessment of their Dielectric Properties
Source: Materials (Basel). 2022 Dec 16;15(24):8997. doi: 10.3390/ma15248997 (PMC9785758; doi:10.3390/ma15248997)
Supplement: Supplementary file 1 [file materials-15-08997-s001.zip › materials-2076329-supplementary materials.pdf]

Supporting Information for

Slick Synthetic Approach to Various Fluoroalkyl Silsesquioxanes - - Assessment of their Dielectric Properties

Julia Duszczak[1,2], Katarzyna Mituła[1,2], Monika Rzonsowska[1,2], Paweł Ławniczak[3], Rafał Januszewski[1,2], Bartłomiej Szarłan[1,2], Beata Dudziec[1,2,]*

[1] Faculty of Chemistry, Adam Mickiewicz University in Poznan, Uniwersytetu Poznańskiego 8, 61-614 Poznan, Poland;
[2] Centre for Advanced Technologies, Adam Mickiewicz University in Poznan, Uniwersytetu Poznańskiego 10, 61-614 Poznań, Poland
[3] Institute of Molecular Physics Polish Academy of Sciences, Smoluchowskiego 17, 60-179 Poznan, Poland.
* Correspondence: *beata.dudziec@gmail.com*

Table of contents:

1. Table of isolated compounds……………………………………………………... ••• S -2-
2. Characterization data of obtained products (^{1}H, ^{13}C, ^{29}Si NMR spectra and FT-IR data)……………………………………………………………….... ••• S -4-
3. Additional ^{29}Si NMR spectra comparisons…………………………………….. ••• S -33-
4. Dielectric Relaxation Spectra and Tables……………………………………….... ••• S -34-

1. Table S1. The list of isolated fluorinated silsesquioxanes

Structure	Compound Abbrev.	NMR spectra page:
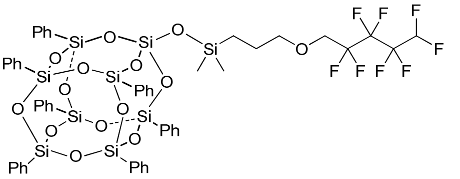	**monoT$_8$(F1)**	4-5
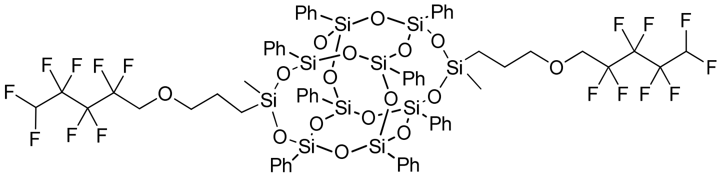	**DDSQ-2Si(F1)**	6-7
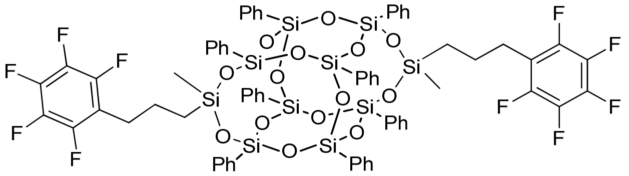	**DDSQ-2Si(F2)**	8-9
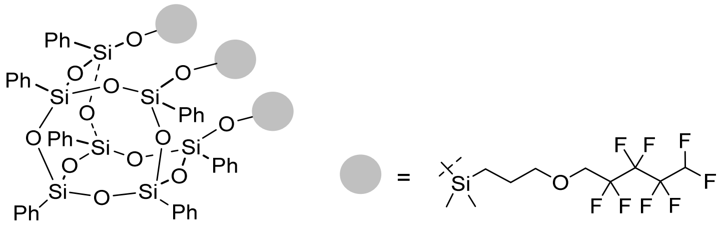	**triT$_7$(F1)**	10-11
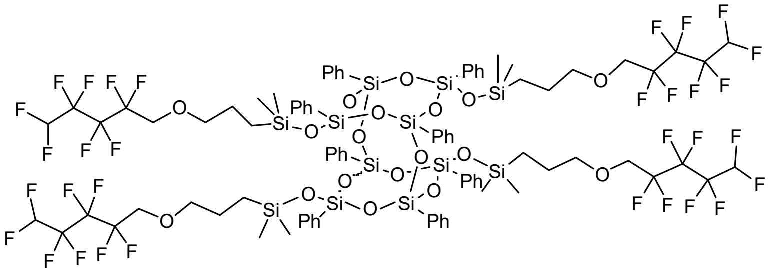	**DDSQ-4Si(F1)**	12-13
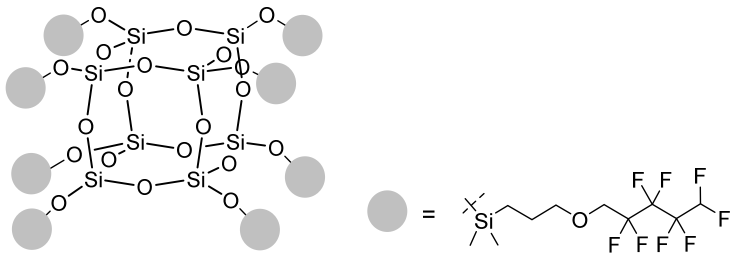	**octaT$_8$(F1)**	14-15

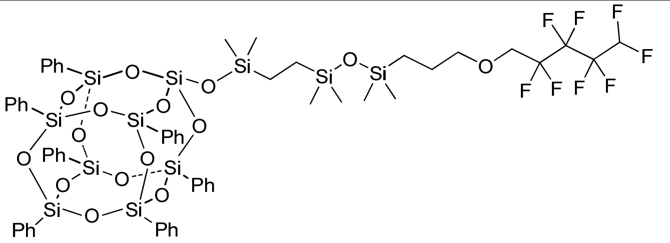	**monoT$_8$(F3)**	16-17
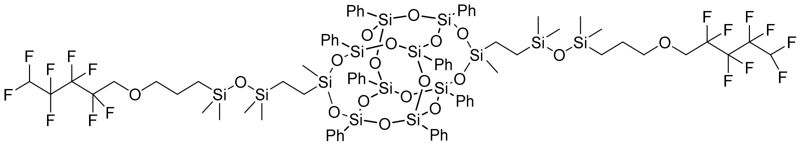	**DDSQ-2Si(F3)**	18-19
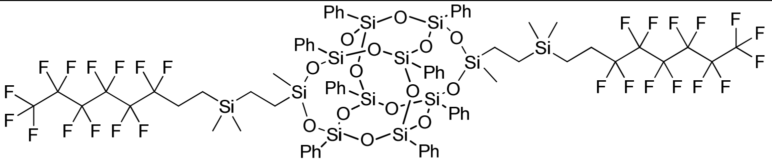	**DDSQ-2Si(F4)**	20-21
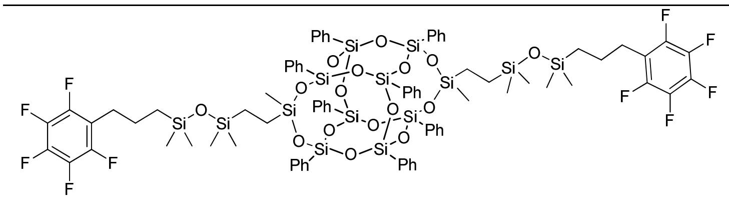	**DDSQ-2Si(F5)**	22-23
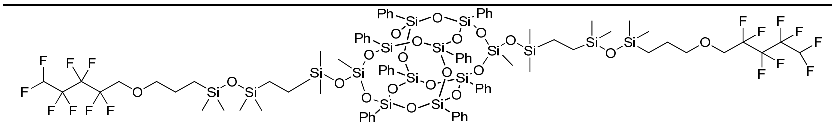	**DDSQ-2OSi(F3)**	24-25
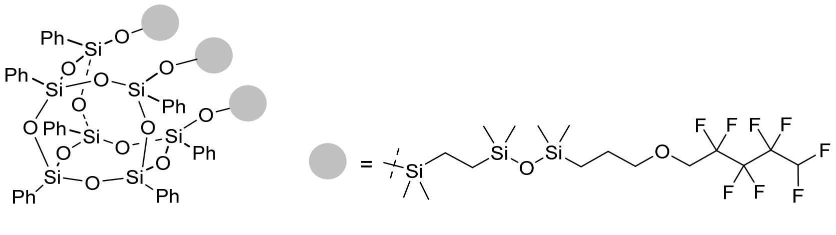	**triT$_7$(F3)**	26-27
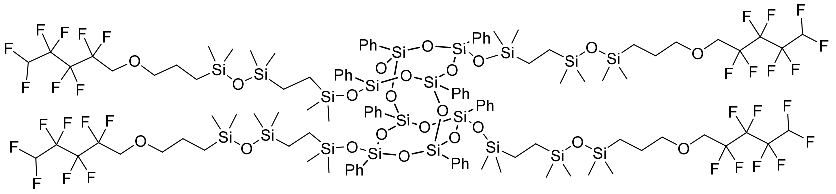	**DDSQ-4Si(F3)**	28-30
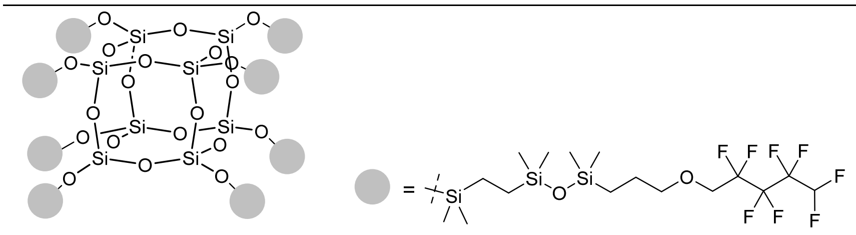	**octaT$_8$(F3)**	31-32

2. Characterization data of obtained products (^{1}H, ^{13}C, ^{29}Si NMR spectra and FT-IR data)

monoT$_8$(F1)

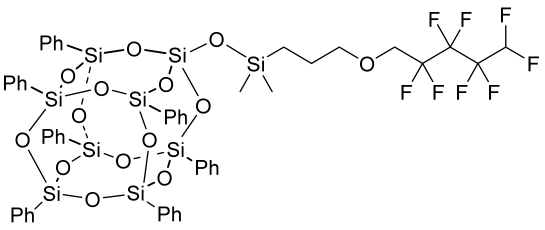

White solid. Isolated Yield 94%

^{1}H NMR (300 MHz, $CDCl_3$, ppm): δ = 0.09 (s, 6H, $Si(CH_3)_2$), 0.49-0.55 (m, 2H, Si-CH_2-), 1.52-1.57 (m, 2H, -CH_2-), 3.31 (t, J_{H-H}=6.7 Hz, 2H, -CH_2-O), 3.74 (t, J_{H-H}= 14.0Hz, 2H, -O-CH_2-), 6.00 (tt, , J_{H-H} = 52.1, 5.6 Hz, 1H, -CH-F_2), 7.32-7.48, 7.71-7.78 (m, 35H, Ph);

^{13}C NMR (101 MHz, $CDCl_3$, ppm): δ = -0.25 ($SiCH_3$), 13.53 (Si-CH_2-), 23.10 (-CH_2-), 67.24-67.74 (-CH_2-O-), 75.57 (O-CH_2-), 105.23 (-CF_2-), 107.76 (-CF_2-), 110.27 (-CHF_2-), 115.64 (-CF_2-), 127.99-128.11 (Ph), 130.23 (Ph), 130.27 (Ph), 130.96 (Ph);

^{29}Si NMR (79 MHz, $CDCl_3$, ppm): δ = 13.07 (-Si-($CH_3)_2$-), -77.95, -78.14, -78.27, -78.37 (-Si-Ph), -108.92 (SiO_4);

IR (ATR, cm^{-1}): 3073.86, 3051.55 (C-H phenyl), 2957.92, 2924.57 (C-H), 1594.33, 1430.76 (C=C phenyl), 1265.25 (Si-C), 1190.23 (C-F), 1131.40, 1091.14 (Si-O-Si), 997.30 (C-H phenyl).

EA: Anal. calcd for $C_{52}H_{50}F_8O_{14}Si_9$ (%): C, 47.91; H, 3.87; found: C, 48.09; H, 3.88.

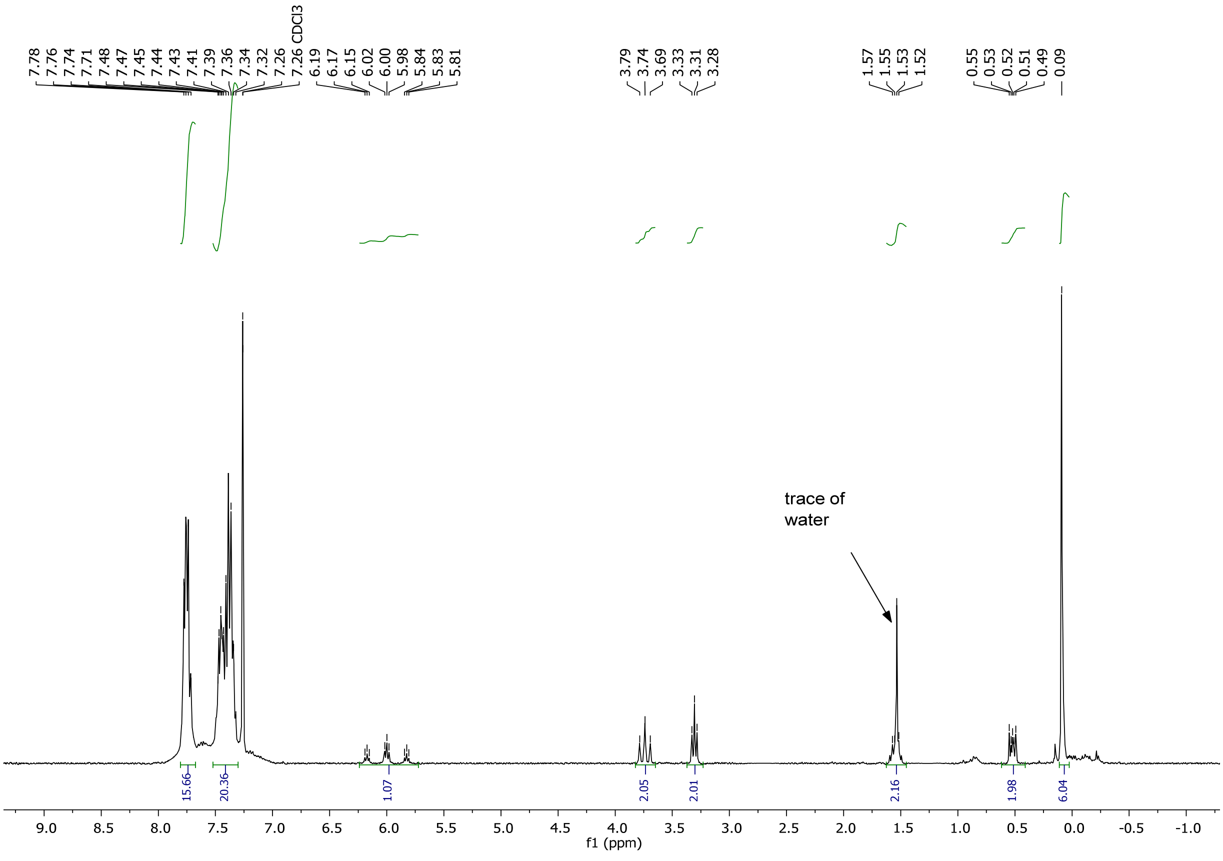

Figure S 1. ^{1}H NMR (300 MHz, $CDCl_3$) spectrum of **monoT$_8$(F1).**

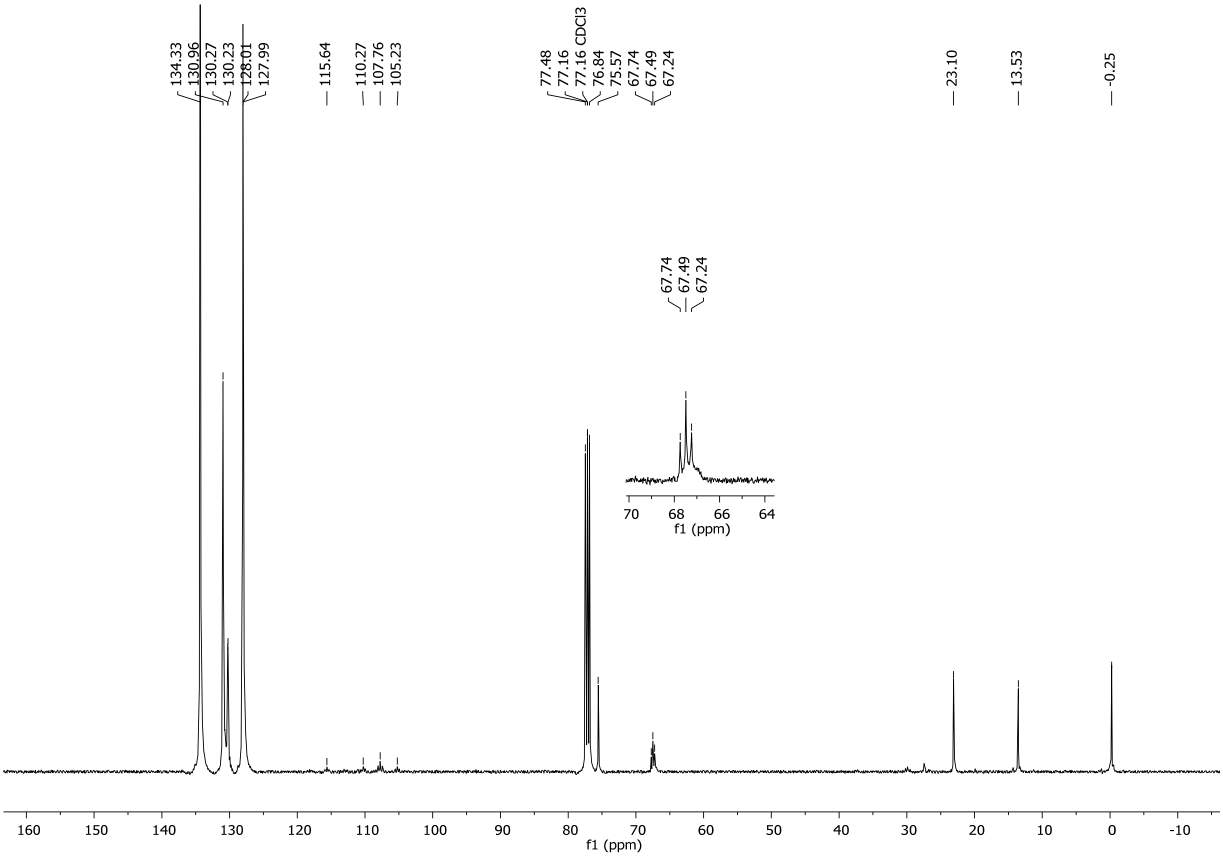

Figure S 2. ^{13}C NMR (*75.5 MHz*, $CDCl_3$) spectrum of **monoT$_8$(F1).**

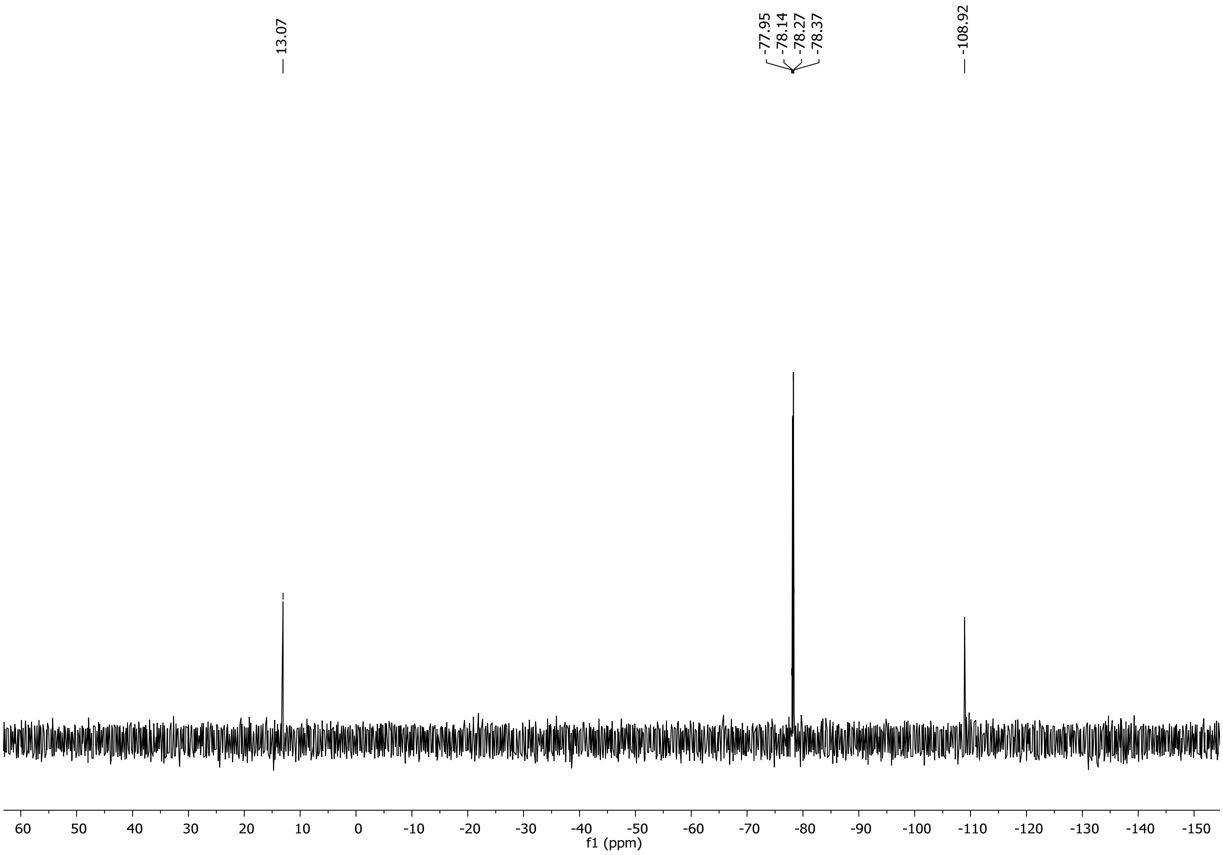

Figure S 3. ^{29}Si NMR (79.5 MHz, $CDCl_3$) spectrum of **monoT$_8$(F1).**

DDSQ-2Si(F1)

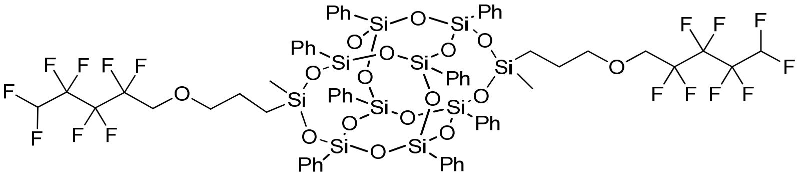

White solid. Isolated Yield 96%

^{1}H NMR (300 MHz, $CDCl_3$, ppm): δ = 0.31 (s, 6H, $Si(CH_3)_2$), 0.71-0.76 (m, 4H, Si-CH_2-), 1.68 (dd, 4H, -CH_2-), 3.41 (t, $J_{H\text{-}H}$= 6.7 Hz, 4H, -CH_2-O), 3.64 (t, $J_{H\text{-}H}$= 14.0 Hz, 4H,-O-CH_2-), 5.98 (tt, , $J_{H\text{-}H}$= 52.0, 5.6 Hz, 2H,-CH-F_2), 7.18-7.54 (m, 40H, Ph);

^{13}C NMR (101 MHz, CDCl,3, ppm): δ = -0.82 ($SiCH_3$), 12.65 (Si-CH_2-), 22.89 (-CH_2-), 67.17-67.68 (-CH_2-O-), 75.22 (O-CH_2-), 105.26 (-CF_2-), 107.77 (-CF_2-), 110.29 (-CHF_2-), 115.61 (-CF_2-), 127.85-128.00 (Ph), 130.61 (Ph), 131.11 (Ph), 131.11 (Ph), 132.03 (Ph), 134.04-134.17 (Ph);

^{29}Si NMR (79 MHz, $CDCl_3$, ppm): δ = -17.64 (-Si- CH_3-), -78.57, -79.43, -79.53, -79.62 (-Si-Ph);

IR (ATR, cm^{-1}): 3073.45 (C-H phenyl), 2923.95 (C-H), 1594.03, 1430.90 (C=C phenyl), 1262.16 (Si-C), 1174.21 (C-F), 1076.40, 1028.14 (Si-O-Si), 997.48 (C-H phenyl).

EA: Anal. calcd for $C_{66}H_{64}F_{16}O_{16}Si_{10}$ (%): C, 46.68; H, 3.80; found: C, 46.80; H, 3.81.

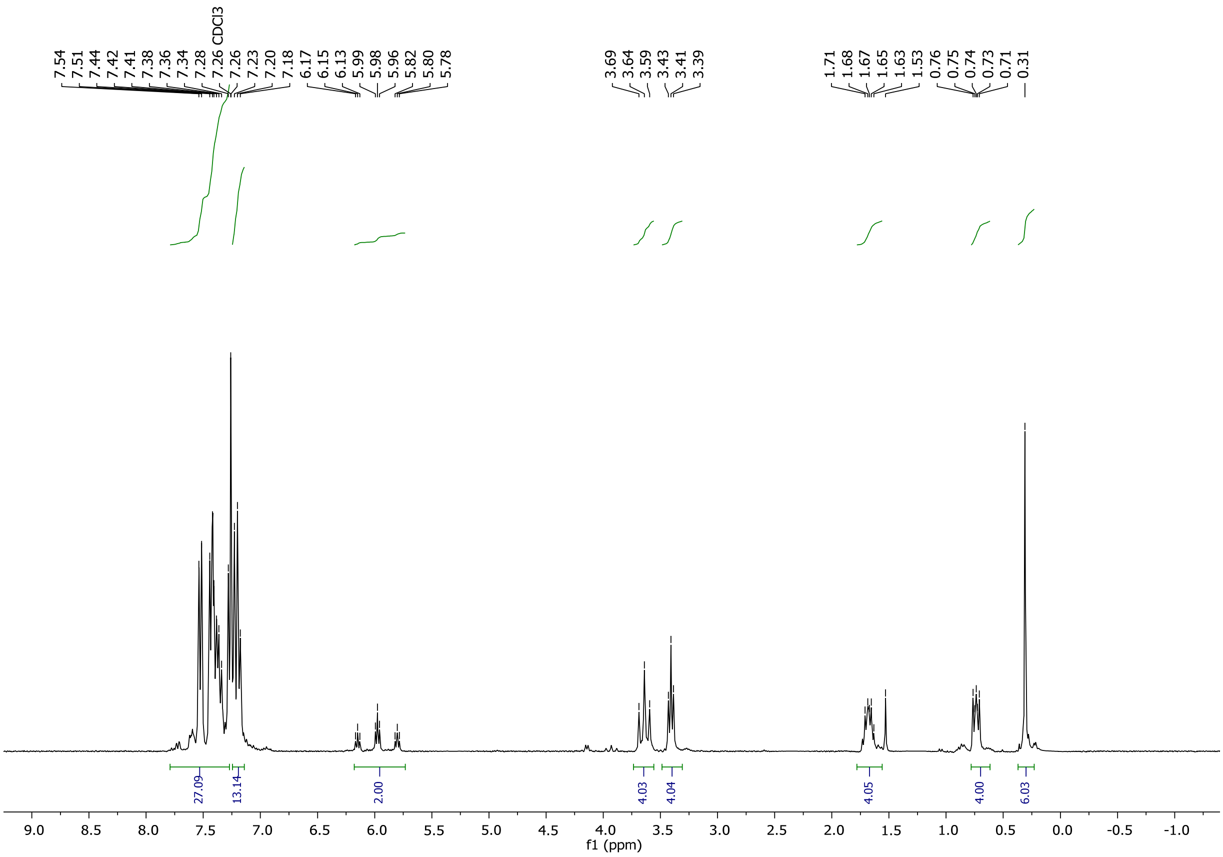

Figure S 4. ^{1}H NMR (300 MHz, $CDCl_3$) spectrum of **DDSQ-2Si(F1).**

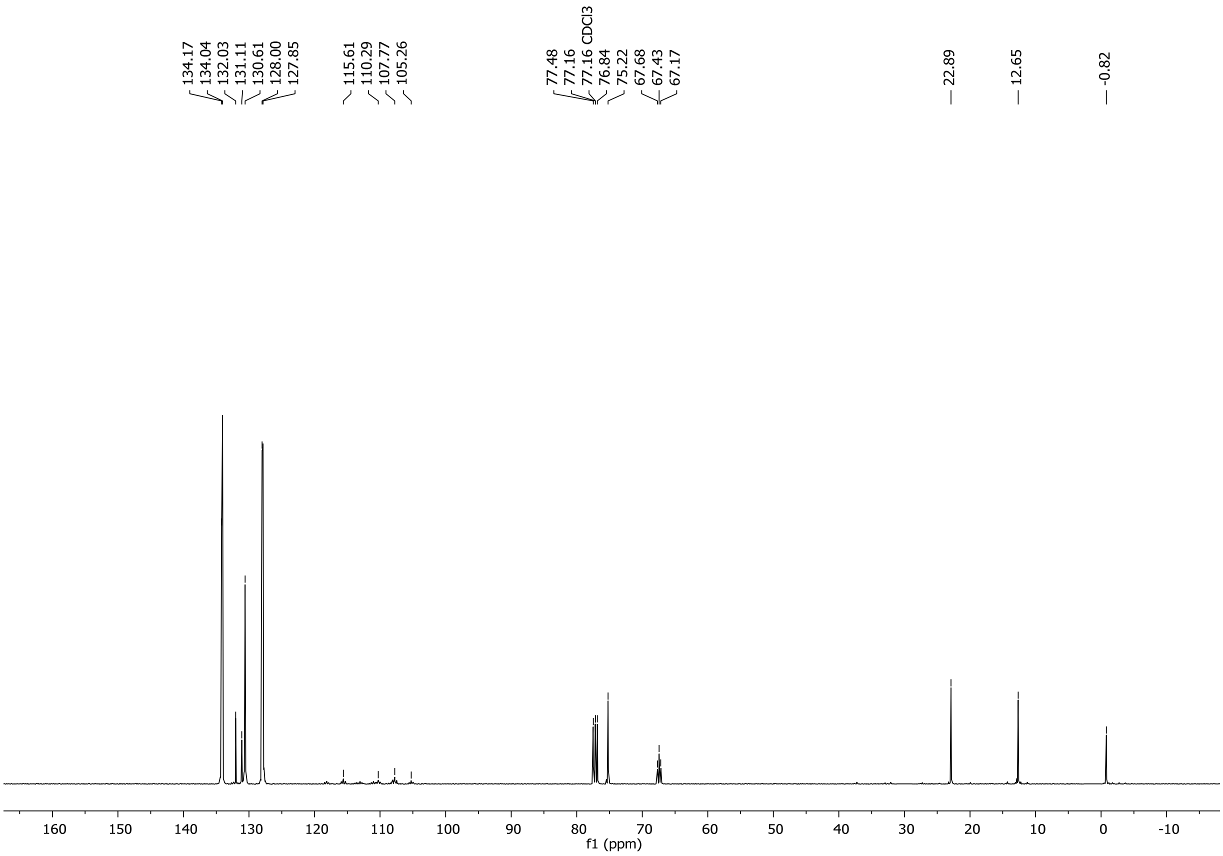

Figure S 5. ^{13}C NMR (*101 MHz*, $CDCl_3$) spectrum of **DDSQ-2Si(F1).**

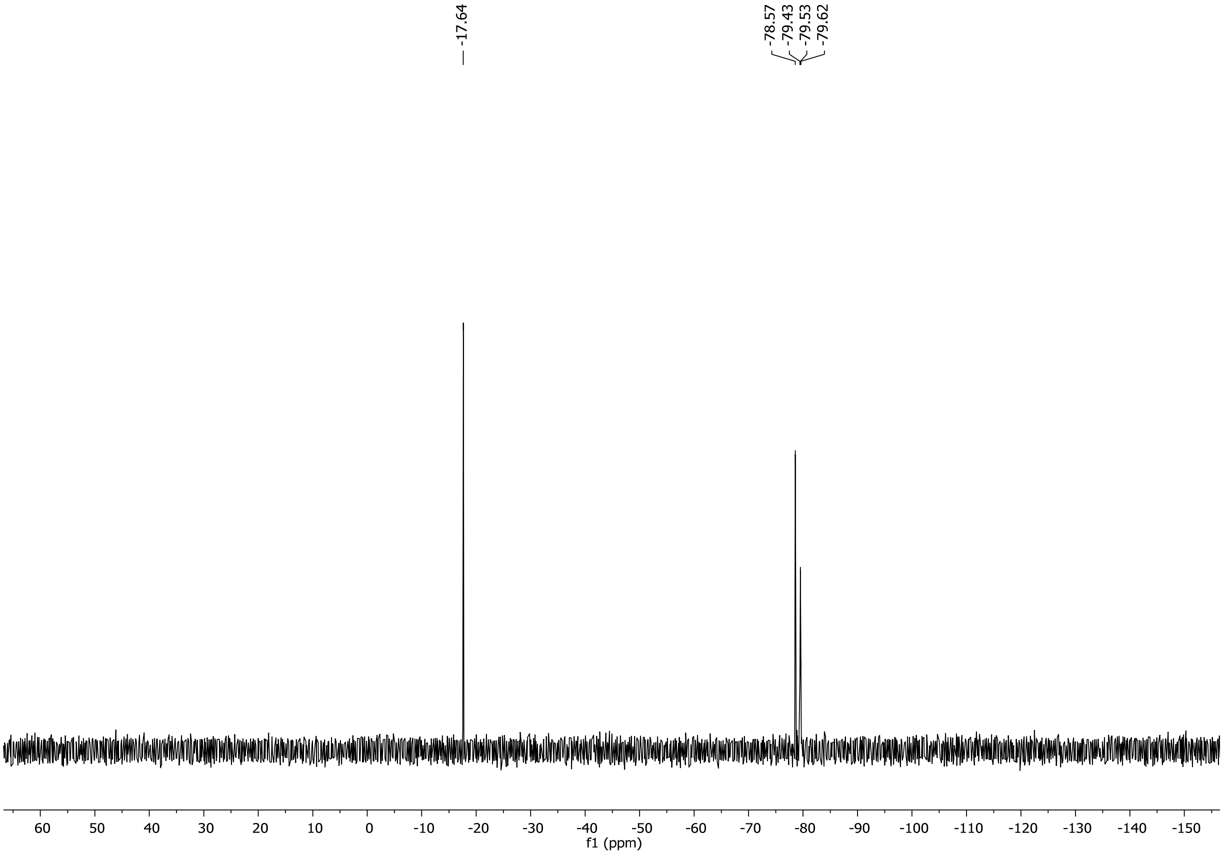

Figure S 6. ^{29}Si NMR (79.5 MHz, $CDCl_3$) spectrum of **DDSQ-2Si(F1).**

DDSQ-2Si(F2)

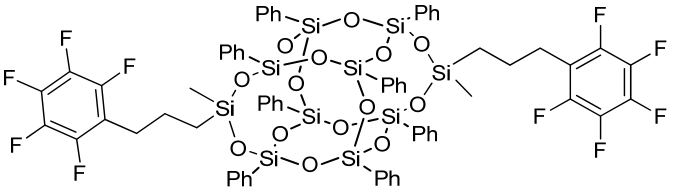

White solid. Isolated Yield 93%

^{1}H NMR (300 MHz, $CDCl_3$, ppm): δ = 0.31 (s, 6H, $Si(CH_3)_2$), 0.74-0.78 (m, 4H, Si-CH_2-), 1.68 (td, J_{H-H} = 8.1, 7.3, 3.4 Hz 4H, -CH_2-), 2.61 (t, J_{H-H} = 7.7 Hz, 4H, -CH_2-), 7.20-7.54 (m, 40H, Ph);

^{13}C NMR (101 MHz, $CDCl_3$, ppm): δ = -0.76 ($SiCH_3$), 16.67 (Si-CH_2-), 23.91 (-CH_2-), 25.56 (-CH_2-),114.92-115.28 (-C-), 127.80-127.96 (Ph), 130.57 (Ph), 131.03 (Ph), 132.00 (Ph), 134.01-134.15 (Ph), 136.15 (-CF-), 138.64 (-CF-), 140.79 (-CF-), 143.84 (-CF-), 146.28 (-CF-);

^{29}Si NMR (79 MHz, $CDCl_3$, ppm): δ = -18.38 (-Si-(CH_3)-), -78.44,-79.36, -79.42, -79.47 (-Si-Ph);

IR (ATR, cm^{-1}): 3072.92, 3051.06 (C-H phenyl), 2930.13 (C-H), 1655.26 (C=C phenyl), 1593.89, 1518.76, 1500.95 (-C-F-) 1429.98 (C=C phenyl), 1261.94 (Si-C), 1167.27 (C-F), 1027.64 (Si-O-Si), 997.19 (C-H phenyl).

EA: Anal. calcd for $C_{68}H_{58}F_{10}O_{14}Si_{10}$ (%): C, 52.02; H, 3.72; found: C, 51.91; H, 3.71.

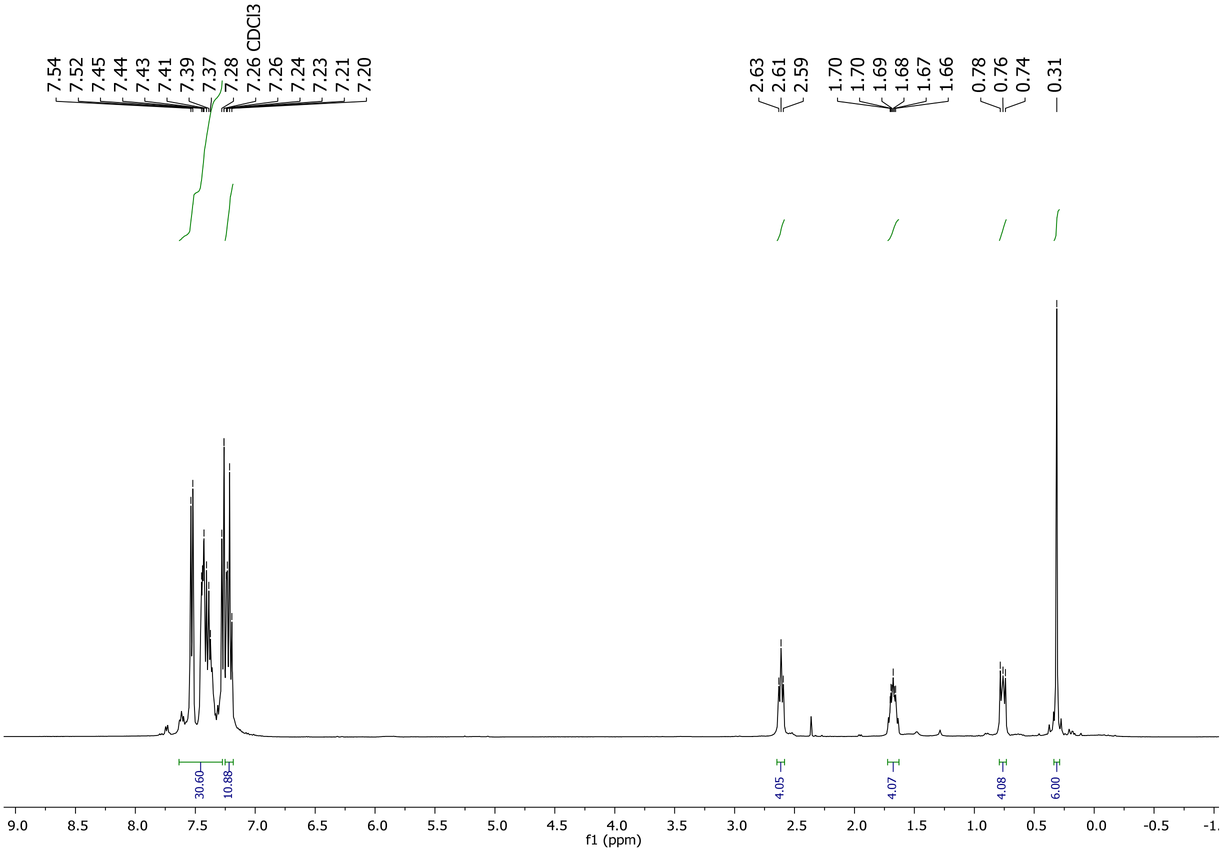

Figure S 7. ^{1}H NMR (300 MHz, $CDCl_3$) spectrum of **DDSQ-2Si(F2).**

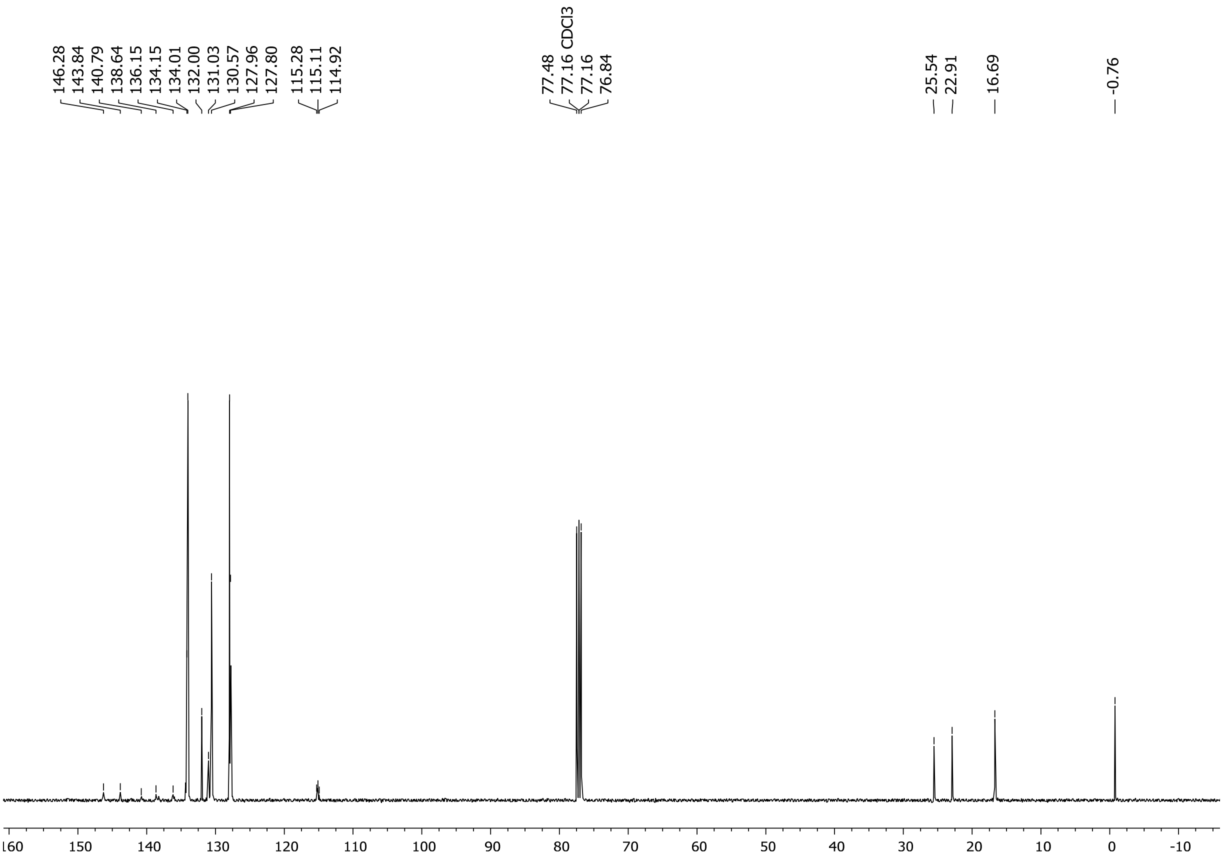

Figure S 8. ^{13}C NMR (*101 MHz*, $CDCl_3$) spectrum of **DDSQ-2Si(F2).**

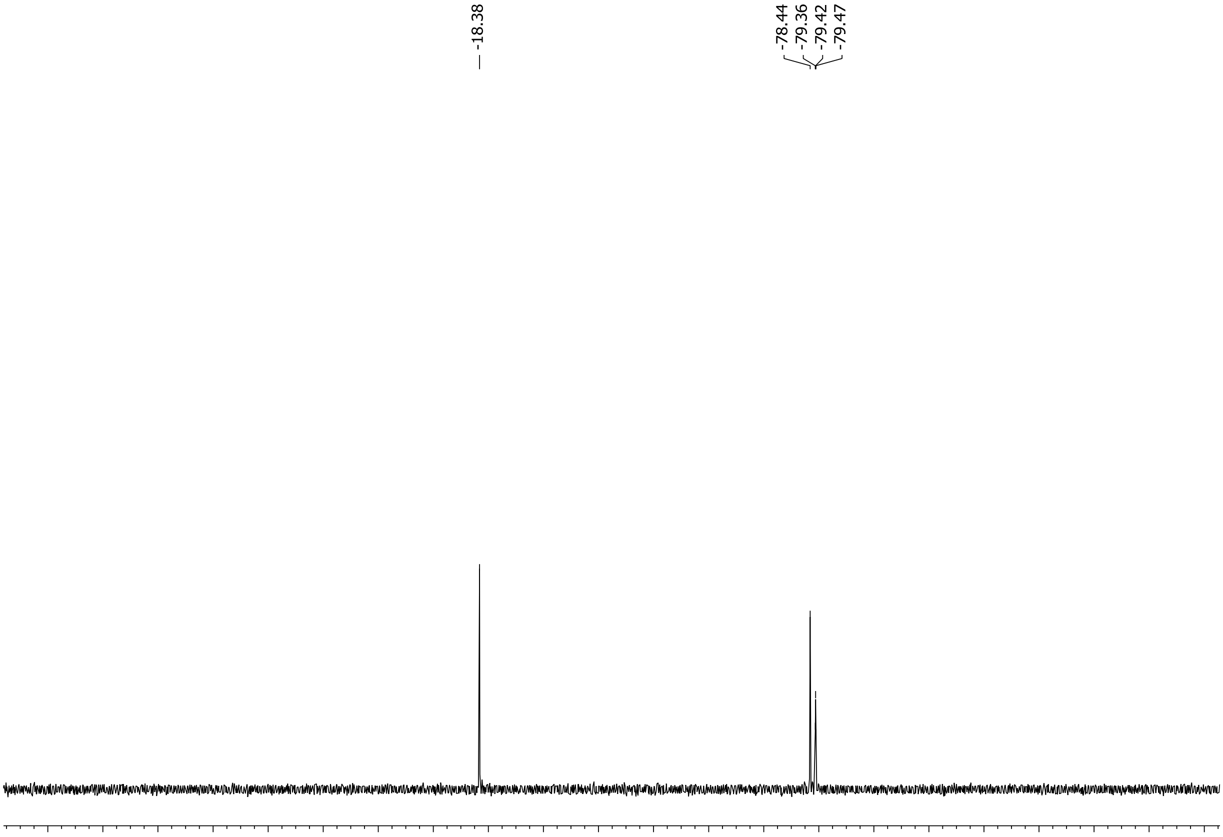

Figure S 9. ^{29}Si NMR (79.5 MHz, $CDCl_3$) spectrum of **DDSQ-2Si(F2).**

triT$_7$(F1)

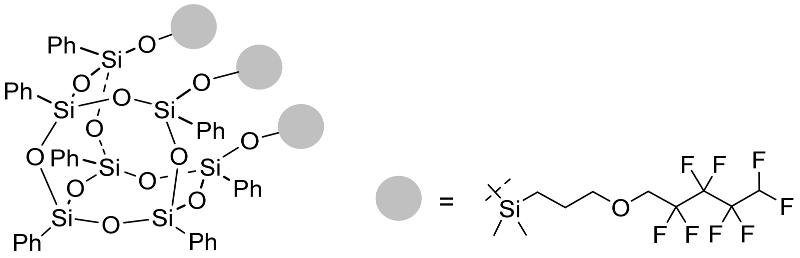

Oil. Isolated Yield 94%

^{1}H NMR (300 MHz, $CDCl_3$, ppm): δ = 0.23 (s, 18H, $Si(CH_3)_2$), 0.56-0.61 (m, 6H, Si-CH_2-), 1.59 (dq, J_{H-H} = 12.4, 7.1 Hz, 6H, -CH_2-), 3.42 (t, J_{H-H} = 6.9 Hz, 6H, -CH_2-O), 3.75 (t, J_{H-H} = 14.0Hz, 4H,-O-CH_2-), 6.00 (tt, , J_{H-H} = 52.0, 5.5Hz 3H,-CH-F_2), 7.06-7.52 (m,40H,Ph);
^{13}C NMR (101 MHz, $CDCl_3$, ppm): δ = 0.00 ($SiCH_3$), 13.46 (Si-CH_2-), 22.94 (-CH_2-), 66.81-67.48 (-CH_2-O-), 75.46 (O-CH_2-), 104.07 (-CF_2-), 107.44 (-CF_2-), 115.34 (-CHF_2-), 118.68 (-CF_2-), 127.37-128.73 (Ph), 129.92 (Ph), 130.06 (Ph), 130.79 (Ph), 132.42 (Ph), 133.69-133.77 (Ph);
^{29}Si NMR (79 MHz, $CDCl_3$, ppm): δ = 11.85 (-Si-($CH_3)_2$-), -77.36,-77.62, -77.98(-Si-Ph);
IR (ATR, cm^{-1}): 3073.78, 3051.97 (C-H phenyl), 2956.36, 2924.18 (C-H), 1594.24, 1430.50 (C=C phenyl), 1254.66 (Si-C), 1168.76 (C-F), 1107.87, 1047.64 (Si-O-Si), 997.68 (C-H phenyl).
EA: Anal. calcd for $C_{72}H_{80}F_{24}O_{15}Si_{10}$ (%): C, 44.99; H, 4.20; found: C, 45.07; H, 4.21.

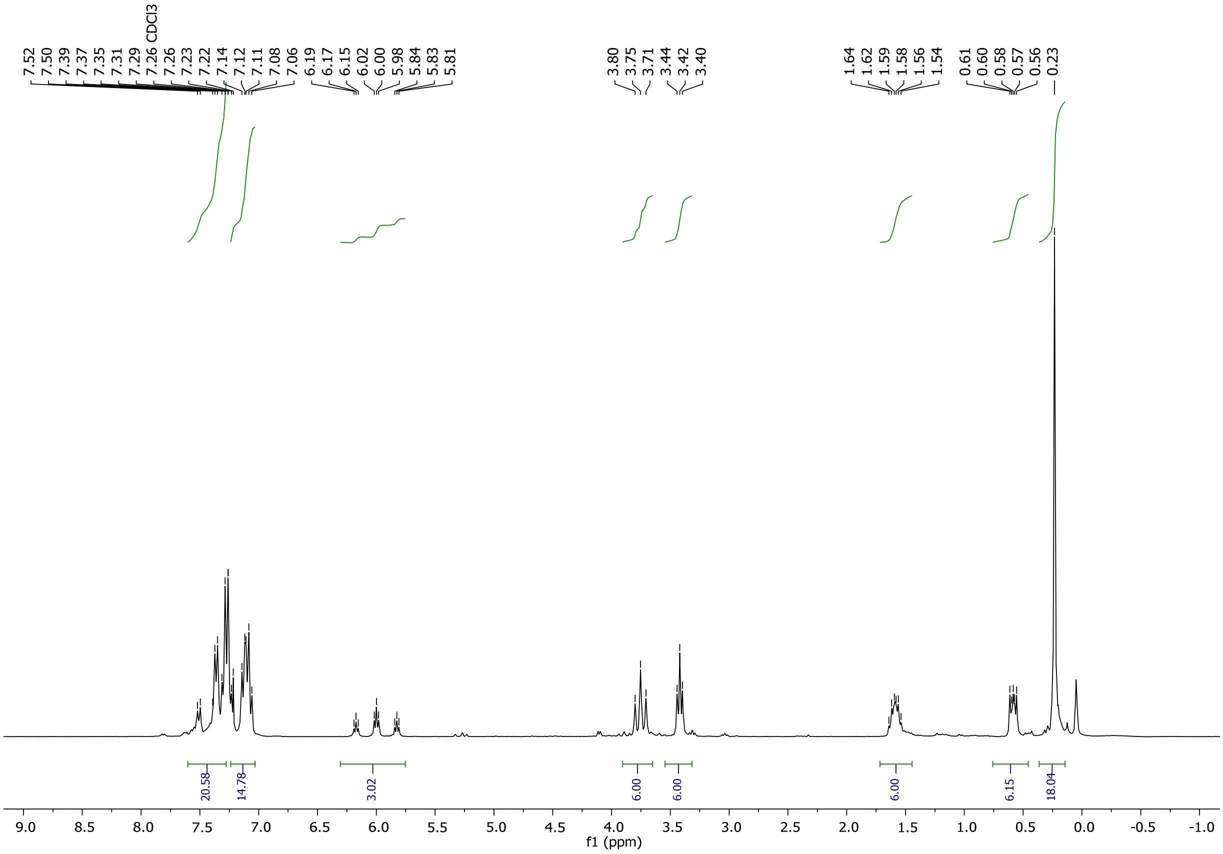

Figure S 10. ^{1}H NMR (300 MHz, $CDCl_3$) spectrum of **triT$_7$(F1).**

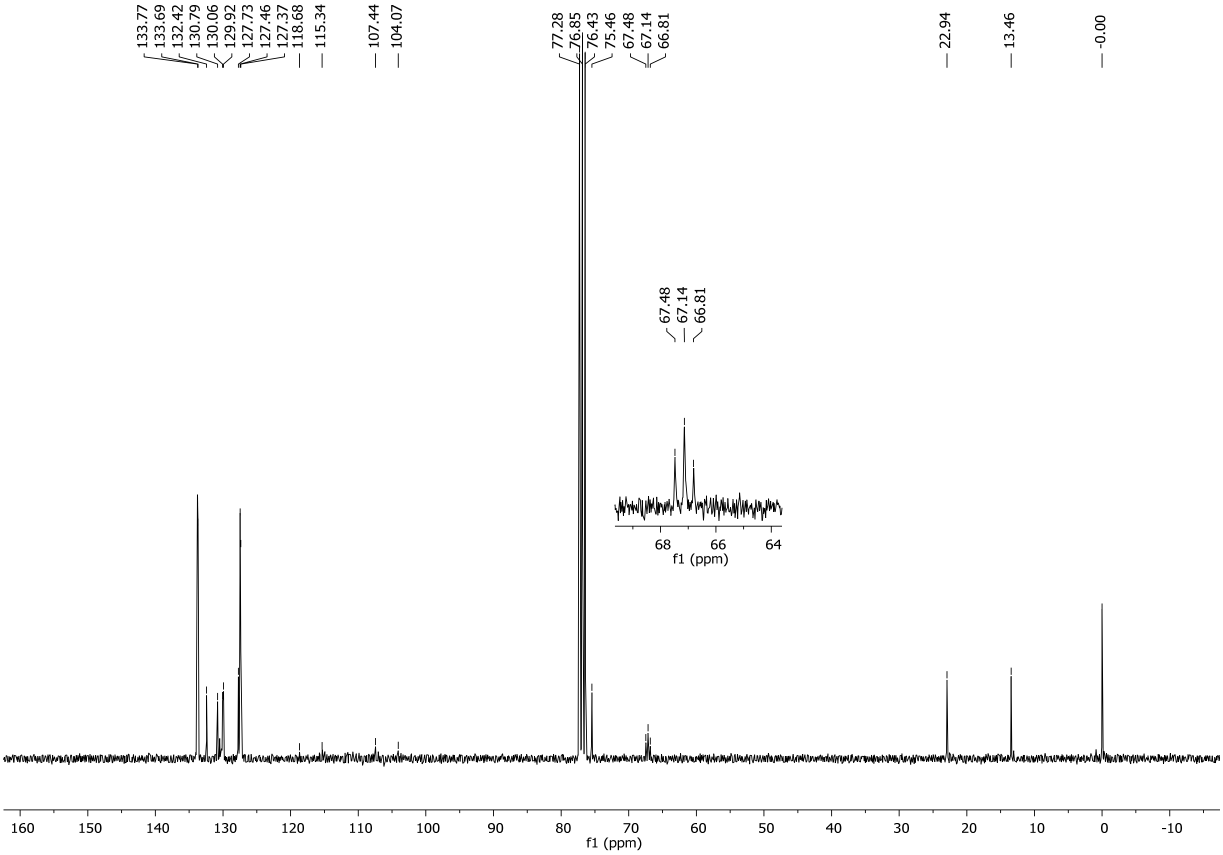

Figure S 11. ^{13}C NMR (*75.5 MHz*, $CDCl_3$) spectrum of **triT$_7$(F1).**

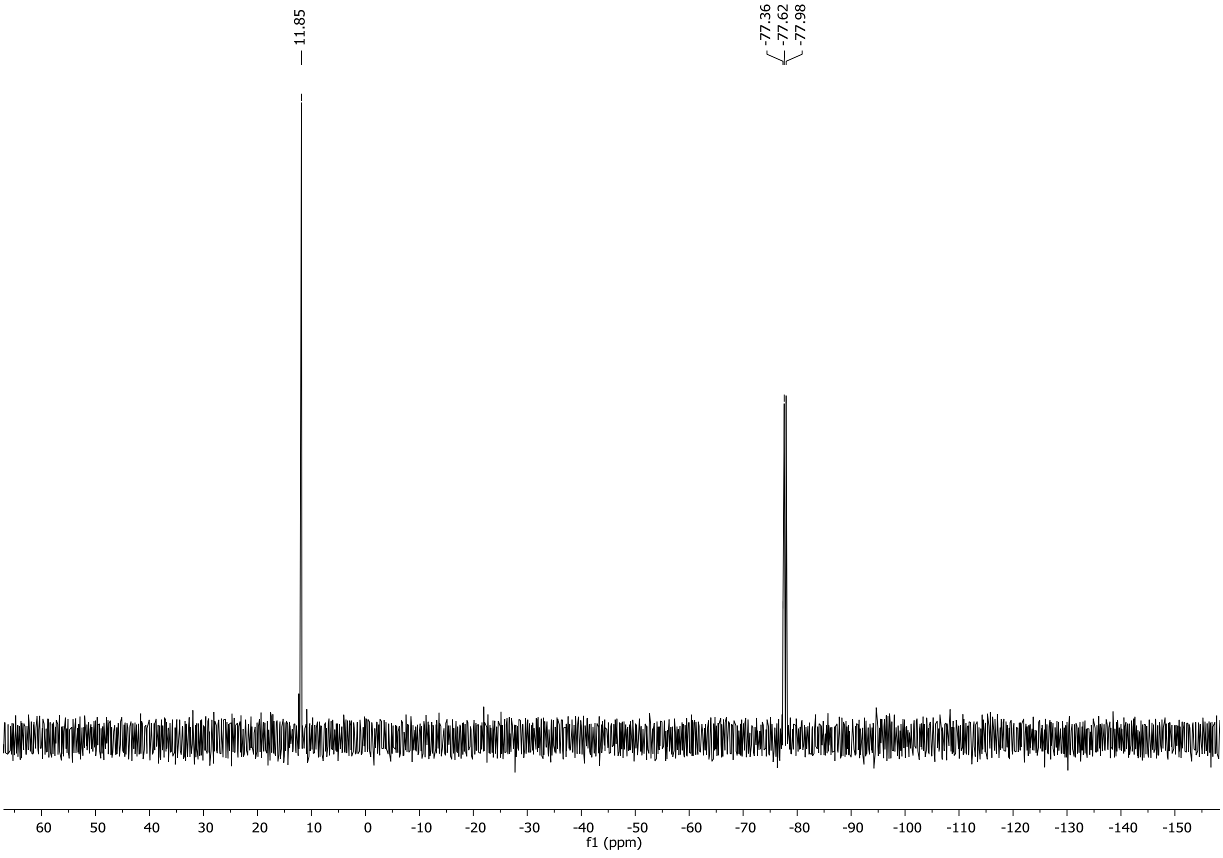

Figure S 12. ^{29}Si NMR (79.5 MHz, $CDCl_3$) spectrum of **triT$_7$(F1).**

DDSQ-4Si(F1)

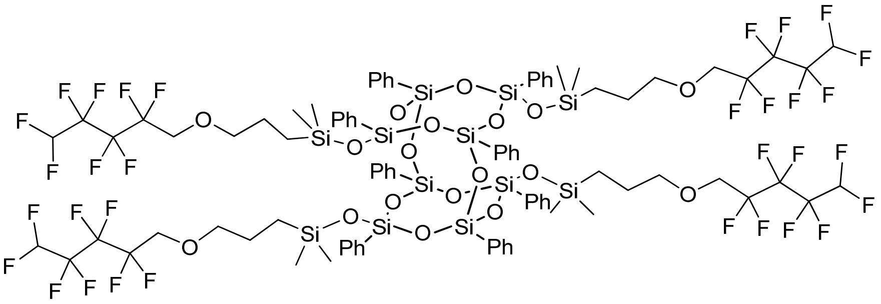

Waxy, white solid. Isolated Yield 95%, (0.186g). R_f=0.476.

^{1}H NMR (300 MHz, $CDCl_3$, ppm): δ = 0.04 (s, 24H, $Si(CH_3)_2$), 0.44 (dd, $J_{H\text{-}H}$ = 10.0, 6.2Hz 8H, Si-CH_2-), 1.32 (d, $J_{H\text{-}H}$= 5.9Hz 8H, -CH_2-), 3.06 (m, $J_{H\text{-}H}$= 6.6 Hz, 8H, -CH_2-O), 3.61 (t, $J_{H\text{-}H}$ = 14.1Hz, 8H,-O-CH_2-), 6.01 (tt,, $J_{H\text{-}H}$= 5.5Hz 4H,-CH-F_2), 7.15-7.47 (m,40H,Ph);

^{13}C NMR (101 MHz, $CDCl_3$, ppm): δ = 0.14 ($SiCH_3$), 13.88 (Si-CH_2-), 23.07 (-CH_2-), 67.08-67.58 (-CH_2-O-), 75.66 (O-CH_2-), 105.26 (-CF_2-), 107.77 (-CF_2-), 110.30 (-CHF_2-), 115.62 (-CF_2-), 127.70-128.38 (Ph), 129.19 (Ph), 130.17-130.48 (Ph), 131.67 (Ph), 133.32-134.45 (Ph);

^{29}Si NMR (79 MHz, $CDCl_3$, ppm): δ = 11.26 (-Si-$(Me)_2$-), -75.87,-78.69 (-Si-Ph);

IR (ATR, cm^{-1}): 3074.47, 3053.89 (C-H phenyl), 2958.84, 2924.78 (C-H), 1594.27, 1430.62 (C=C phenyl), 1265.36 (Si-C), 1170.28 (C-F), 1127.99, 1052.45 (Si-O-Si), 998.50 (C-H phenyl).

MALDI-ToF MS: Calcd. for $C_{88}H_{100}F_{32}Na^+O_{18}Si_{12}$: *m/z* 2411.3522 [M + Na^+]. Found: 2411.3545.

The spectroscopic data are consistent with the literature.[64]

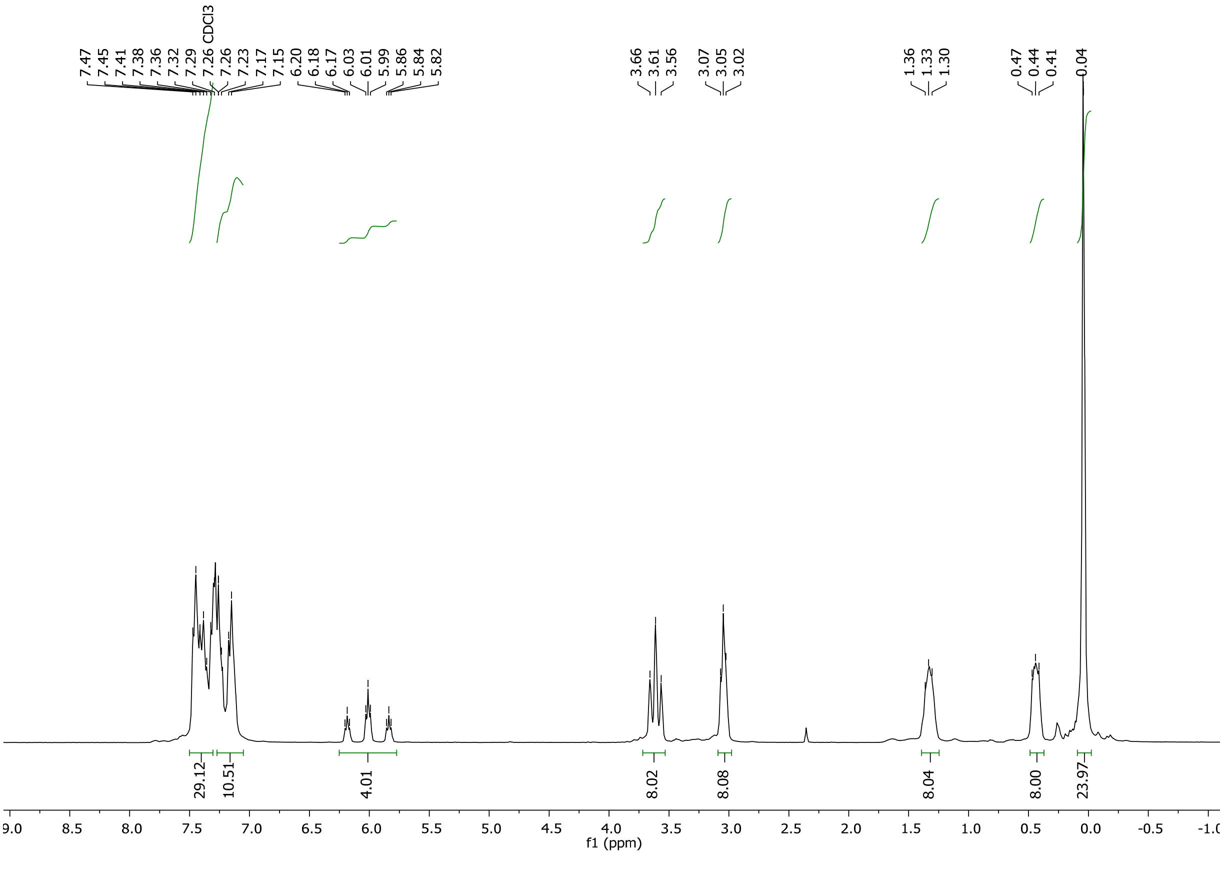

Figure S 13. ^{1}H NMR (300 MHz, $CDCl_3$) spectrum of **DDSQ-4Si(F1.)**

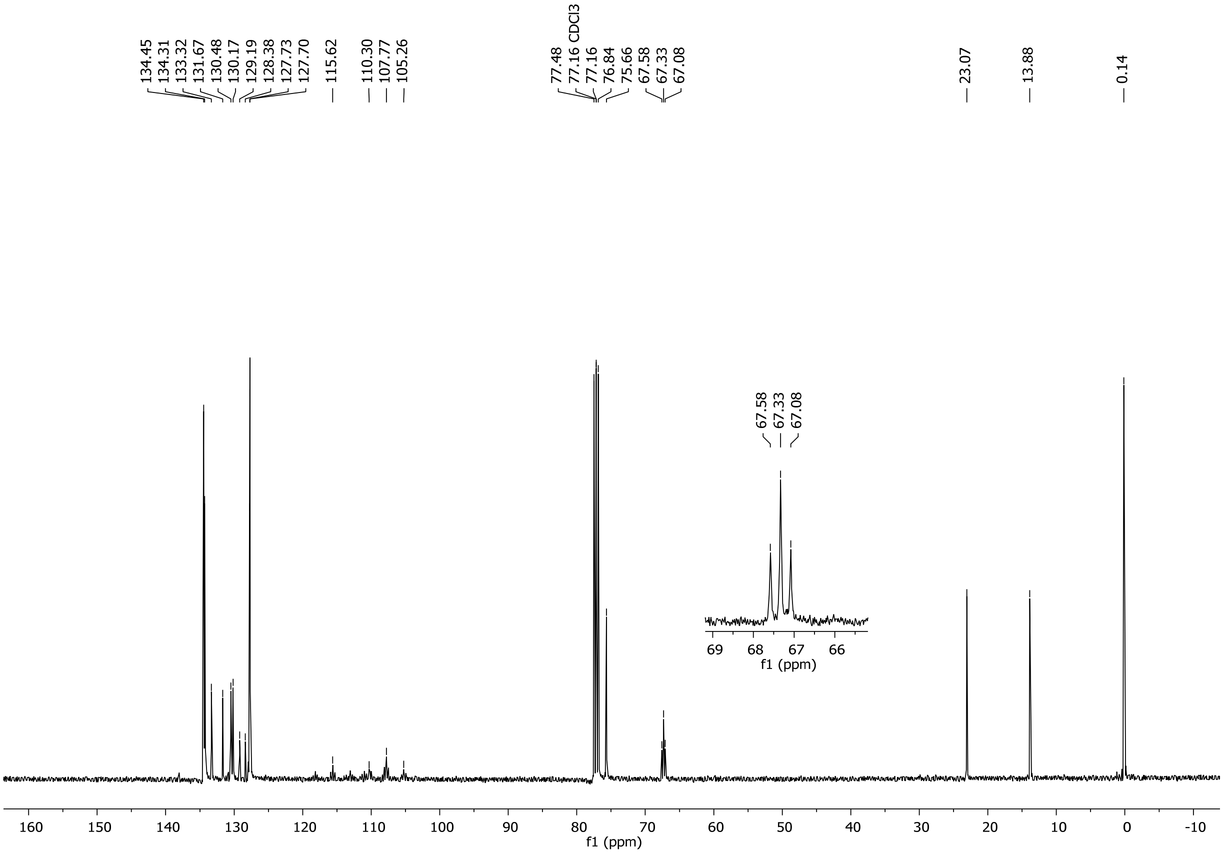

Figure S 14. ^{13}C NMR (*101 MHz*, $CDCl_3$) spectrum of **DDSQ-4Si(F1).**

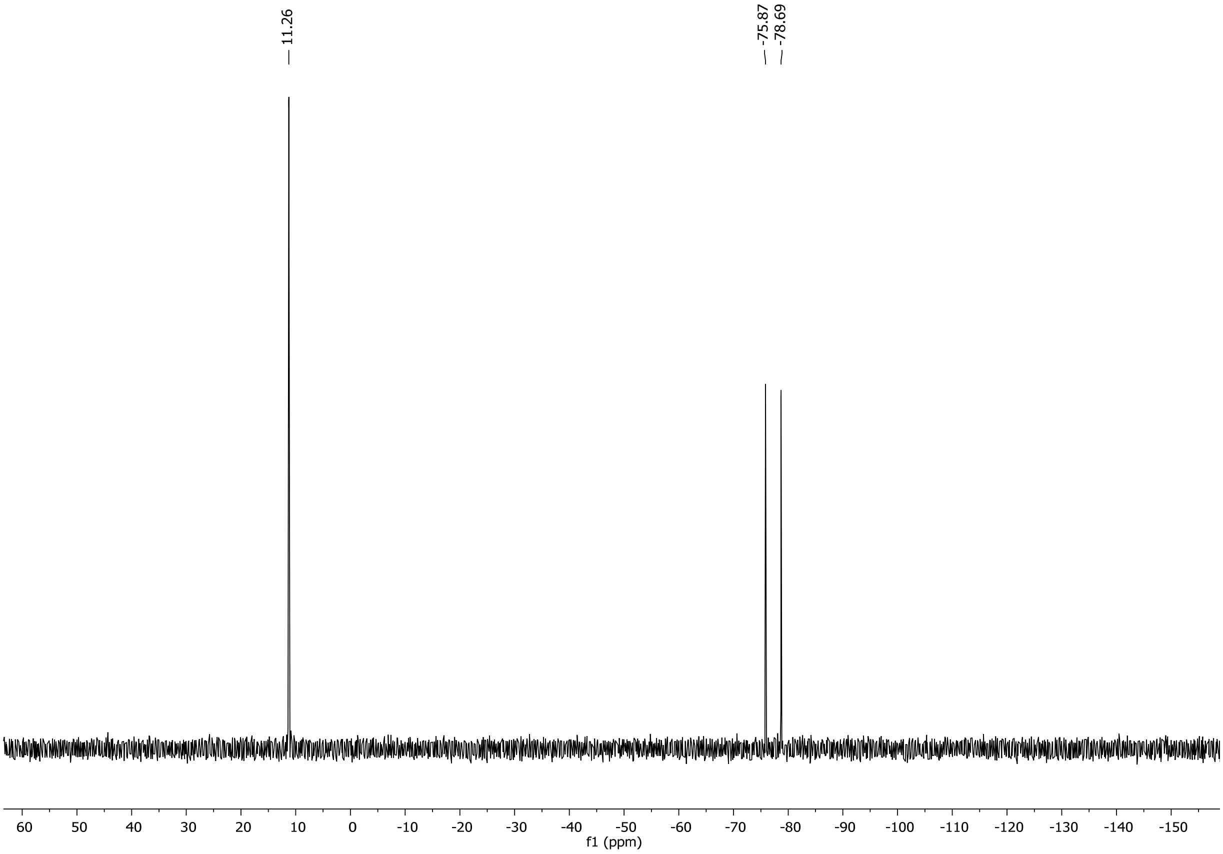

Figure S 15. ^{29}Si NMR (79.5 MHz, $CDCl_3$) spectrum of **DDSQ-4Si(F1).**

octaT_8(F1)

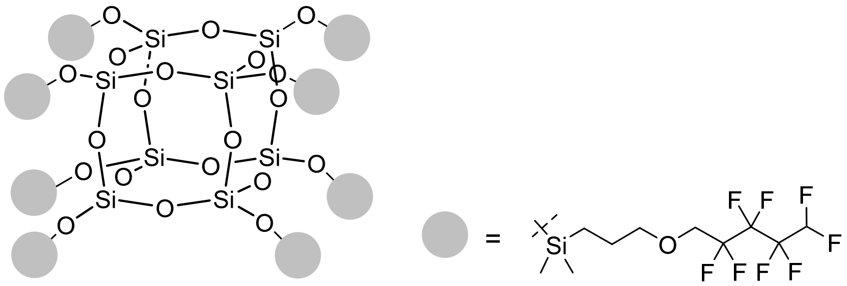

Oil. Isolated Yield 93%

^{1}H NMR (300 MHz, $CDCl_3$, ppm): δ = 0.14 (s, 48H, $Si(CH_3)_2$), 0.56-0.62 (m, 16H, Si-CH_2-), 1.64 (dq, $J_{H\text{-}H}$ = 12.7, 6.8 Hz 16H, -CH_2-), 3.54 (t, $J_{H\text{-}H}$ = 6.7 Hz, 16H, -CH_2-O), 3.9 (t, $J_{H\text{-}H}$ = 14.1Hz, 16H,-O-CH_2-), 6.05 (tt, $J_{H\text{-}H}$ = 52.0, 5.5Hz 8H,-CH-F_2);
^{13}C NMR (101 MHz, $CDCl_3$, ppm): δ = -0.47 ($SiCH_3$), 13.44 (Si-CH_2-), 23.11 (-CH_2-), 67.23-67.90 (-CH_2-O-), 75.71 (O-CH_2-), 104.37 (-CF_2-), 107.75 (-CF_2-), 111.09 (-CHF_2-), 115.66 (-CF_2-);
^{29}Si NMR (79 MHz, $CDCl_3$, ppm): δ = 13.00 (-Si-(Me)$_2$-), -109.00 (-Si-);
IR (ATR, cm^{-1}): 2958.18, 2927.81, 2880.96 (C-H), 1255.21 (Si-C), 1164.63 (C-F), 1072.06 (Si-O-Si). The spectroscopic data are consistent with the literature.[69]

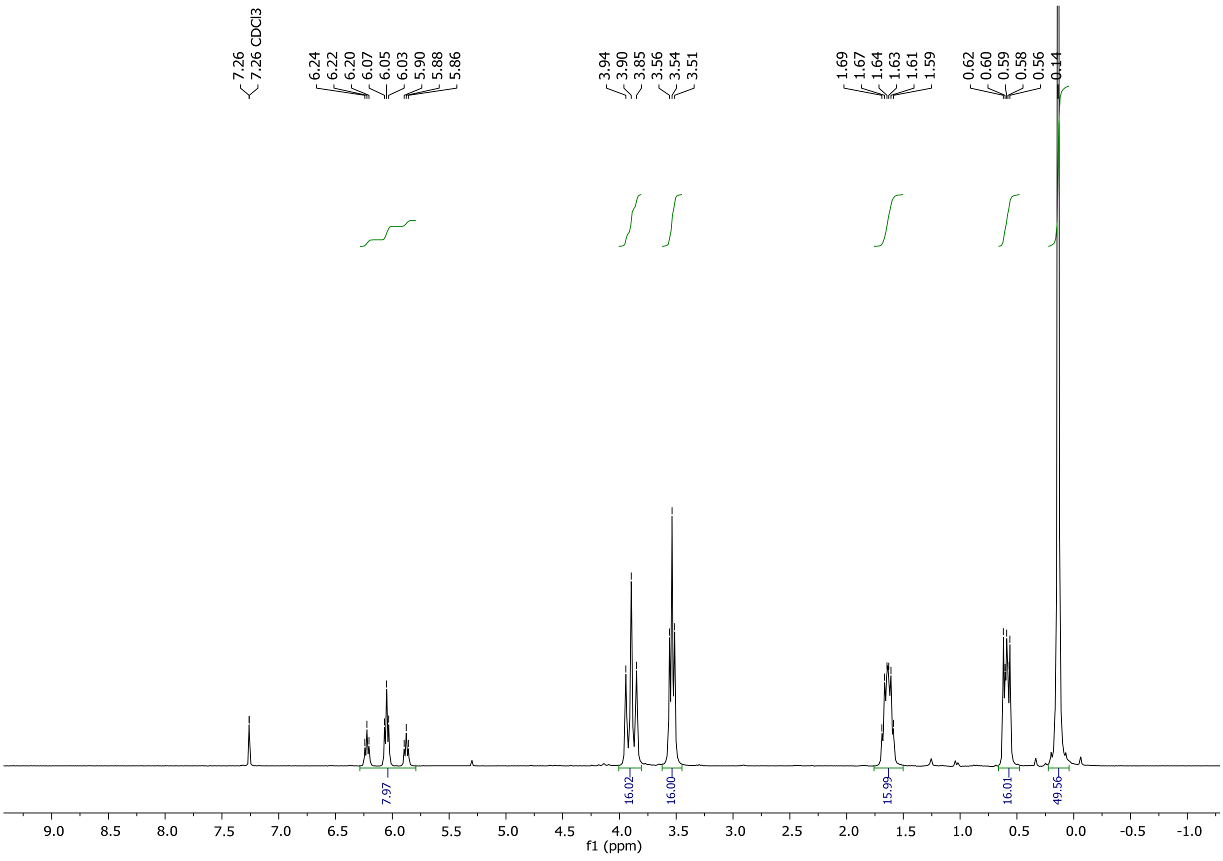

Figure S 16. ^{1}H NMR (300 MHz, $CDCl_3$) spectrum of **octaT_8(F1).**

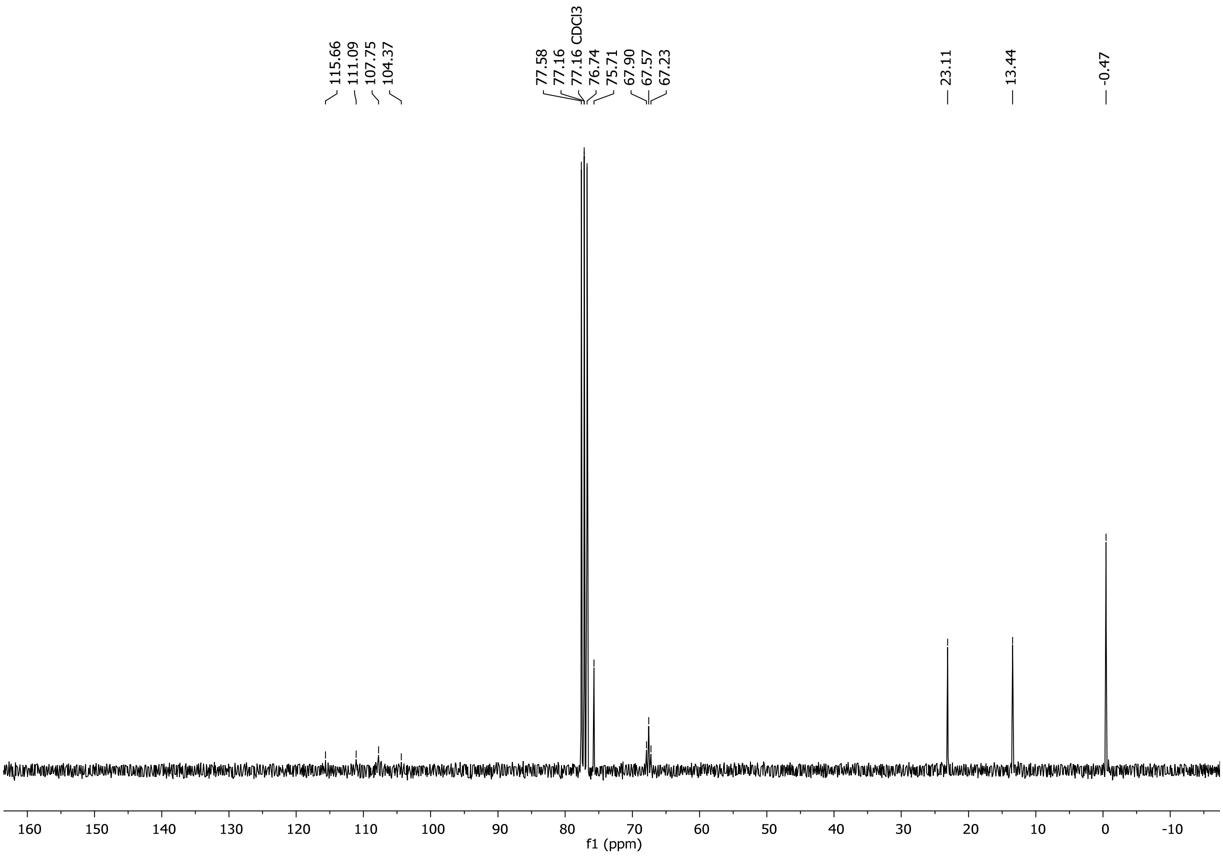

Figure S 17. ^{13}C NMR (*75.5 MHz*, $CDCl_3$) spectrum of **octaT_8(F1).**

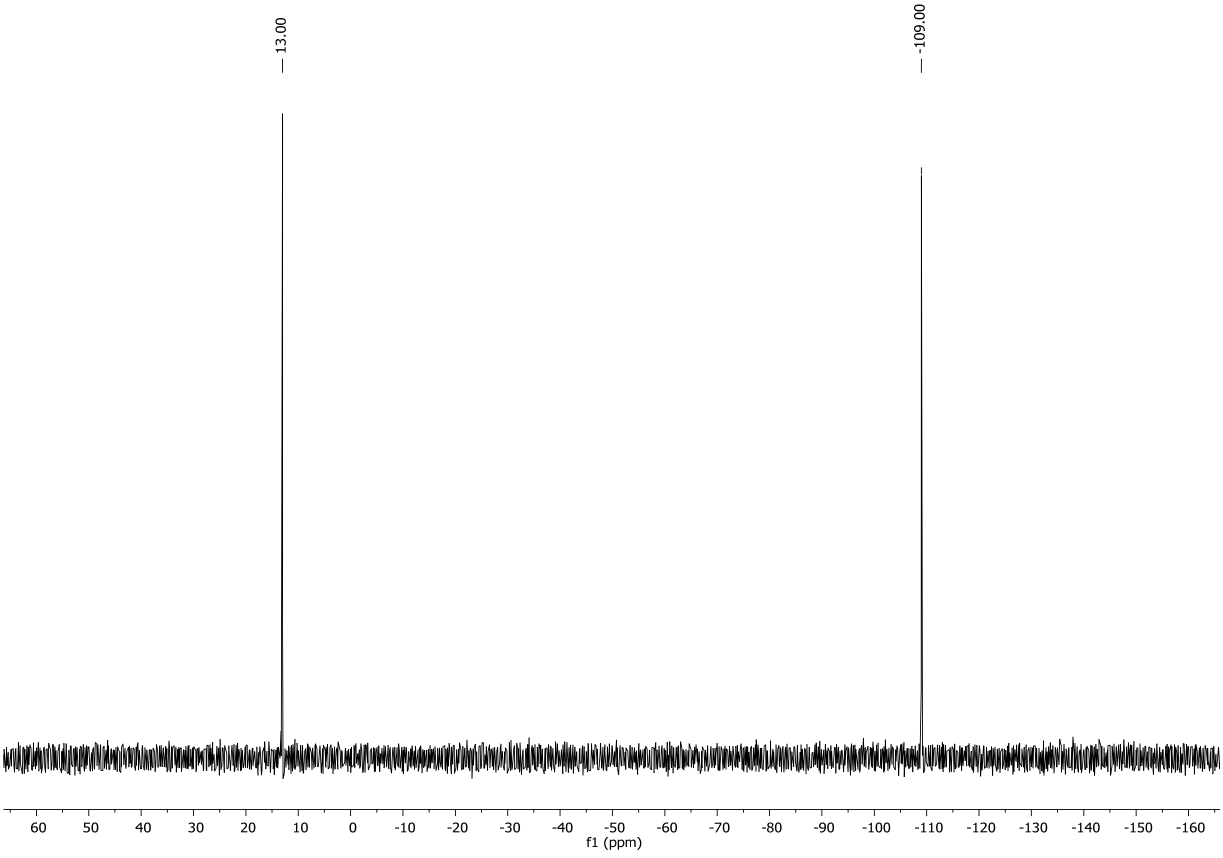

Figure S 18. ^{29}Si NMR (79.5 MHz, $CDCl_3$) spectrum of **octaT_8(F1).**

monoT$_8$(F3)

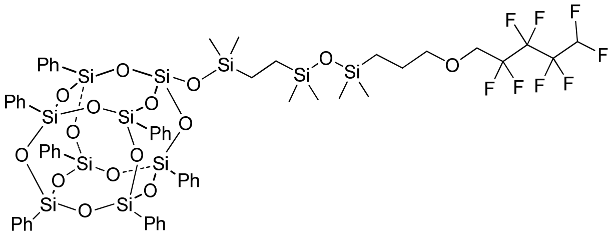

Waxy, white solid. Isolated Yield 93%

^{1}H NMR (300 MHz, $CDCl_3$, ppm): δ = -0.07(s, 6H, $Si(CH_3)_2$), -0.01 (s, 6H, $Si(CH_3)_2$, 0.08 (s, 6H, $Si(CH_3)_2$), 0.09-0.13 (m, 2H, Si-CH_2-), 0.39-0.44 (m, 2H, -CH_2-Si-), 0.47-0.57 (m, 2H, Si-CH_2-), 1.52-1.65 (m, 2H, -CH_2-), 3.51 (dt, J_{H-H}= 23.6, 6.8 Hz, 2H, -CH_2-O), 3.89 (dt, J_{H-H}= 19.0, 13.9, Hz, 2H, -O-CH_2-), 6.06 (tq, J_{H-H}= 52.00, 6.3 Hz,, 1H,-CH-F_2), 7.35-7.46, 7.73-7.77 (m, 35H, Ph);
^{13}C NMR (101 MHz, $CDCl_3$, ppm): δ = -1.06 (-$Si(CH_3)_2$), -0.48 ($Si(CH_3)_2$), 0.23 (-$Si(CH_3)_2$), 9.02 (-Si-CH_2-), 9.24 (-CH_2-Si), 13.92 (-Si-CH_2-), 23.29 (-CH_2-), 67.19-67.72 (-CH_2-O-), 75.76 (O-CH_2-), 105.11 (-CF_2-), 107.62 (-CF_2-), 110.16 (-CHF_2-), 115.53 (-CF_2-), 127.67-127.83 (Ph), 130.16 (Ph), 130.19 (Ph), 130.75 (Ph), 134.00-134.19 (Ph);
^{29}Si NMR (79 MHz, $CDCl_3$, ppm): δ = 7.24 (-Si-($CH_3)_2$-), 8.72 (-Si-($CH_3)_2$-), 13.66 (-Si-($CH_3)_2$), -78.37, -78.32, -78.37 (-Si-Ph), -108.90 (-SiO_4);
IR (ATR, cm^{-1}): 3073.94, 3051.76 (C-H phenyl), 2956.72, 2924.13 (C-H), 1594.45, 1430.71 (C=C phenyl), 1254.19 (Si-C), 1131.06 (C-F), 1043.72 (Si-O-Si), 997.21 (C-H phenyl).
EA: Anal. calcd for $C_{58}H_{66}F_8O_{15}Si_{11}$ (%): C, 47.58; H, 4.54; found: C, 47.46; H, 4.53.

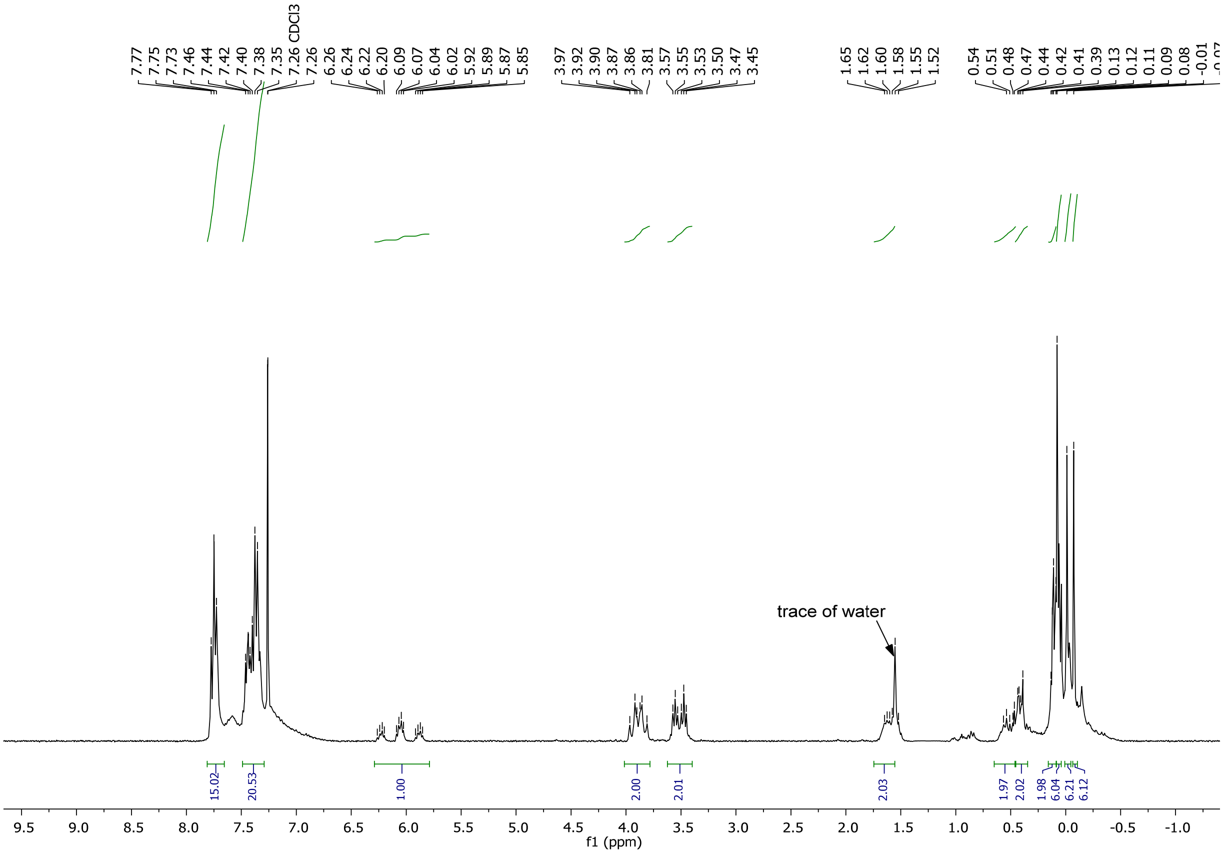

Figure S 19. ^{1}H NMR (300 MHz, $CDCl_3$) spectrum of **monoT$_8$(F3).**

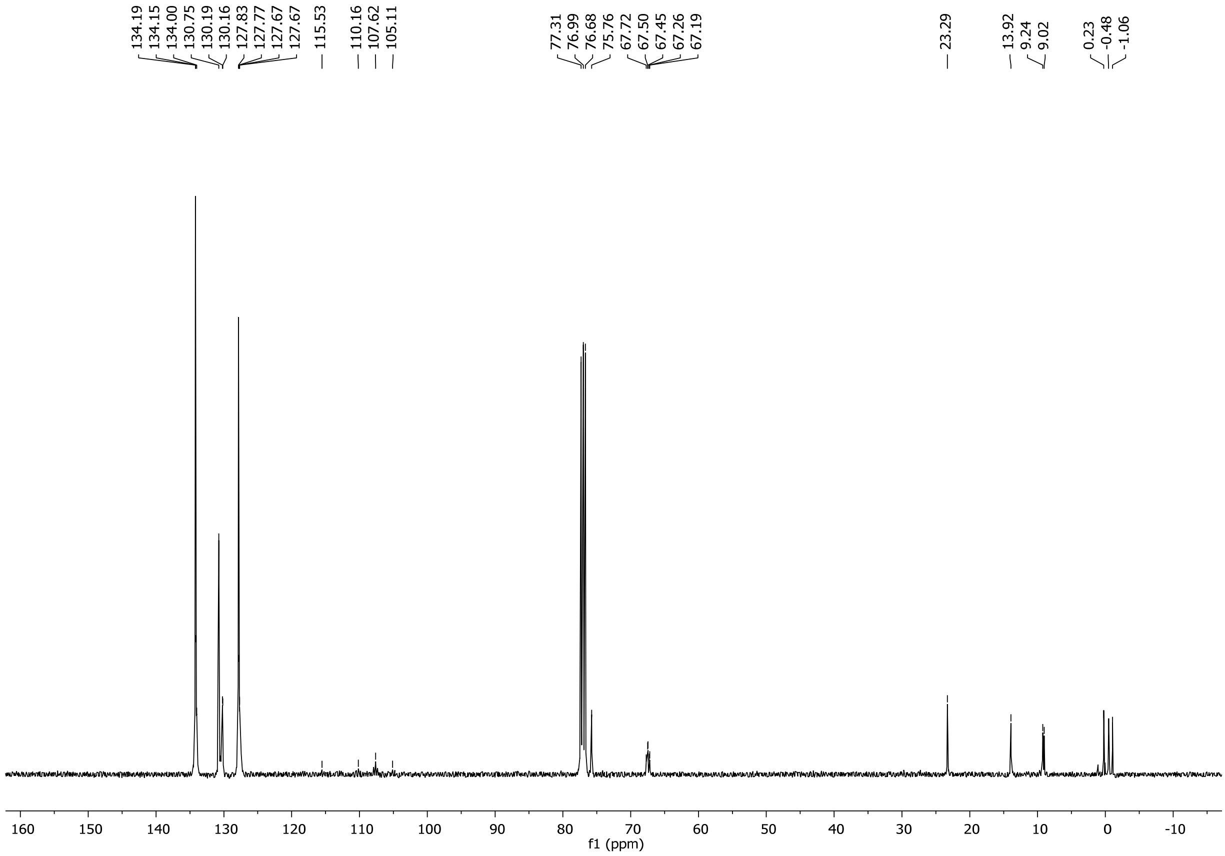

Figure S 20. ^{13}C NMR (*101 MHz*, $CDCl_3$) spectrum of **monoT$_8$(F3).**

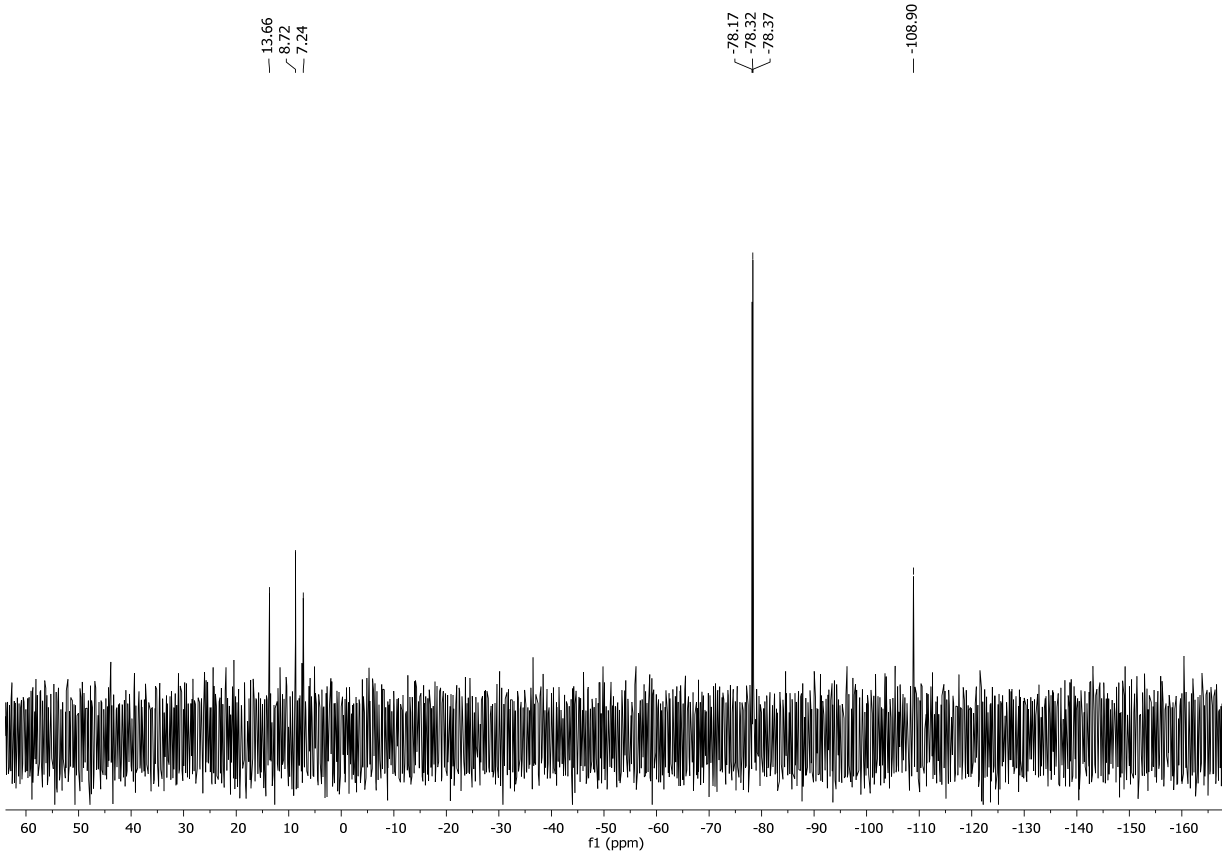

Figure S 21. ^{29}Si NMR (79.5 MHz, $CDCl_3$) spectrum of **monoT$_8$(F3).**

DDSQ-2Si(F3)

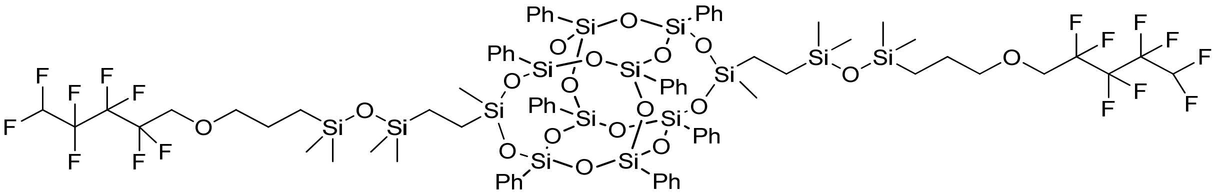

White solid. Isolated Yield 91%

^{1}H NMR (300 MHz, $CDCl_3$, ppm): δ = -0.09 (d, $J_{H\text{-}H}$ = 2.8 Hz, 12H, $Si(CH_3)_2$), -0.03 (d, $J_{H\text{-}H}$=2.6 Hz, 12H, $Si(CH_3)_2$, 0.07-0.15 (m, 4H, $Si(CH_3)_2$), 0.30 (d, $J_{H\text{-}H}$ = 2.8 Hz, 6H, Si-CH_2-), 0.40-0.54 (m, 4H, -CH_2-Si-),0.58-0.62 (m, 4H, Si-CH_2-), 1.51-1.56 (m, 4H, -CH_2-), 3.41-3.58 (m, 4H, -CH_2-O), 3.84 (t, $J_{H\text{-}H}$ = 13.9 Hz, 4H,-O-CH_2-), 6.04 (tt, $J_{H\text{-}H}$ = 51.07, 6.0 Hz,, 2H,-CH-F_2), 7.18-7.57 (m, 40H, Ph);

^{13}C NMR (101 MHz, $CDCl_3$, ppm): δ = -1.56 (-$Si(CH_3)_2$), -0.39 ($Si(CH_3)_2$), 0.35 (-$Si(CH_3)_2$), 8.43 (-Si-CH_2-), 9.25 (-CH_2-Si), 14.09 (-Si-CH_2-), 23.45 (-CH_2-), 67.35-67.85 (-CH_2-O-), 75.89 (O-CH_2-), 105.31 (-CF_2-), 107.82 (-CF_2-), 110.34 (-CHF_2-), 115.70 (-CF_2-), 127.80-127.92 (Ph), 130.47 (Ph), 131.32 (Ph), 132.38 (Ph), 134.13-134.23 (Ph);

^{29}Si NMR (79 MHz, $CDCl_3$, ppm): δ = 8.69 (-Si-($CH_3)_2$-), 7.33 (-Si-($CH_3)_2$-), -17.08 (-Si-($CH_3)_2$), -78.69, -79.64 (-Si-Ph);

IR (ATR, cm^{-1}): 3073.49 (C-H phenyl), 2923.95 (C-H), 1594.23, 1430.48 (C=C phenyl), 1262.19 (Si-C), 1172.16 (C-F), 1028.14 (Si-O-Si), 997.48 (C-H phenyl).

EA: Anal. calcd for $C_{78}H_{96}F_{16}O_{18}Si_{14}$ (%): C, 46.41; H, 4.79; found: C, 46.50; H, 4.80.

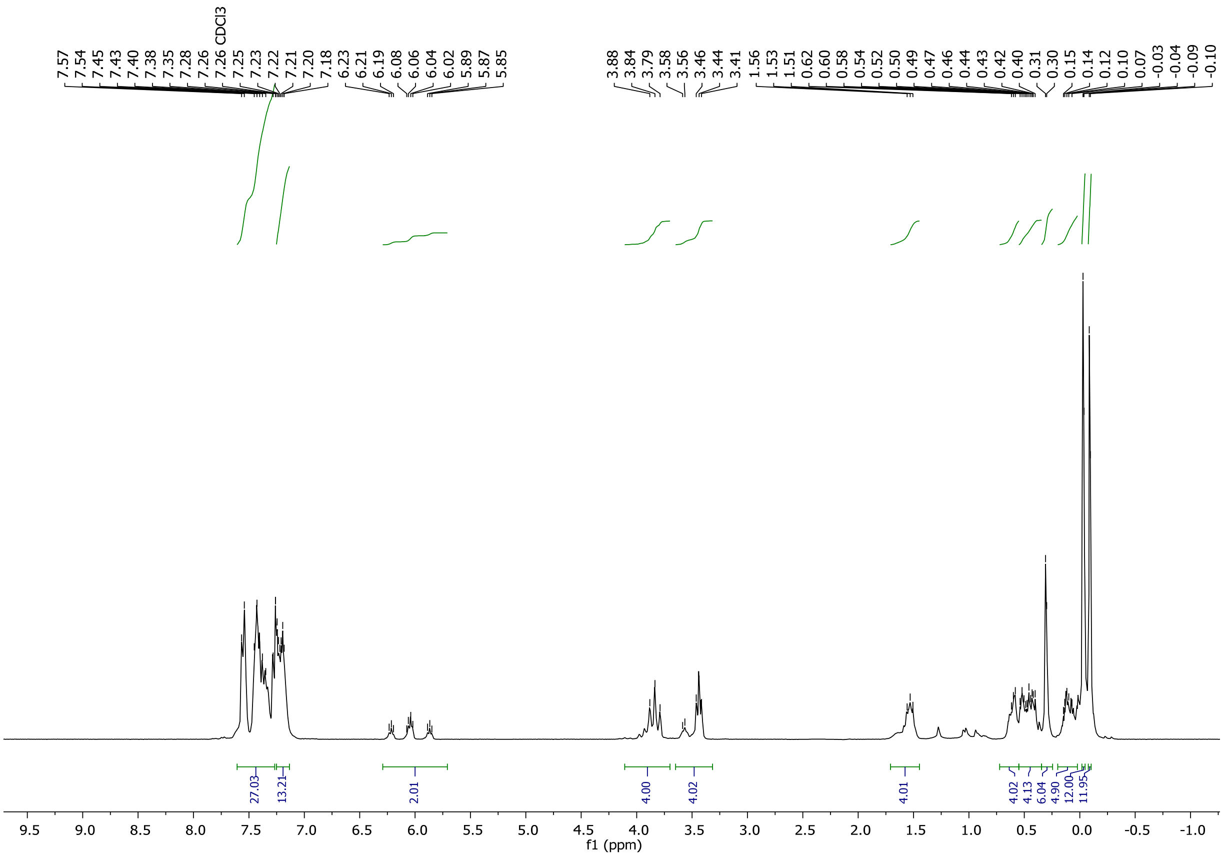

Figure S 22. ^{1}H NMR (300 MHz, $CDCl_3$) spectrum of **DDSQ-2Si(F3).**

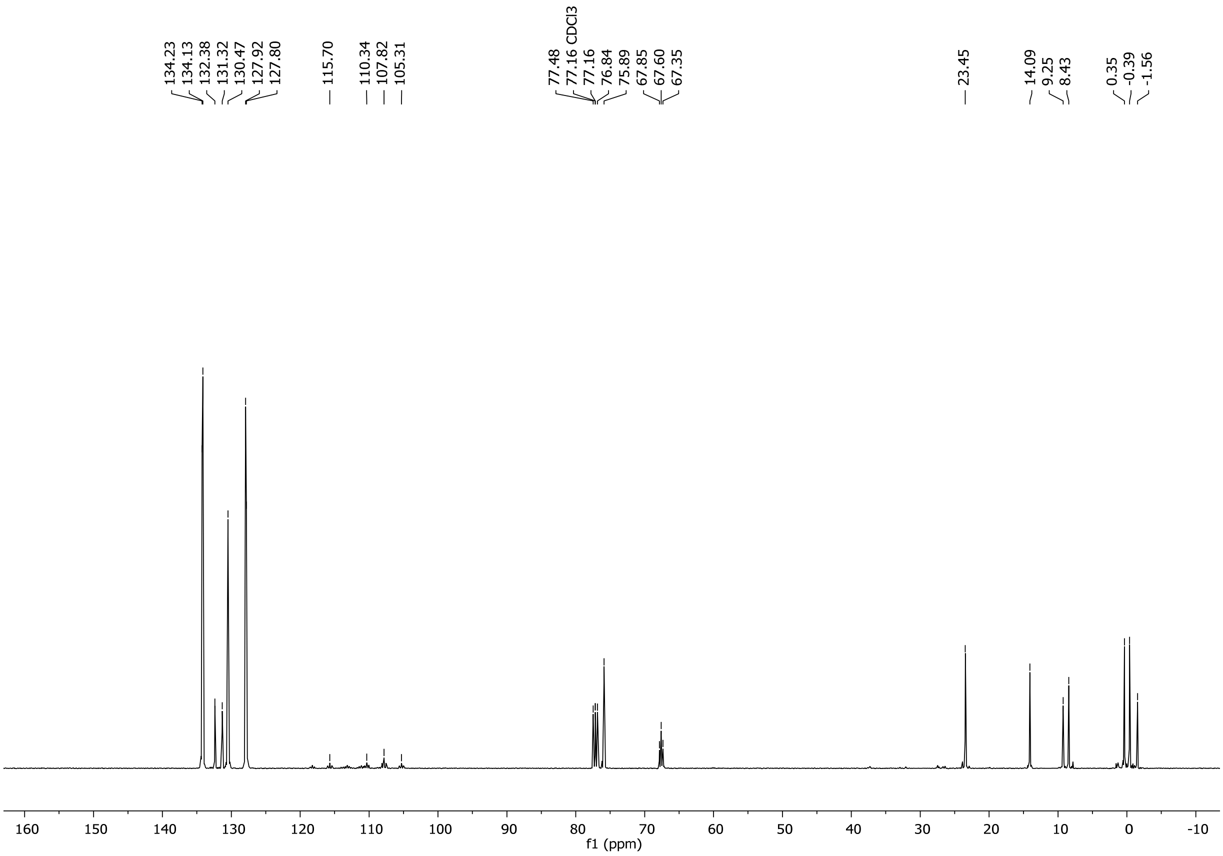

Figure S 23. ^{13}C NMR (*101 MHz*, $CDCl_3$) spectrum of **DDSQ-2Si(F3).**

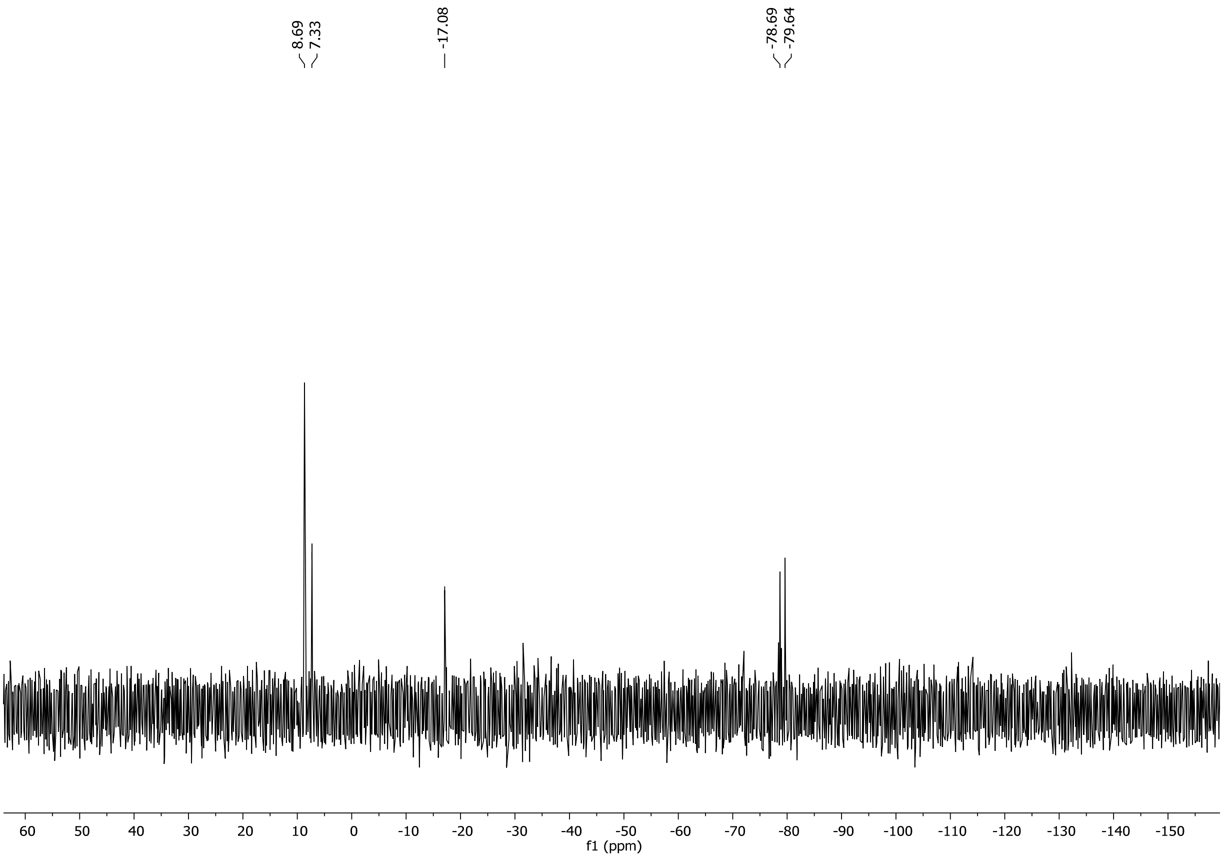

Figure S 24. ^{29}Si NMR (79.5 MHz, $CDCl_3$) spectrum of **DDSQ-2Si(F3).**

DDSQ-2Si(F4)

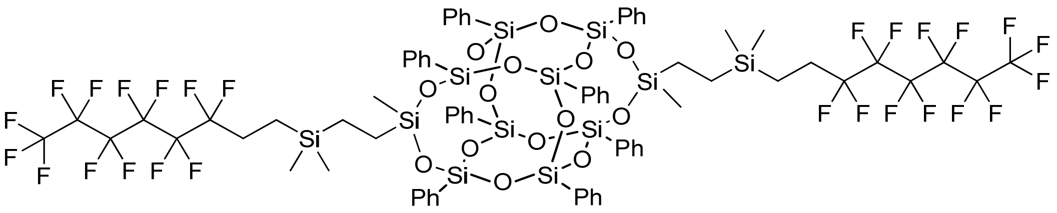

White solid. Isolated Yield 88%

^{1}H NMR (300 MHz, $CDCl_3$, ppm): δ = -0.16 (s, 12H, $Si(CH_3)_2$), 0.29 (s, 6H, $SiCH_3$,), 0.52-0.61 (m, 12H, Si-CH_2-, -CH_2-), 1.76-1.93 (m, 4H, -CH_2-), 7.20-7.54 (m, 40H, Ph);

^{13}C NMR (101 MHz, $CDCl_3$, ppm): δ = -4.46 (-$Si(CH_3)$), -1.63-($Si(CH_3)_2$), 3.84 (-CH_2-), 5.67 (-CH_2-), 8.66 (-CH_2-), 25.49-25.97 (-CH_2-), 115.82 (-CF-), 117.52 (-CF-), 118.11 (-CF-), 118.43 (-CF-),118.71 (-CF-), 119.59 (-CF-), 125.32 (Ph), 127.67-128.25 (Ph), 129.05 (Ph), 130.40 (Ph), 131.06 (Ph), 132.05 (Ph), 133.91-134.04 (Ph);

^{29}Si NMR (79 MHz, $CDCl_3$, ppm): δ = -5.59 (-Si-($CH_3)_2$-), -17.60 (-Si-(CH_3)-), -78.60, -79.57, -79.64, -79.72 (-Si-Ph);

IR (ATR, cm^{-1}): 3073.16, 3052.02, 3027.25 (C-H phenyl), 2954.81, 2909.70 (C-H), 1594.22, 1430.38 (C=C phenyl), 1239.96 (Si-C), 1207.93 (C-F), 1029.19 (Si-O-Si), 998.35 (C-H phenyl).

EA: Anal. calcd for $C_{74}H_{74}F_{26}O_{14}Si_{12}$ (%): C, 44.04; H, 3.70; found: C, 44.16; H, 3.71.

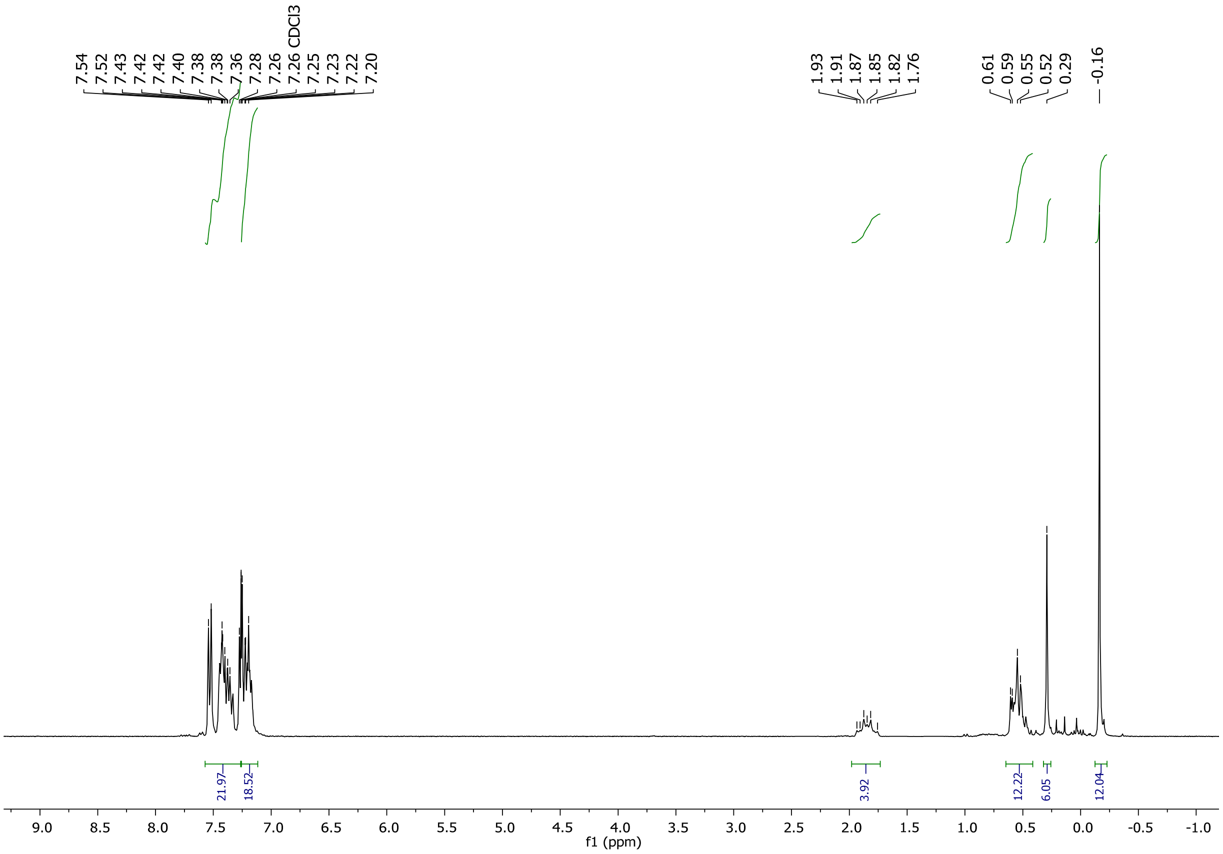

Figure S 25. ^{1}H NMR (300 MHz, $CDCl_3$) spectrum of **DDSQ-2Si(F4).**

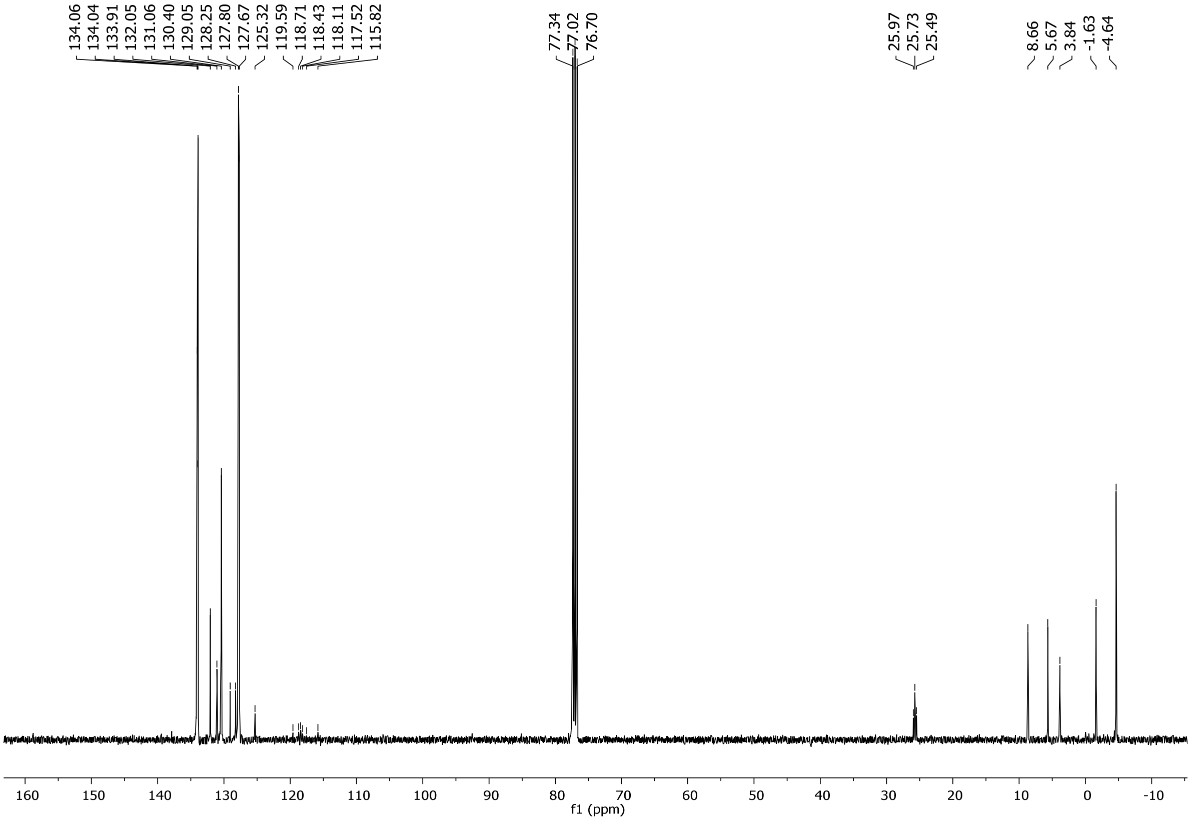

Figure S 26. ^{13}C NMR (*101 MHz*, $CDCl_3$) spectrum of **DDSQ-2Si(F4).**

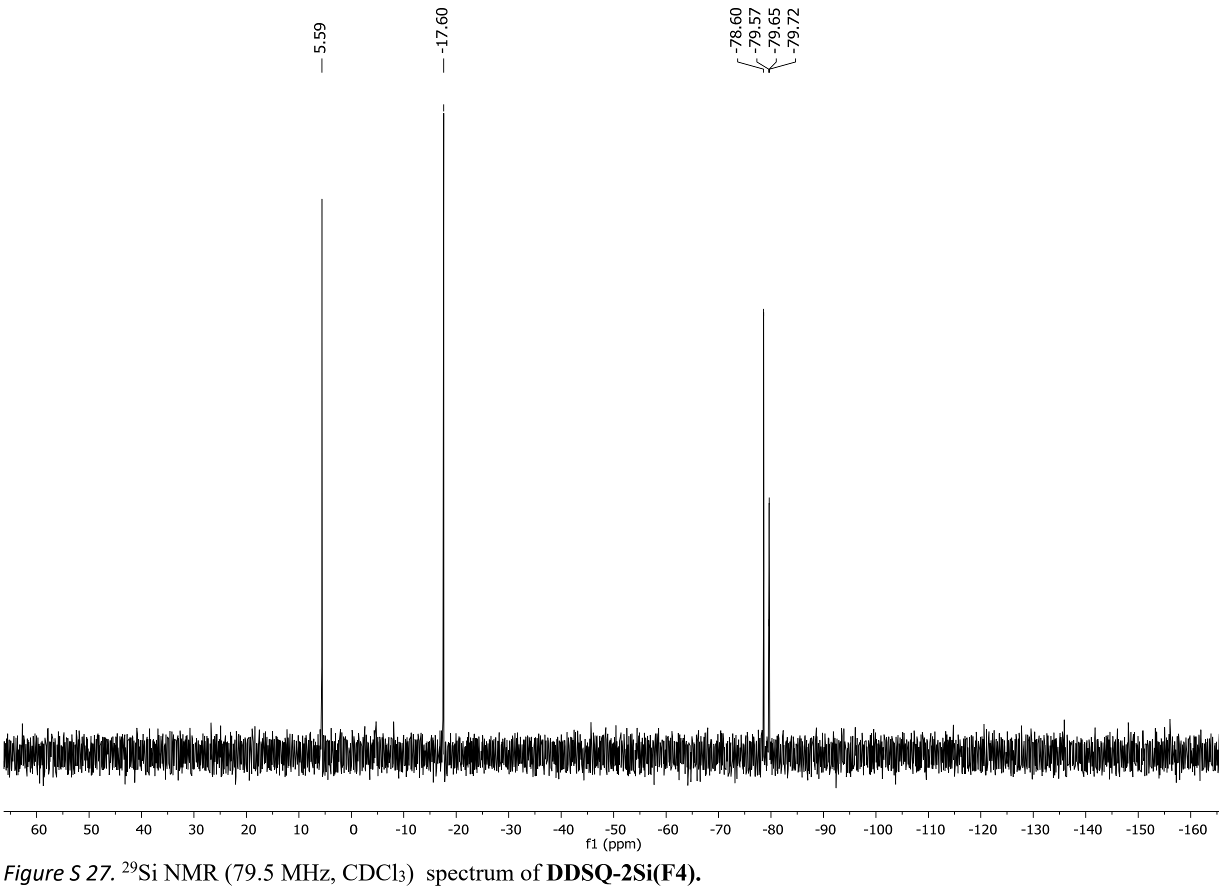

Figure S 27. ^{29}Si NMR (79.5 MHz, $CDCl_3$) spectrum of **DDSQ-2Si(F4).**

DDSQ-2Si(F5)

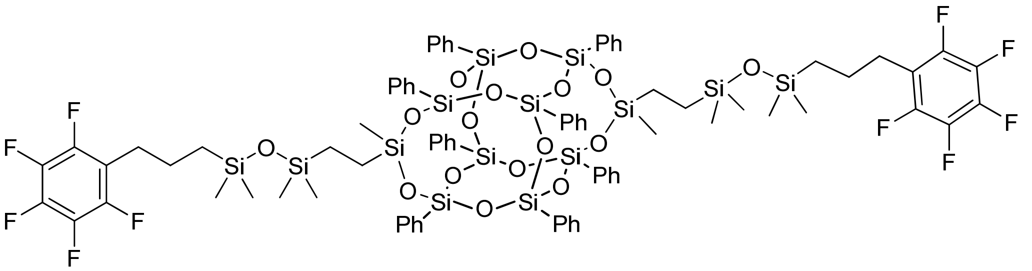

Waxy white solid. Isolated Yield 87%

^{1}H NMR (300 MHz, $CDCl_3$, ppm): δ = -0.09 (s, 12H, $Si(CH_3)_2$), -0.03 (s, 12H, $SiCH_3$,), 0.03-0.11 (m, 4H, Si-CH_2-), 0.32 (s, 6H, $Si(CH_3)$), 0.46-0.54 (m, 4H, -CH_2-), 0.59-0.65 (m, 4H, -CH_2-), 1.49-1.60 (m, 4H, -CH_2-), 2.63 (t, $J_{H\text{-}H}$ = 7.7 Hz, 4H,-CH_2-), 7.20-7.58 (m, 40H, Ph);

^{13}C NMR (101 MHz, $CDCl_3$, ppm): δ = -1.59 (-$Si(CH_3)$), 0.41-($Si(CH_3)_2$), 0.37 (-$Si(CH_3)_2$), 8.37 (-Si-CH_2-), 9.20 (-CH_2-Si), 18.20 (-Si-CH_2-), 23.54 (-CH_2-), 25.86 (-CH_2-), 115.47 (-C-), 127.73-127.88 (Ph), 130.43 (Ph), 131.28 (Ph), 132.34 (Ph), 134.09-134.20 (Ph), 136.24 (-CF-), 138.72 (-CF-), 140.74 (-CF-), 143.93 (-CF-), 146.37 (-CF-);

^{29}Si NMR (79 MHz, $CDCl_3$, ppm): δ = 8.77, 6.80 (-Si-(CH_3)$_2$-), -17.08 (-Si-(CH_3)-), -78.67, -79.58, -79.63, -79.67 (-Si-Ph);

IR (ATR, cm^{-1}): 3073.02, 3051.29, 3027.75 (C-H phenyl), 2956.08, 2920.51 (C-H), 1655.03, 1594.11 (C=C phenyl), 1253.73 (Si-C), 1084.53.82, 1028.21.36 (Si-O-Si), 997.91 (C-H phenyl);

EA: Anal. calcd for $C_{80}H_{90}F_{10}O_{16}Si_{14}$ (%): C, 50.82; H, 4.80; found: C, 50.80; H, 4.79.

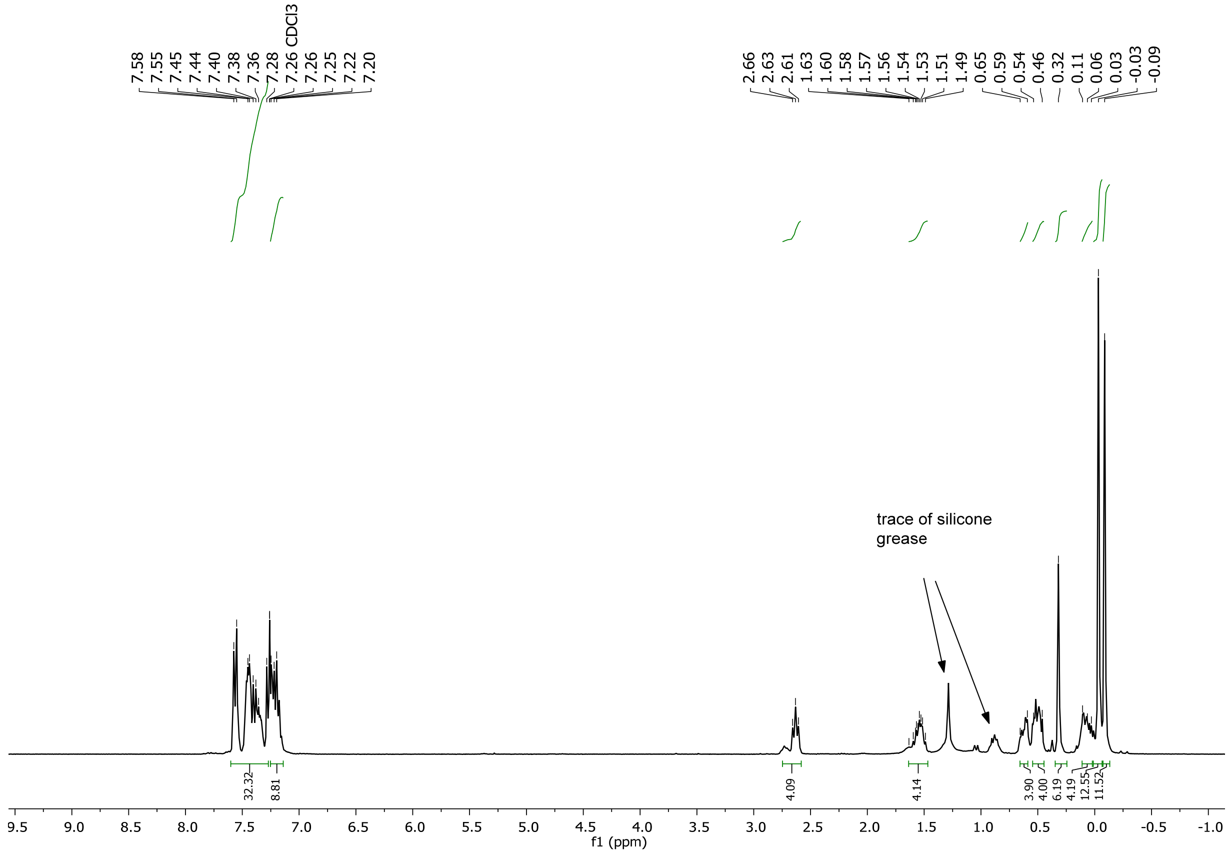

Figure S 28. ^{1}H NMR (300 MHz, $CDCl_3$) spectrum of **DDSQ-2Si(F5).**

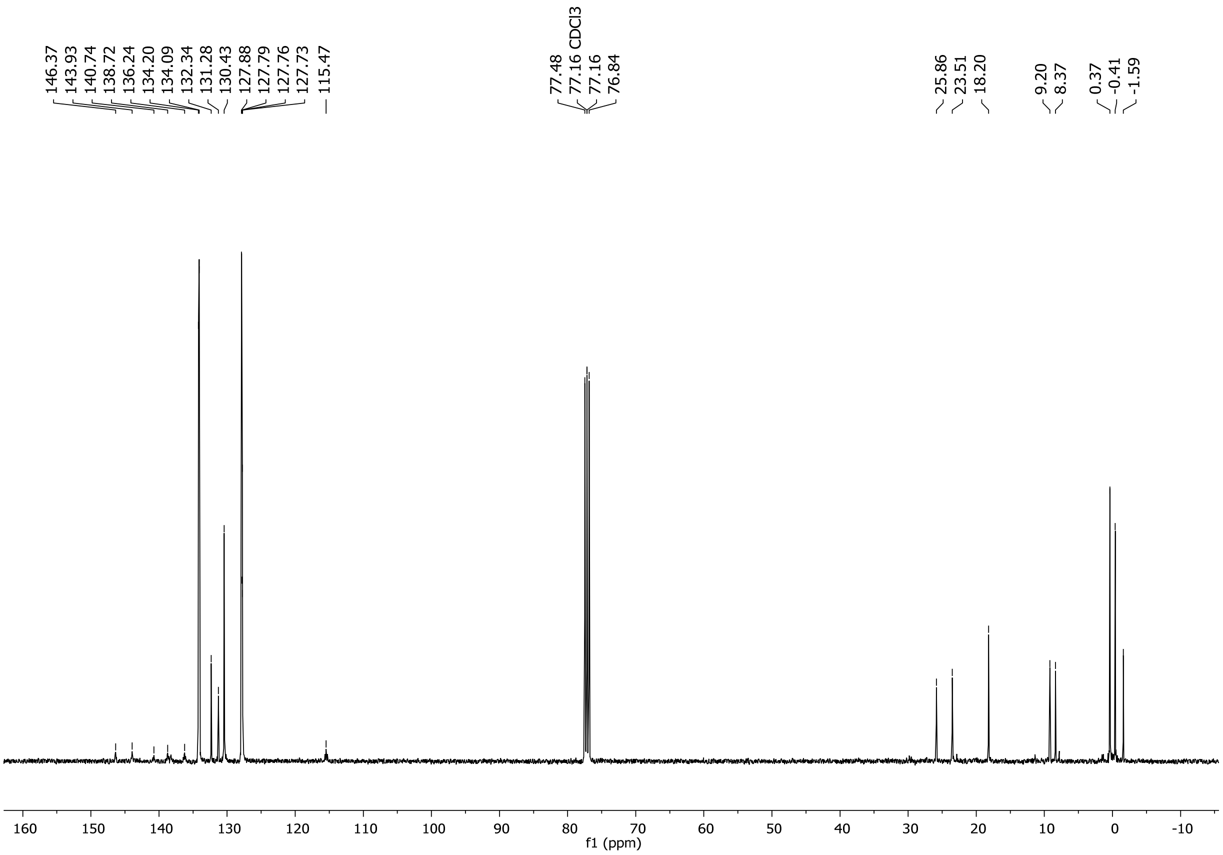

Figure S 29. ^{13}C NMR (*101 MHz*, $CDCl_3$) spectrum of **DDSQ-2Si(F5).**

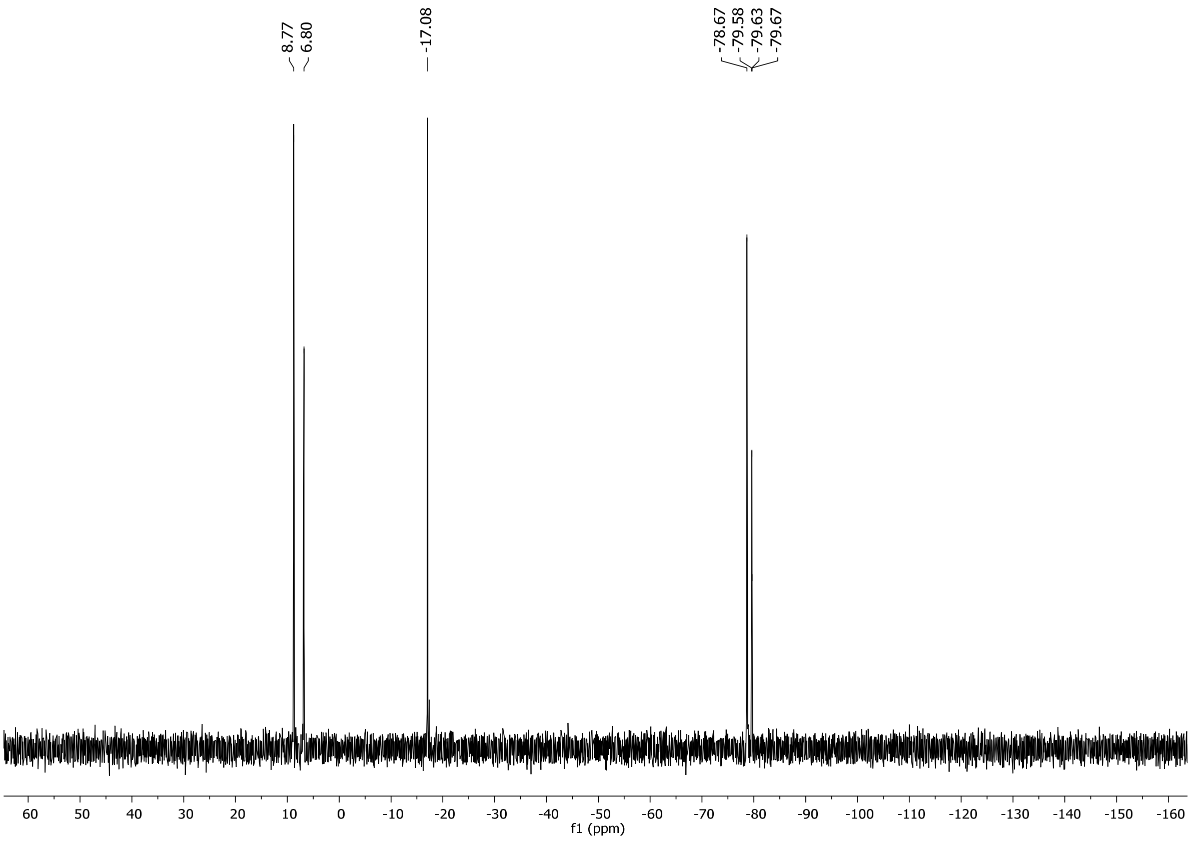

Figure S 30. ^{29}Si NMR (79.5 MHz, $CDCl_3$) spectrum of **DDSQ-2Si(F5).**

DDSQ-2OSi(F3)

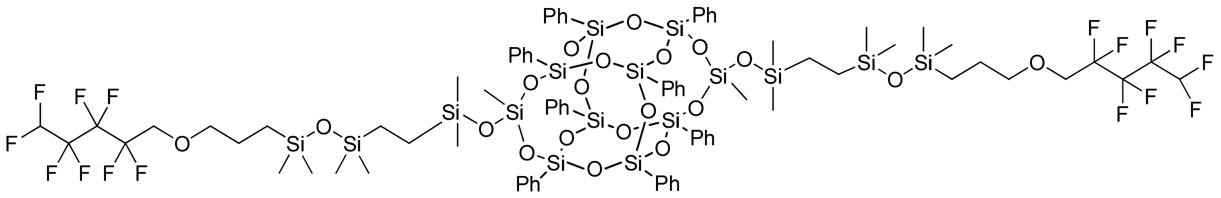

White solid. Isolated Yield 94%

[1]**H NMR** (300 MHz, $CDCl_3$, ppm): δ = -0.08 (s, 12H, $Si(CH_3)_2$), 0.00 (s, 12H, $Si(CH_3)_2$, 0.08 (s, 12H, Si-CH_2-), 0.28 (s, 6H, Si-CH_2-), 0.37-0.40 (d, J_{H-H} = 5.5 Hz, 4H, $Si(CH_3)_2$), 0.43-0.49 (m, 4H, -CH_2-Si-),0.84-0.97 (td, J_{H-H} = 15.4, 7.7 Hz, 4H, Si-CH_2-), 1.51-1.60 (m, 4H, -CH_2-), 3.50 (t, J_{H-H} = 6.9 Hz, 4H, -CH_2-O), 3.88 (t, J_{H-H} = 14.1 Hz, 4H,-O-CH_2-), 6.05 (tt, J_{H-H} = 52.1, 5.7 Hz, 2H,-CH-F_2), 7.16-7.58 (m, 40H, Ph);

[13]**C NMR** (101 MHz, $CDCl_3$, ppm): δ = -2.59 (-Si(CH_3)), -0.61-($Si(CH_3)_2$), -0.36 (-$Si(CH_3)_2$), 0.38 (-$Si(CH_3)_2$), 9.40 (-Si-CH_2-), 9.53 (-CH_2-Si), 14.09 (-Si-CH_2-), 23.46 (-CH_2-), 67.46-67.86 (-CH_2-O-), 75.93 (O-CH_2-), 105.27 (-CF_2-), 107.79 (-CF_2-), 110.31 (-CHF_2-), 115.67 (-CF_2-), 127.63-127.94 (Ph), 130.44-130.49 (Ph), 131.19 (Ph), 132.11 (Ph), 134.10-134.30 (Ph);

[29]**Si NMR** (79 MHz, $CDCl_3$, ppm): δ = 10.88 (-Si-($CH_3)_2$-), 8.80 (-Si-($CH_3)_2$-), 7.20 (-Si-($CH_3)_2$), -64.23 (-$SiCH_3$), -78.25, -79.31, -79.52 (-Si-Ph);

IR (ATR, cm^{-1}): 3073.64, 3052.10 (C-H phenyl), 2956.20, 2922.43 (C-H), 1594.58, 1430.51 (C=C phenyl), 1253.28 (Si-C), 1170.38 (C-F), 1107.03, 1028.21 (Si-O-Si), 997.84 (C-H phenyl).

EA: Anal. calcd for $C_{82}H_{108}F_{16}O_{20}Si_{16}$ (%): C, 45.45; H, 5.02; found: C, 45.38; H, 5.01.

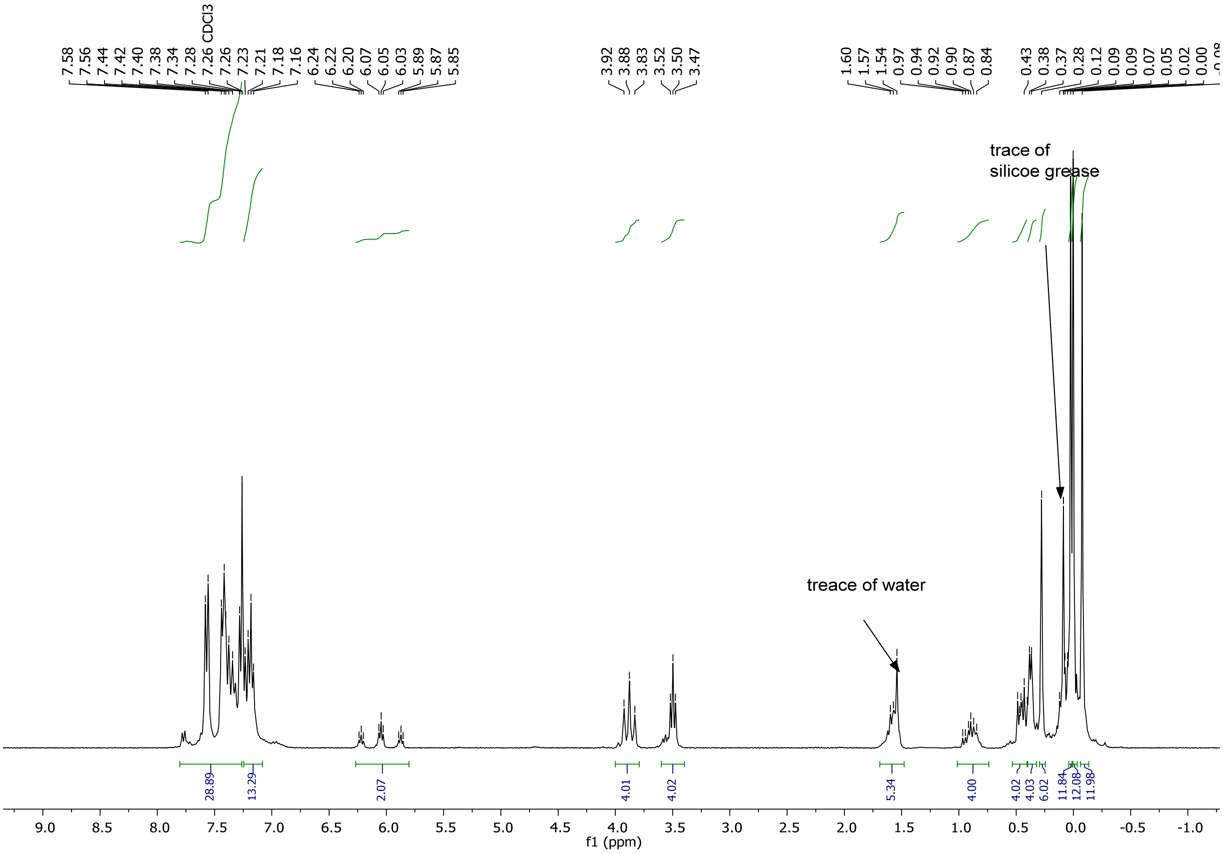

Figure S 31. ^{1}H NMR (300 MHz, $CDCl_3$) spectrum of **DDSQ-2OSi(F3).**

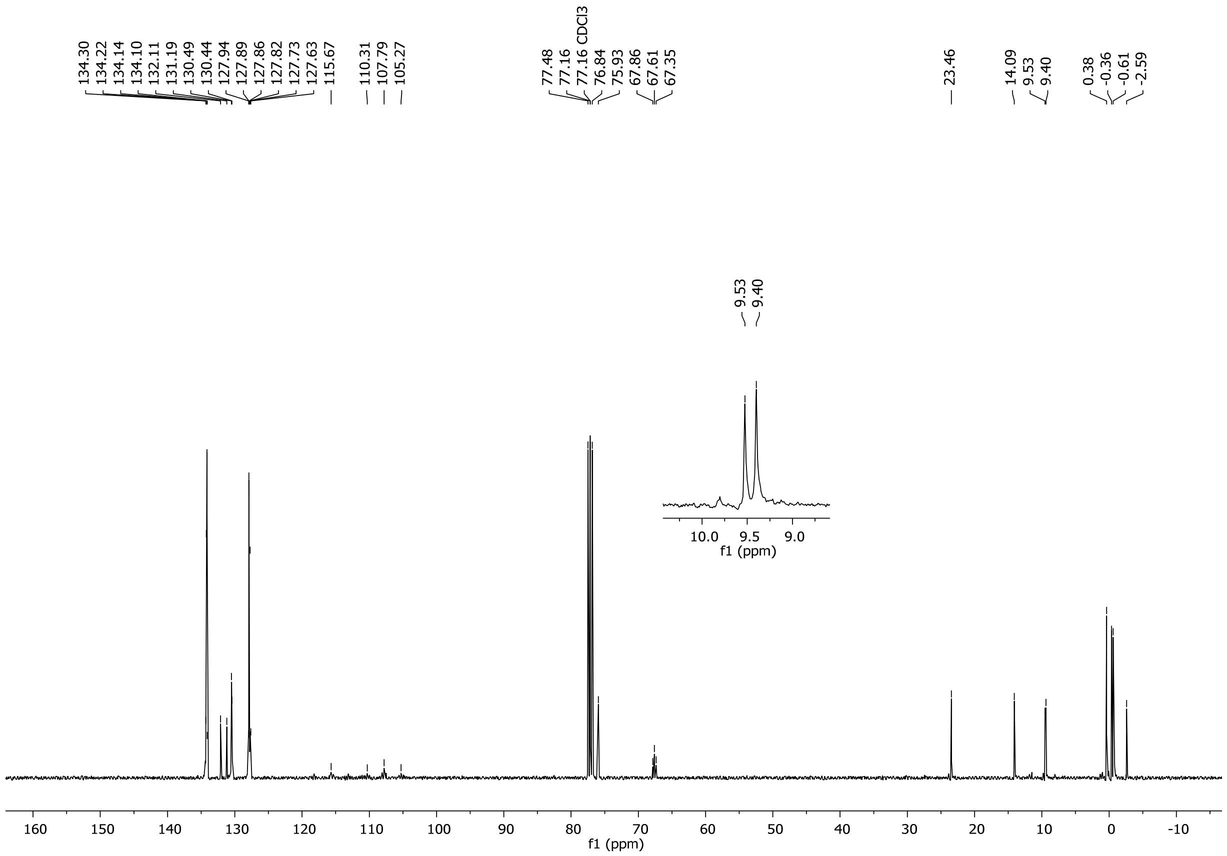

Figure S 32. ^{13}C NMR (*101 MHz*, $CDCl_3$) spectrum of **DDSQ-2OSi(F3).**

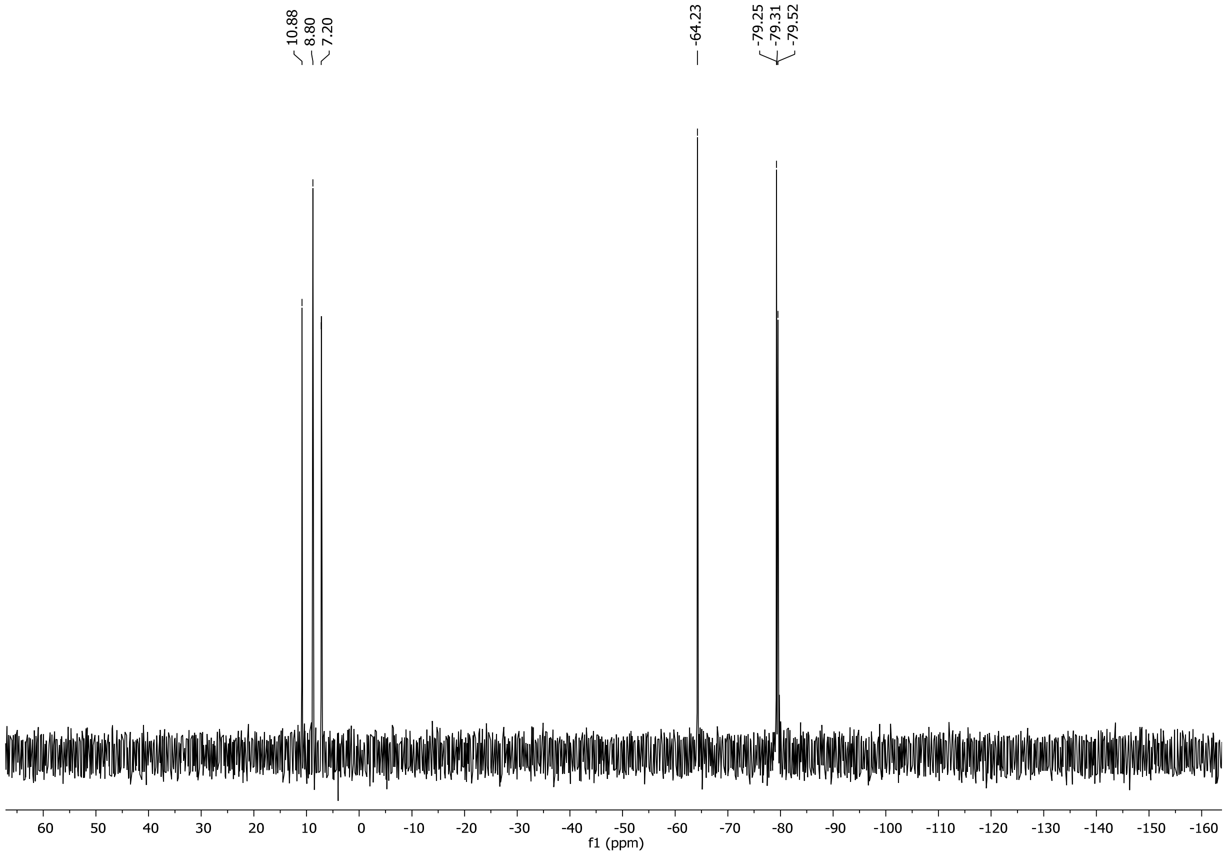

Figure S 33. ^{29}Si NMR (79.5 MHz, $CDCl_3$) spectrum of **DDSQ-2OSi(F3).**

triT$_7$(F3)

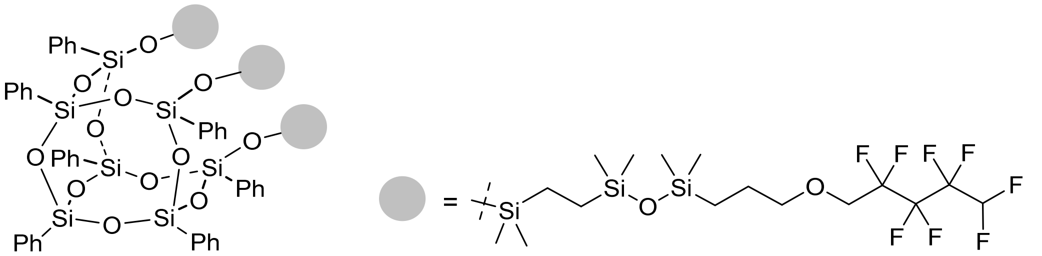

Oil. Isolated Yield 90%

^{1}H NMR (300 MHz, CDCl$_3$, ppm): δ = -0.03 (s, 18H, Si(CH$_3$)$_2$), 0.02 (s, 18H, Si(CH$_3$)$_2$, 0.15-0.13 (m, 6H, Si-CH$_2$-), 0.27 (s, 18H, Si(CH$_3$)$_2$), 0.35-0.48 (m, 6H, -CH$_2$-Si-), 0.50-0.60 (m, 6H, Si-CH$_2$-), 1.56-1.67 (m, 6H, -CH$_2$-), 3.53 (dt, $J_{H\text{-}H}$ = 14.0, 6.6 Hz, 6H, -CH$_2$-O), 3.91 (q, $J_{H\text{-}H}$ = 13.9 Hz, 6H,-O-CH$_2$-), 6.07 (tq, $J_{H\text{-}H}$ = 52.00, 5.0 Hz, 3H,-CH-F$_2$), 7.09-7.57 (m, 35H, Ph);

^{13}C NMR (101 MHz, CDCl$_3$, ppm): δ = -0.36 (-Si(CH$_3$)$_2$), -0.26 (Si(CH$_3$)$_2$), 0.37 (-Si(CH$_3$)$_2$), 9.48 (-Si-CH$_2$-), 9.58 (-CH$_2$-Si), 14.06 (-Si-CH$_2$-), 23.45 (-CH$_2$-), 67.26-67.90 (-CH$_2$-O-), 75.93 (O-CH$_2$-), 105.98 (-CF$_2$-), 107.76 (-CF$_2$-), 111.09 (-CHF$_2$-), 115.66 (-CF$_2$-), 127.61-127.99 (Ph), 130.06-130.22 (Ph), 130.74 (Ph), 131.34, 133.09 (Ph), 139.07-134.14 (Ph);

^{29}Si NMR (79 MHz, CDCl$_3$, ppm): δ = 12.49 (-Si-(CH$_3$)$_2$-), 8.76 (-Si-(CH$_3$)$_2$-), 7.17 (-Si-(CH$_3$)$_2$-), -77.42, -77.91, -78.19 (-Si-Ph).

IR (ATR, cm^{-1}): 3074.01, 3052.40 (C-H phenyl), 2956.92, 2879.77 (C-H), 1594.50, 1430.69 (C=C phenyl), 1253.78 (Si-C), 1168.90 (C-F), 1128.82, 1041.36 (Si-O-Si), 998.11 (C-H phenyl).

EA: Anal. calcd for $C_{90}H_{128}F_{24}O_{18}Si_{16}$ (%): C, 44.98; H, 5.37; found: C, 45.11; H, 5.38.

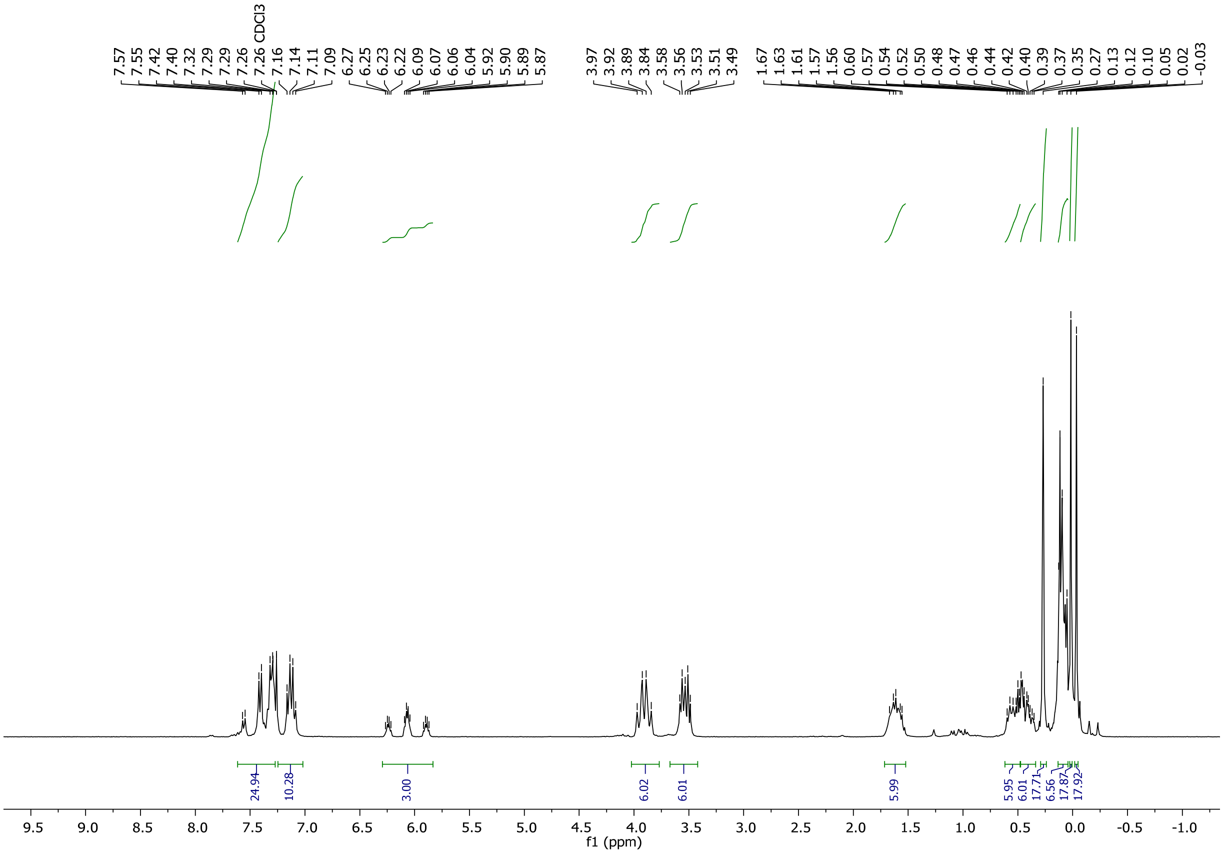

Figure S 34. ^{1}H NMR (300 MHz, CDCl$_3$) spectrum of **triT$_7$(F3).**

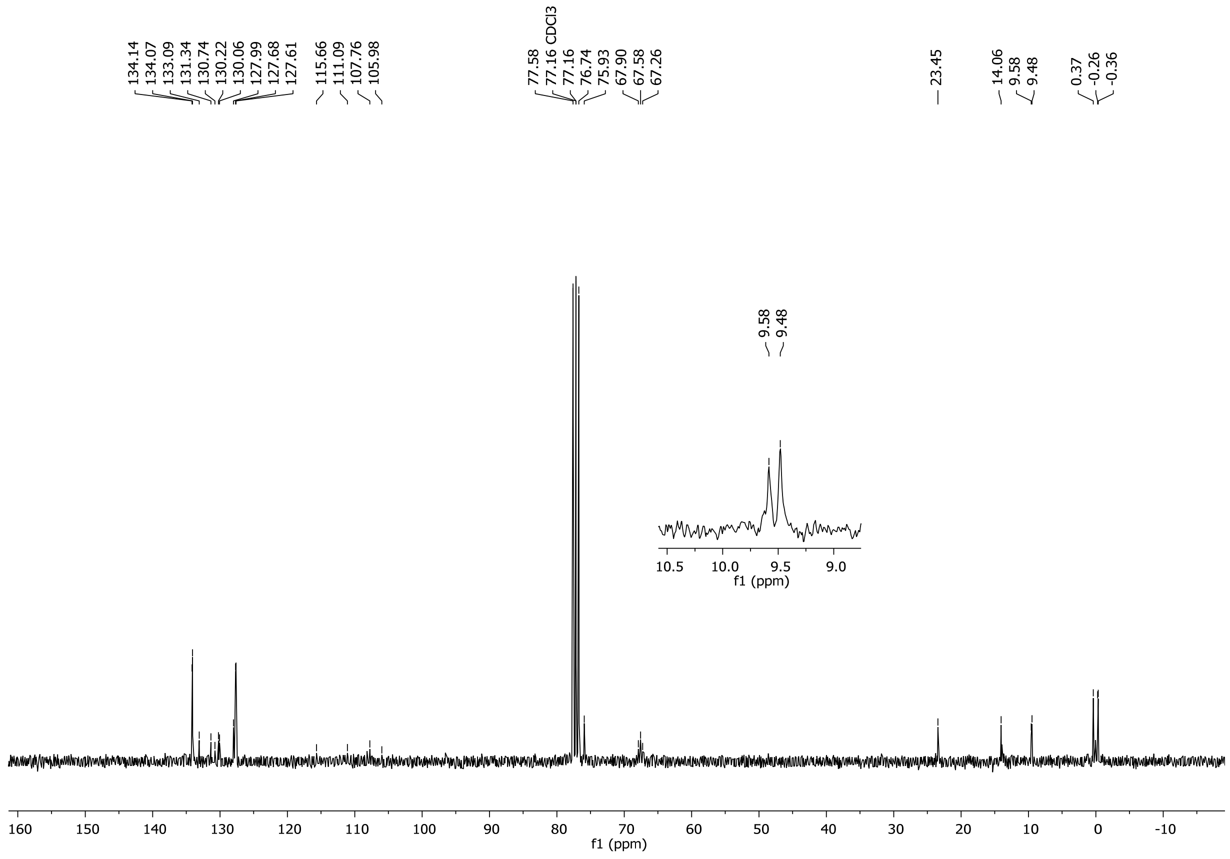

Figure S 35. ^{13}C NMR (*101 MHz*, $CDCl_3$) spectrum of **triT$_7$(F3).**

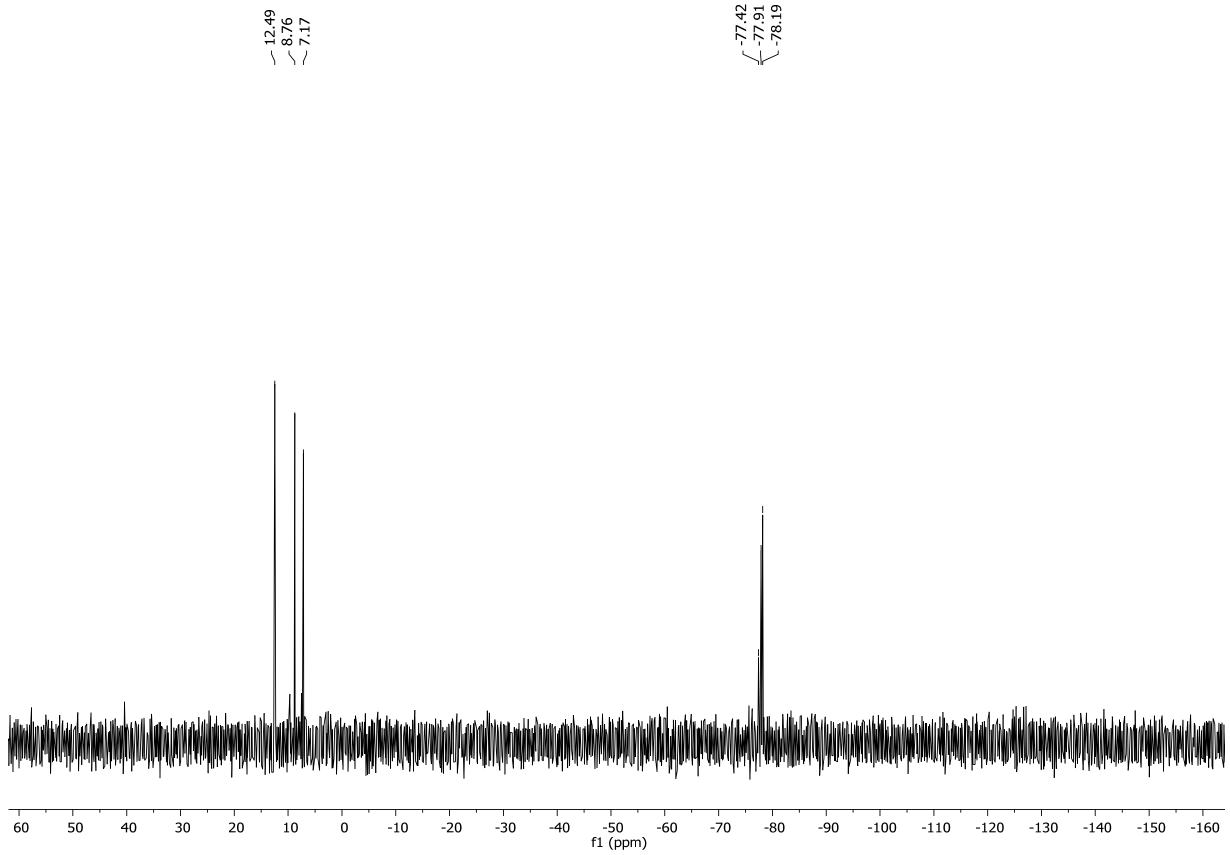

Figure S 36. ^{29}Si NMR (79.5 MHz, $CDCl_3$) spectrum of **triT$_7$(F3).**

DDSQ-4Si(F3)

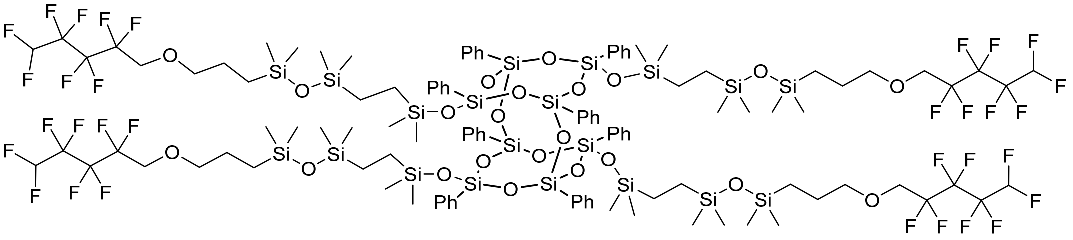

Waxy, white solid. Isolated Yield 94%

^{1}H NMR (400 MHz, $CDCl_3$, ppm): δ = -0.22 (d, $J_{H\text{-}H}$ = 1.8 Hz, 24H, $Si(CH_3)_2$), -0.05 (d, $J_{H\text{-}H}$ = 1.6 Hz, 24H, $Si(CH_3)_2$, 0.04 (d, $J_{H\text{-}H}$ = 1.8 Hz,, 24H, $Si(CH_3)_2$), 0.09-0.13 (m, 8H, Si-CH_2-), 0.27-0.31 (m, 8H, -CH_2-Si-), 0.39-0.43 (m, 8H, Si-CH_2-), 1.50-1.69 (m, 8H, -CH_2-), 3.49-3.58 (m, 8H, -CH_2-O), 3.88 (t, $J_{H\text{-}H}$ = 14.7 Hz, 8H,-O-CH_2-), 6.06 (tt, $J_{H\text{-}H}$ = 52.10, 5.6 Hz, 4H,-CH-F_2), 7.13-7.47 (m, 40H, Ph);
^{13}C NMR (101 MHz, $CDCl_3$, ppm): δ = -0.62 (-$Si(CH_3)_2$), -0.48 ($Si(CH_3)_2$), 0.30 (-$Si(CH_3)_2$), 9.55 (-Si-CH_2-), 9.58 (-CH_2-Si), 14.05 (-Si-CH_2-), 23.44 (-CH_2-), 67.44-67.77 (-CH_2-O-), 75.94 (O-CH_2-), 106.13 (-CF_2-), 107.82 (-CF_2-), 111.09 (-CHF_2-), 115.69 (-CF_2-), 127.59-127.62 (Ph), 129.89 (Ph), 130.25 (Ph), 131.95 (Ph), 133.28 (Ph), 134.28-134.50 (Ph);
^{29}Si NMR (79 MHz, $CDCl_3$, ppm): δ = 6.96 (-Si-$(Me)_2$-), 8.71 (-Si-$(Me)_2$-), 11.76 (-Si-$(Me)_2$-), -76.10, -78.67 (-Si-Ph);
IR (ATR, cm^{-1}): 3074.38, 3054.72 (C-H phenyl), 2957.00, 2924.57 (C-H), 1594.42, 1430.50 (C=C phenyl), 1252.59 (Si-C), 1170.08 (C-F), 1128.29, 1041.47 (Si-O-Si), 998.47 (C-H phenyl).
The spectroscopic data are consistent with the literature.[64]

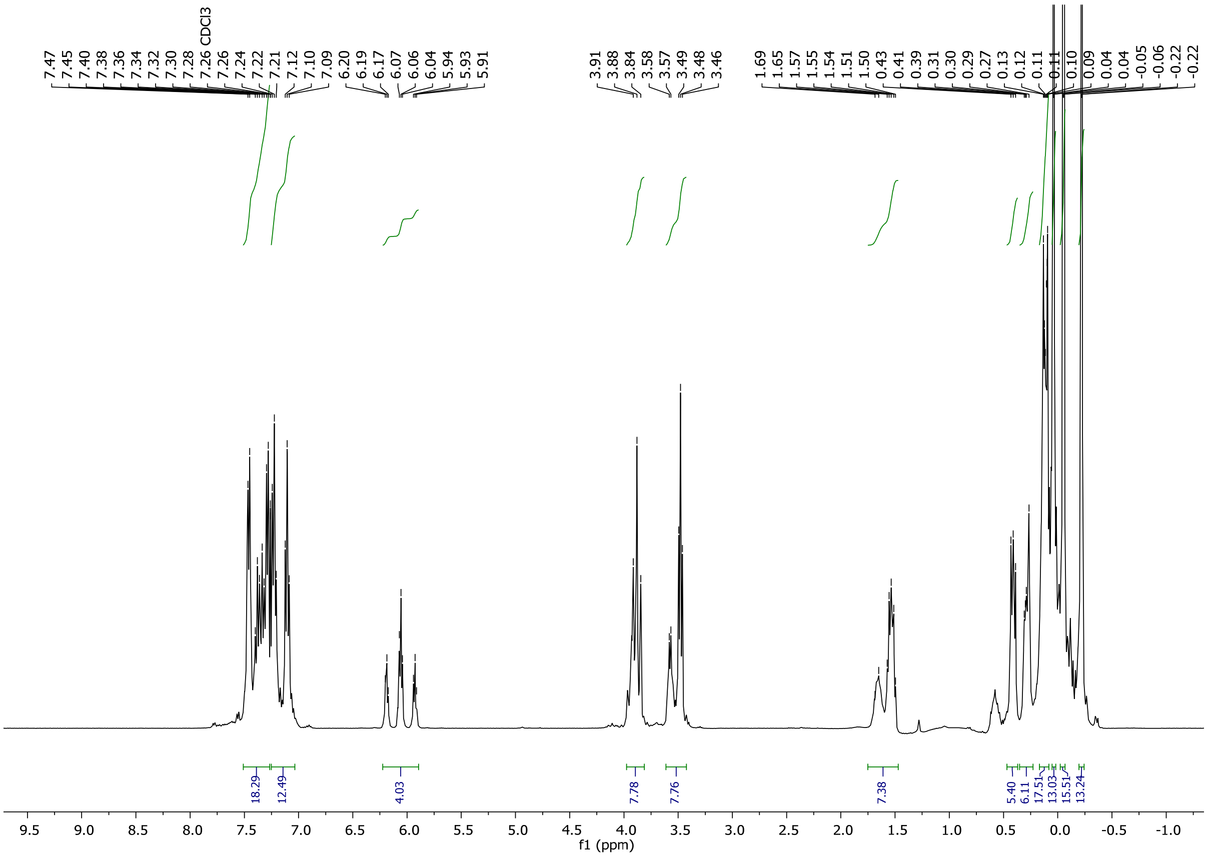

Figure S 37. ^{1}H NMR (300 MHz, $CDCl_3$) spectrum of **DDSQ-4Si(F3).**

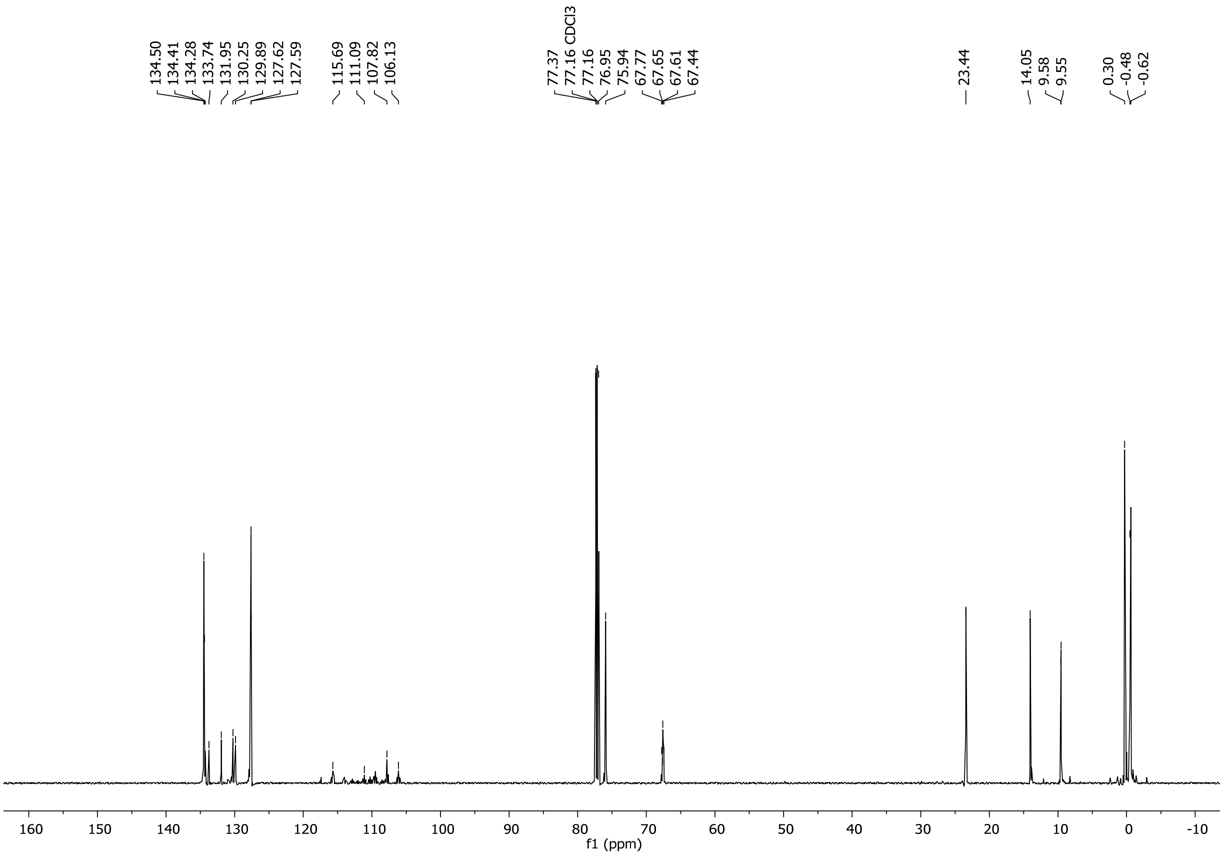

Figure S 38. ^{13}C NMR (*75.5 MHz*, $CDCl_3$) spectrum of **DDSQ-4Si(F3).**

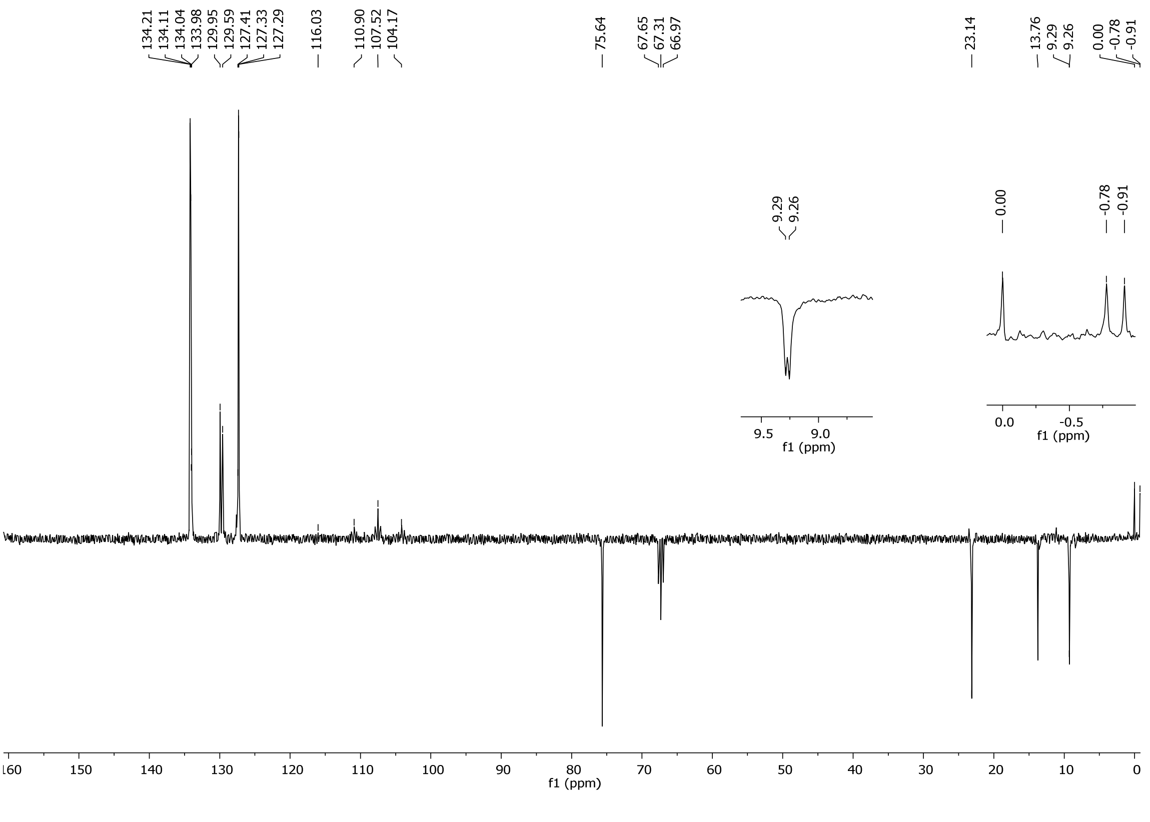

Figure S 39. ^{13}C DEPT 135 NMR (*75.5 MHz*, $CDCl_3$) spectrum of **DDSQ-4Si(F3).**

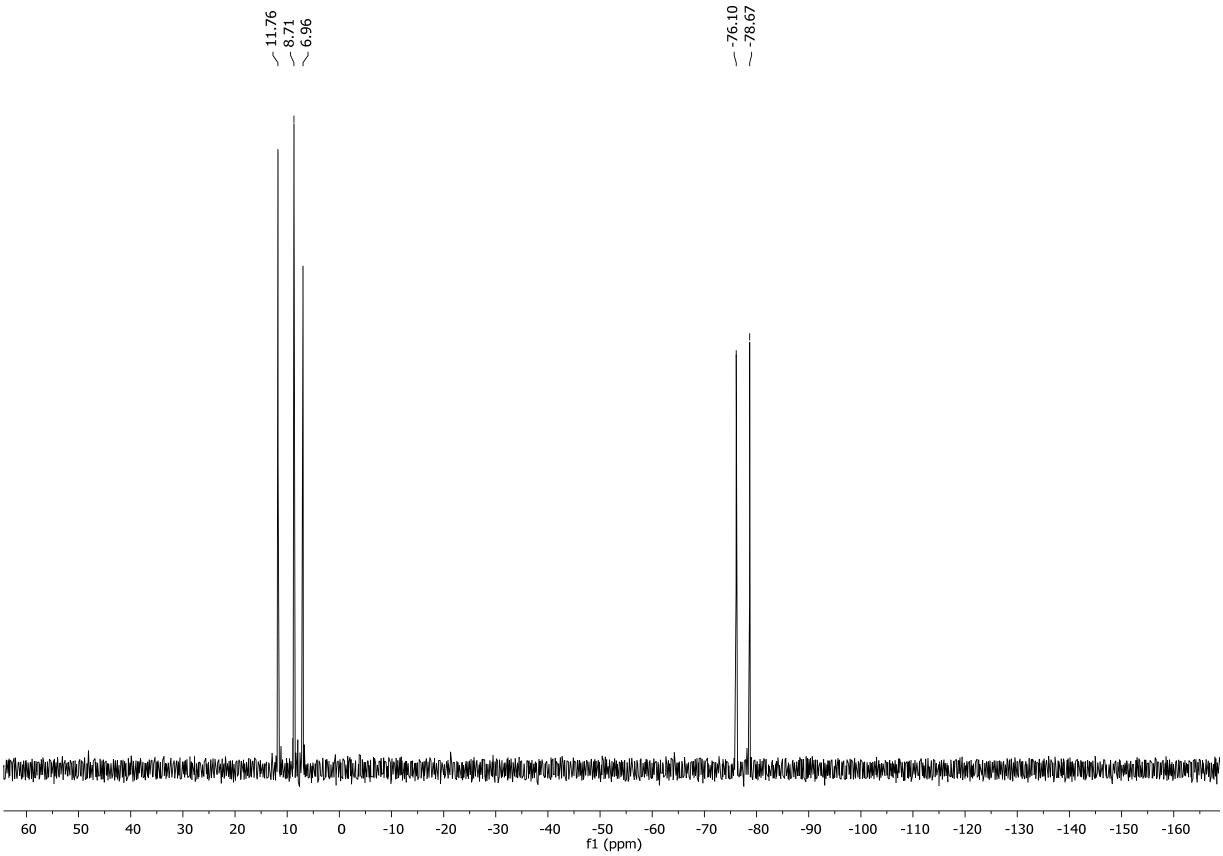

Figure S 40. ^{29}Si NMR (79.5 MHz, $CDCl_3$) spectrum of **DDSQ-4Si(F3).**

octaT$_8$(F3)

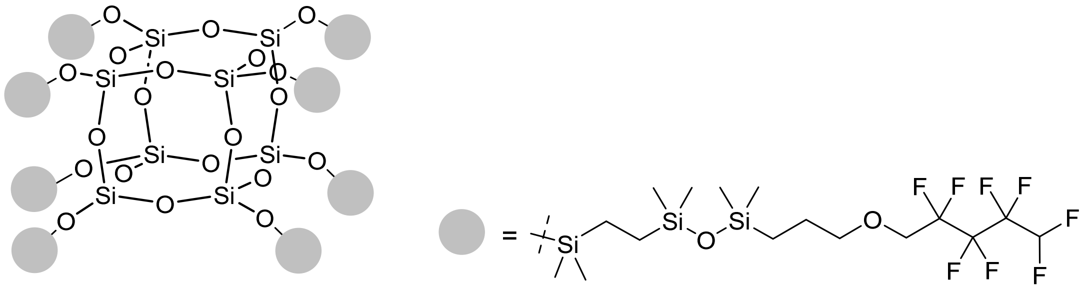

Oil. Isolated Yield 92%

^{1}H NMR (400 MHz, $CDCl_3$, ppm): δ = 0.03 (s, 48H, $Si(CH_3)_2$), 0.05 (s, 48H, $Si(CH_3)_2$, 0.08-0.10 (m, 16H, Si-CH_2-), 0.12 (s, 48H, $Si(CH_3)_2$), 0.41-0.45 (m, 16H, -CH_2-Si-), 0.47-0.53 (m, 16H, Si-CH_2-), 1.56-1.66 (m, 16H, -CH_2-), 3.54 (t, J_{H-H} = 6.9 Hz, 16H, -CH_2-O), 3.91 (t, J_{H-H} = 14.0 Hz, 16H,-O-CH_2-), 6.06 (tt, J_{H-H} = 52.00, 5.6 Hz, 8H,-CH-F_2);

^{13}C NMR (101 MHz, $CDCl_3$, ppm): δ = -0.96 (-$Si(CH_3)_2$), -0.28 ($Si(CH_3)_2$), 0.37(-$Si(CH_3)_2$), 9.07 (-Si-CH_2-), 9.36 (-CH_2-Si), 14.07 (-Si-CH_2-), 23.46 (-CH_2-), 67.24-67.90 (-CH_2-O-), 75.94 (O-CH_2-), 107.73 (-CF_2-), 111.11 (-CF_2-), 116.04 (-CHF_2-), 119.05 (-CF_2-);

^{29}Si NMR (79 MHz, $CDCl_3$, ppm): δ = 7.26 (-Si-($CH_3)_2$-), 8.70 (-Si-($CH_3)_2$-), 13.24 (-Si-($CH_3)_2$-), -108.79 (-Si-);

IR (ATR, cm^{-1}): 2956.83, 2910.07 (C-H), 1252.75 (Si-C), 1167.60 (C-F), 1131.30, 1045.49 (Si-O-Si).

EA: Anal. calcd for C128H248F64O36Si32 (%): C, 34.33; H, 5.58; found: C, 34.43; H, 5.59.

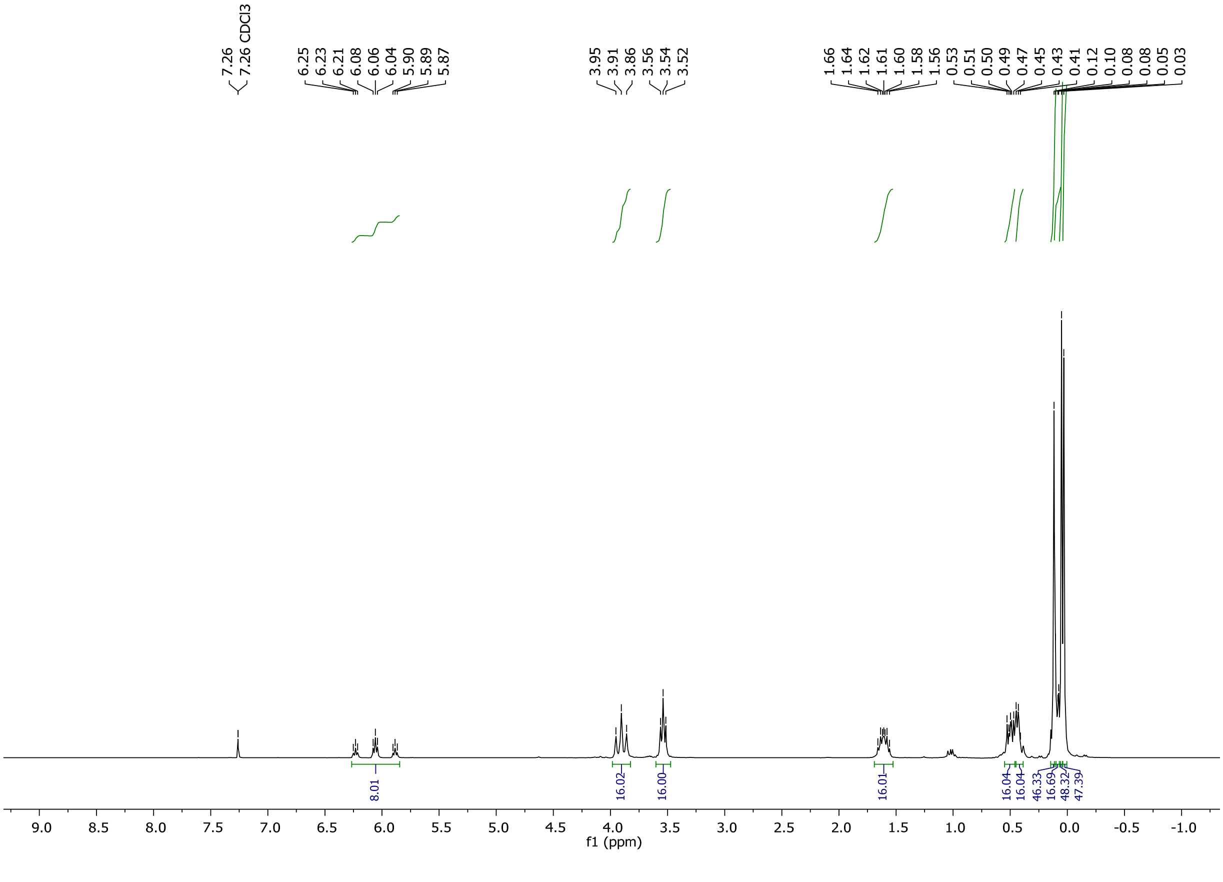

Figure S 41. ^{1}H NMR (300 MHz, $CDCl_3$) spectrum of **octaT$_8$(F3).**

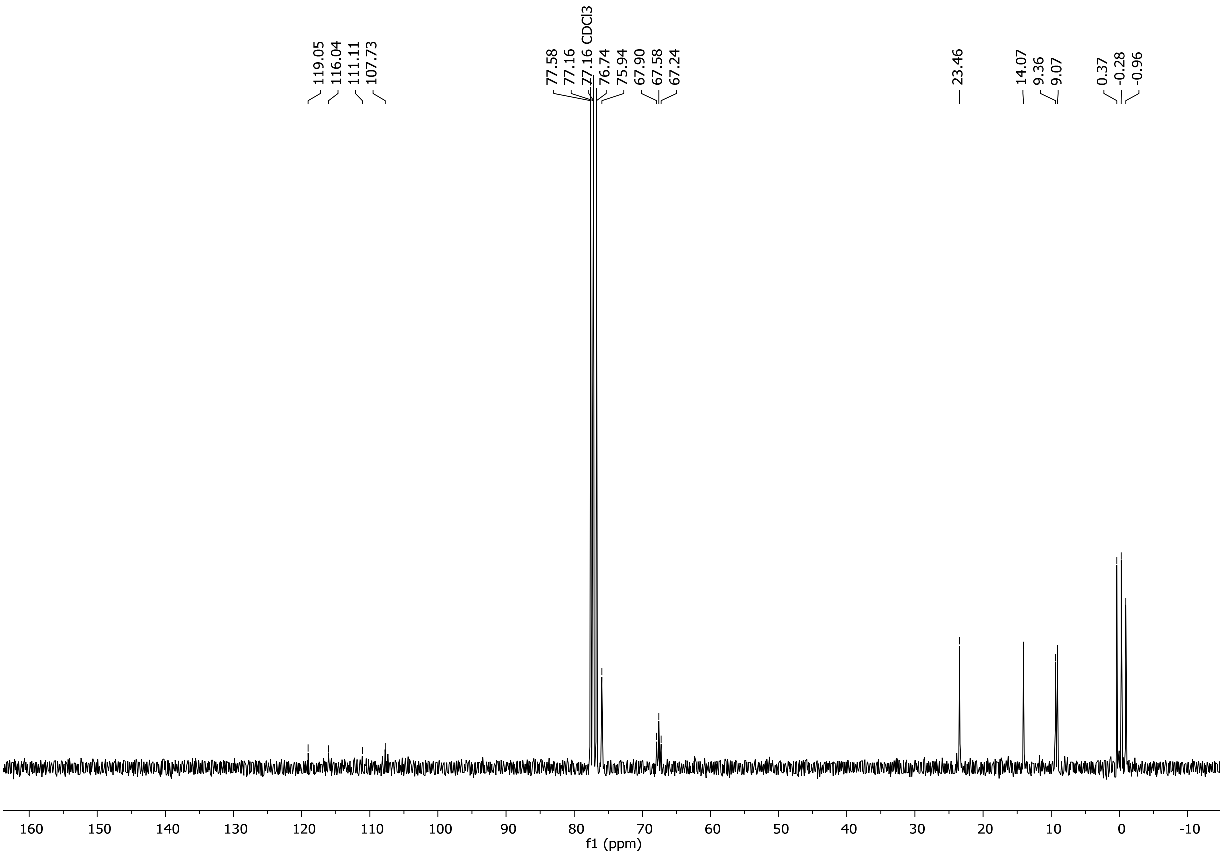

Figure S 42. ^{13}C NMR (*75.5 MHz*, $CDCl_3$) spectrum of **octaT$_8$(F3).**

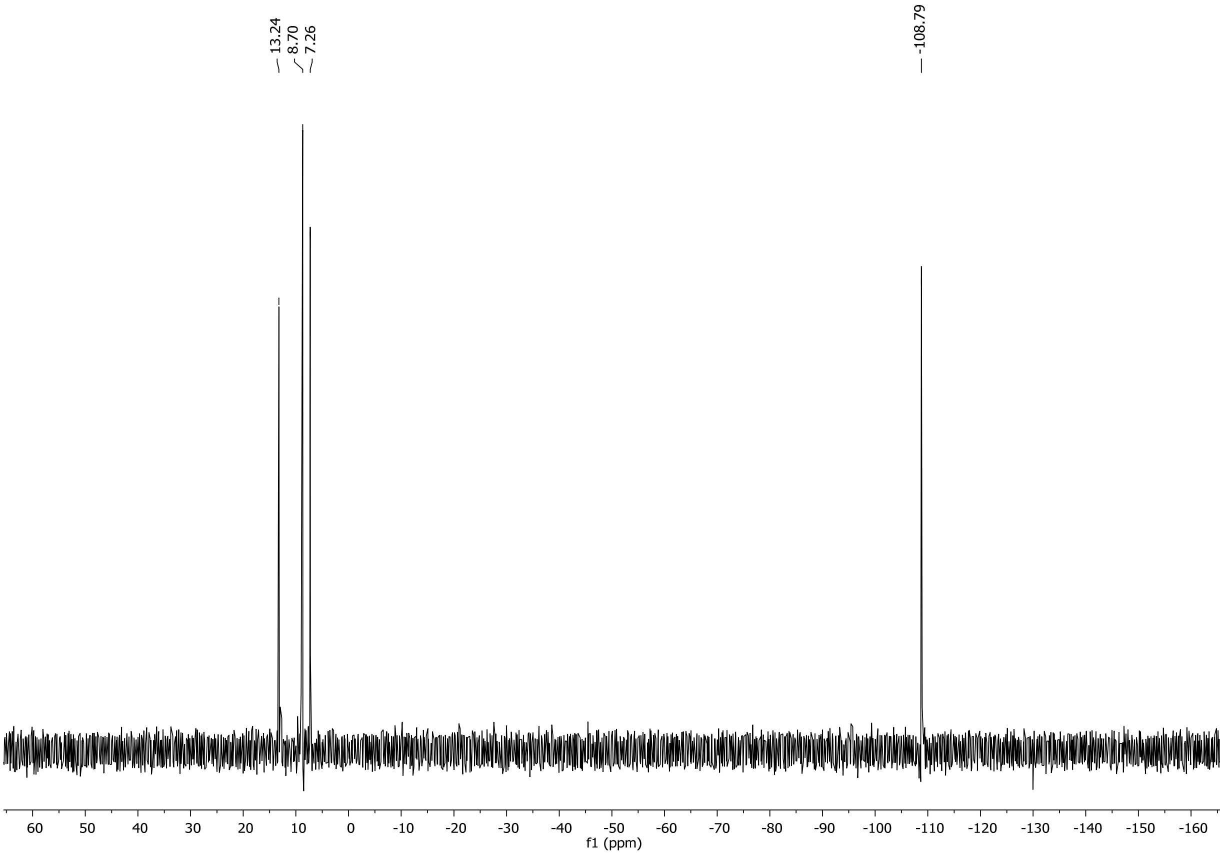

Figure S 43. ^{29}Si NMR (79.5 MHz, $CDCl_3$) spectrum of **octaT$_8$(F3).**

3. Additional ^{29}Si NMR spectra comparisons

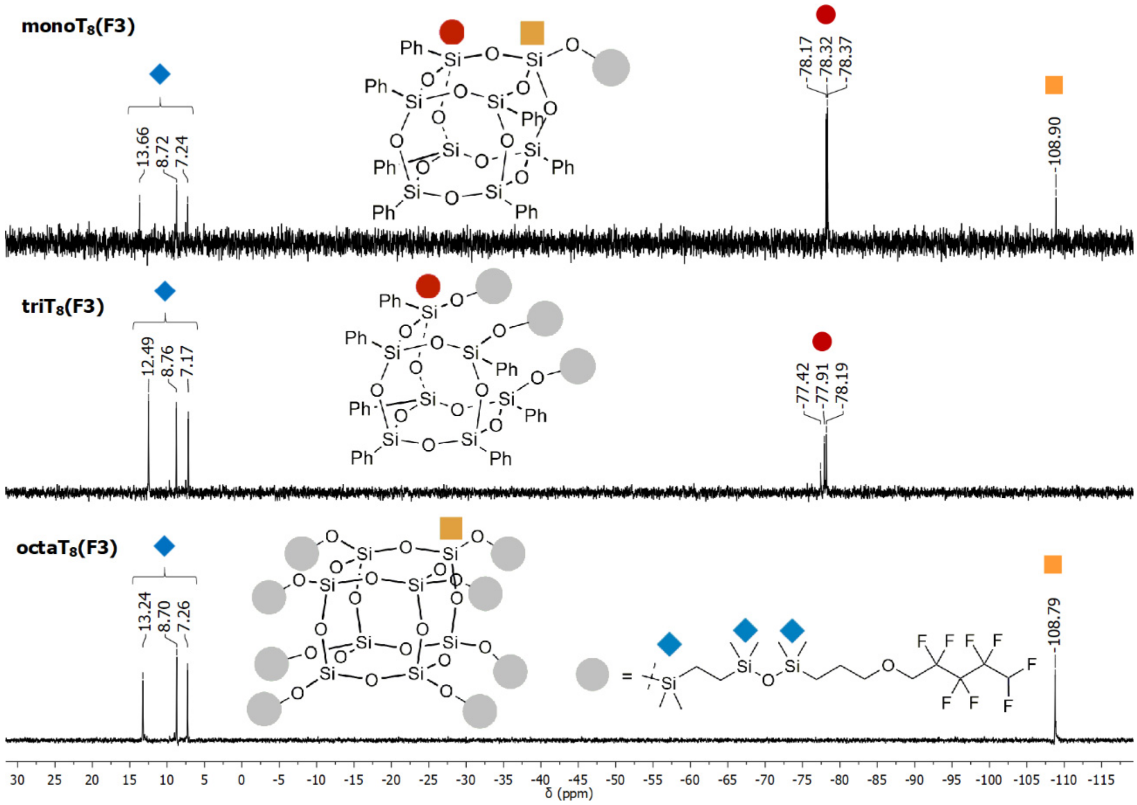

Figure S 44. Selected range of stacked ^{29}Si-NMR spectra of **monoT8(F3)**, **triT8(F3)** and **octaT8(F3)**.

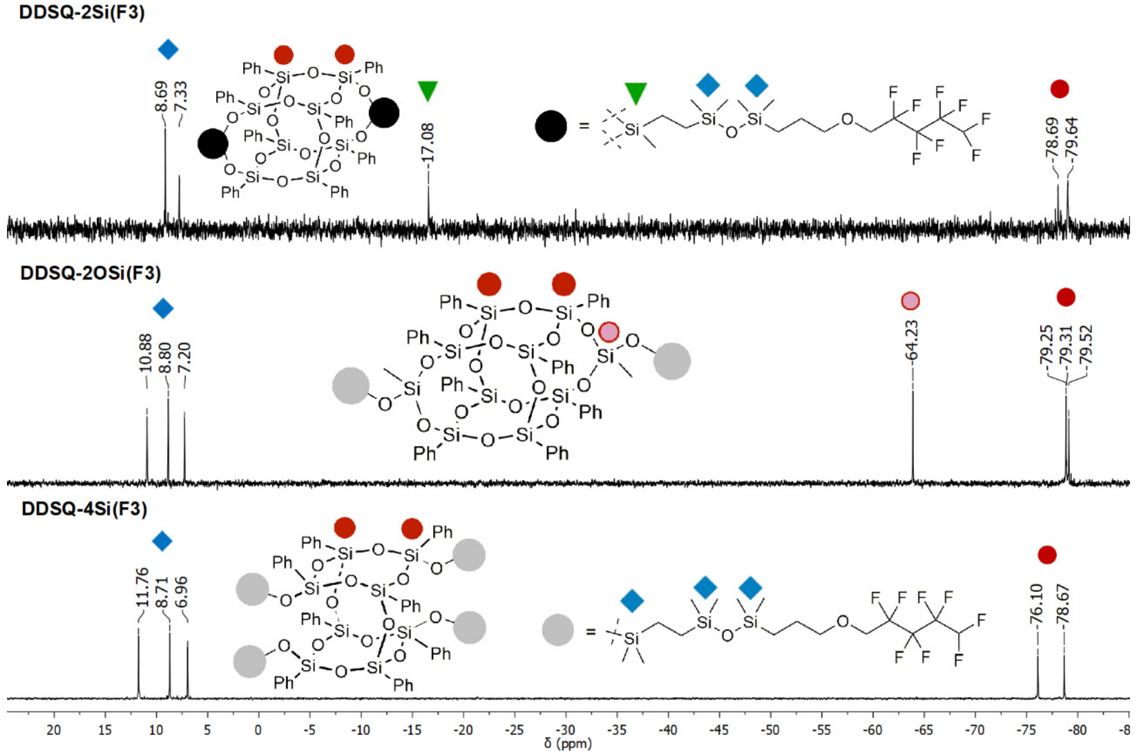

Figure S 45. Selected range of stacked ^{29}Si-NMR spectra of **DDSQ-2Si(F3)**, **DDSQ-2OSi(F3)** and **DDSQ-4Si(F3)**.

4. Dielectric Relaxation Spectra and Tables

Table S2. Values of dielectric permittivity, measured at -100°C at $10^0 \times 10^6$ Hz

Sample	ε at -100 °C						
	1 Hz	10 Hz	100 Hz	1 kHz	10 kHz	100kHz	1 MHz
**DDSQ-4OH**	20.01	19.94	19.96	19.93	19.95	19.96	20.10
**DDSQ-2SiVi**	19.86	19.91	19.93	19.92	19.94	19.97	20.11
**DDSQ-2SiH**	17.36	17.35	17.36	17.36	17.37	17.37	17.44
**DDSQ-2Si(F1)**	18.26	18.23	18.17	18.05	17.89	17.67	17.52
**DDSQ-2Si(F3)**	15.71	16.08	16.12	16.09	16.09	16.11	16.30
**DDSQ-2Si(F1)-f***	3.06	3.06	3.05	2.73	1.94	1.80	1.80
**DDSQ-2Si(F3)-f***	2.86	2.86	2.84	2.82	2.80	2.78	2.76

For dielectric measurements, the powder material was formed into the form of pellet. *Thin films in one layer prepared by a spin-coating technique using 5-10 wt% solutions of co-polymers in DCM (or DCM/THF (1:1)) deposited on the still plate.

Table S3. Values of dielectric permittivity, measured at 85°C at $10^0 \times 10^6$ Hz.

Sample	ε at 85 °C						
	1 Hz	10 Hz	100 Hz	1 kHz	10 kHz	100kHz	1 MHz
**DDSQ-4OH**	18.80	18.77	18.77	18.82	19.65	19.85	20.06
**DDSQ-2SiVi**	18.99	18.94	18.96	18.93	18.95	19.29	22.58
**DDSQ-2SiH**	16.58	16.56	16.82	16.94	17.29	16.82	17.44
**DDSQ-2Si(F1)**	29.91	25.11	22.74	22.06	21.99	22.74	22.58
**DDSQ-2Si(F3)**	21.40	19.82	19.57	19.48	19.79	20.05	20.25
**DDSQ-2Si(F1)-f***	3.03	3.03	2.66	1.95	1.84	1.83	1.83
**DDSQ-2Si(F3)-f***	5.24	4.96	4.63	4.62	4.62	4.98	4.92

For dielectric measurements, the powder material was formed into the form of pellet. *Thin films in one layer prepared by a spin-coating technique using 5-10 wt% solutions of co-polymers in DCM (or DCM/THF (1:1)) deposited on the still plate.

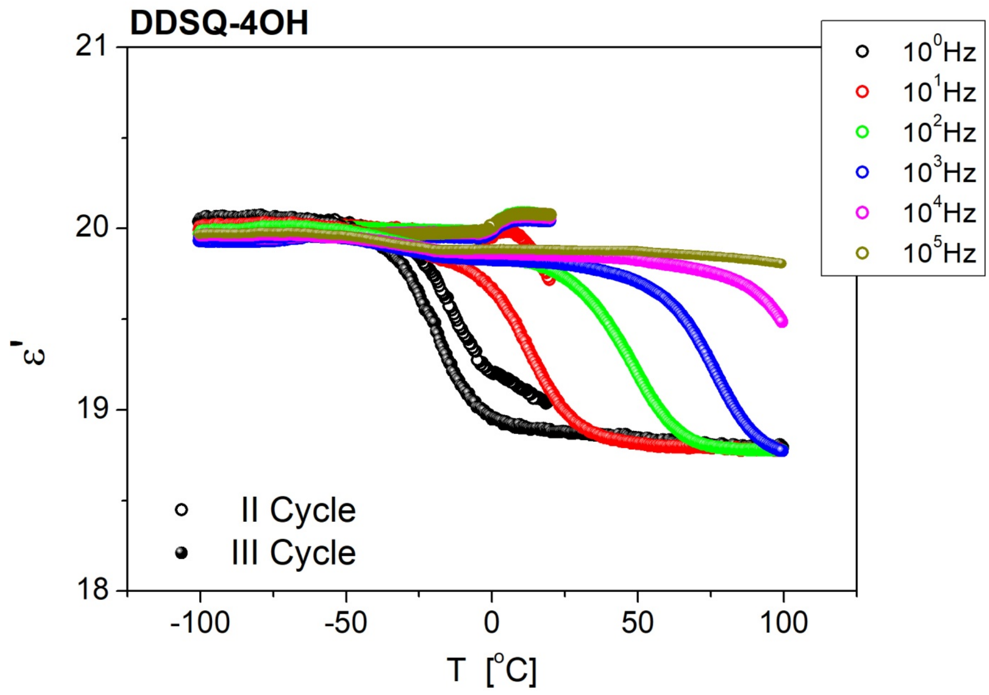

Figure S 46. Temperature dependence of dielectric permittivity, measured for **DDSQ-4OH** sample.

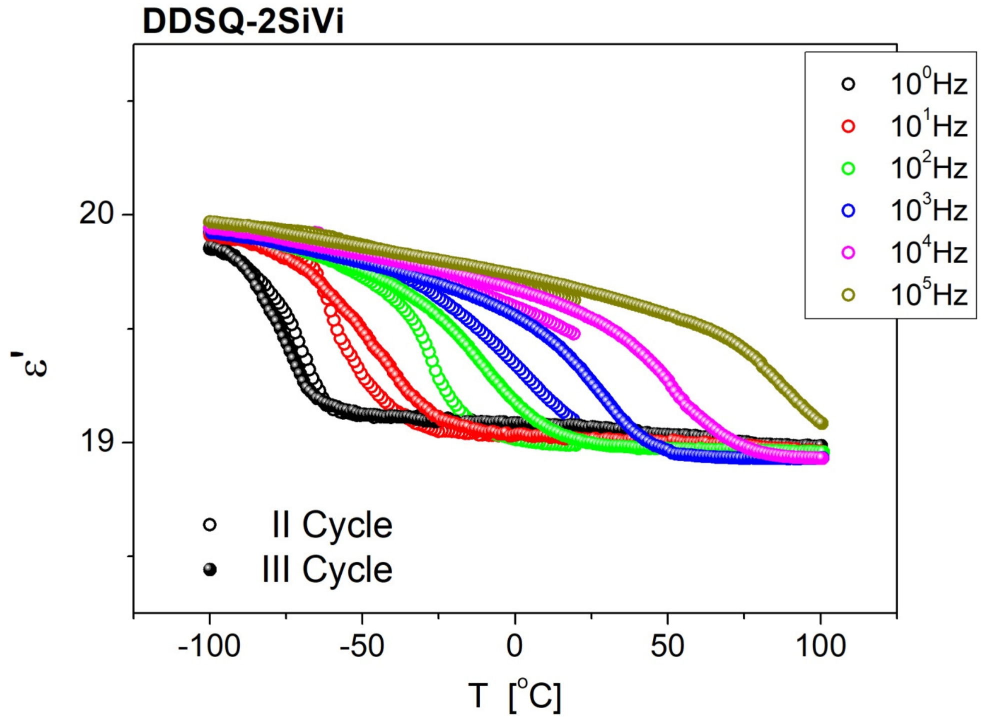

Figure S 47. Temperature dependence of dielectric permittivity, measured for **DDSQ-2SiVi** sample.

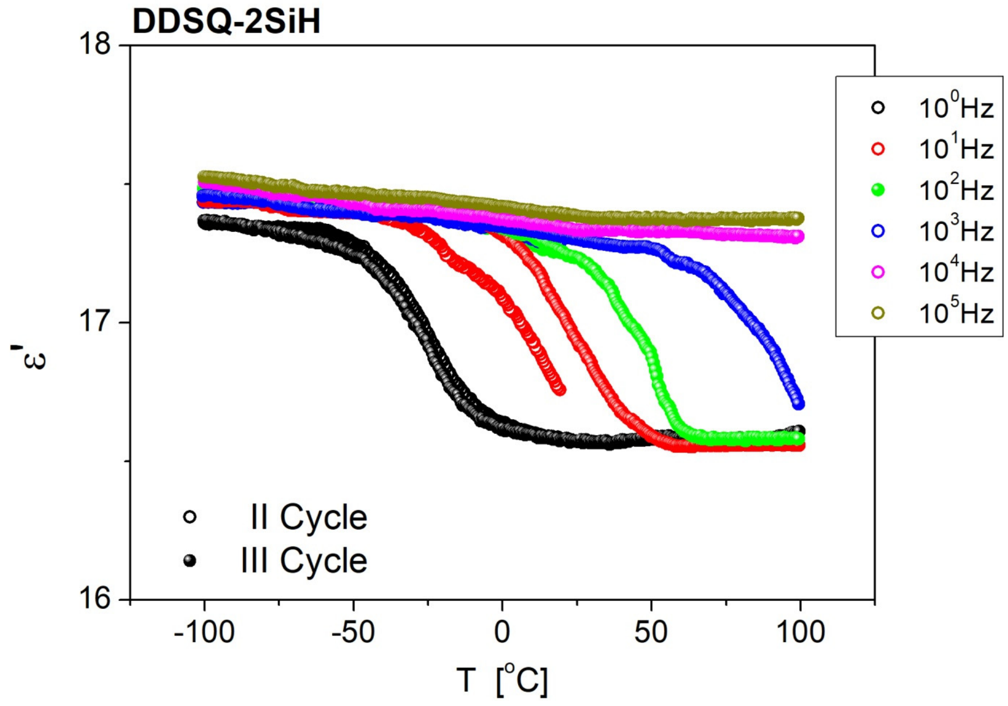

Figure S 48. Temperature dependence of dielectric permittivity, measured for **DDSQ-2SiH** sample.

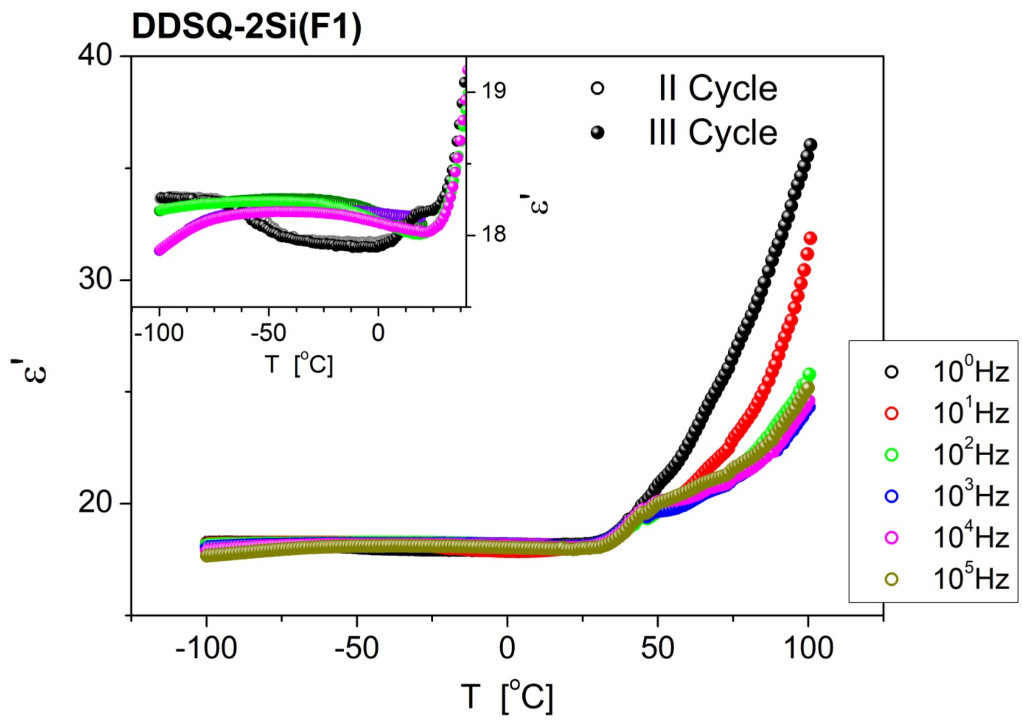

Figure S 49. Temperature dependence of dielectric permittivity, measured for **DDSQ-2Si(F1)** sample.

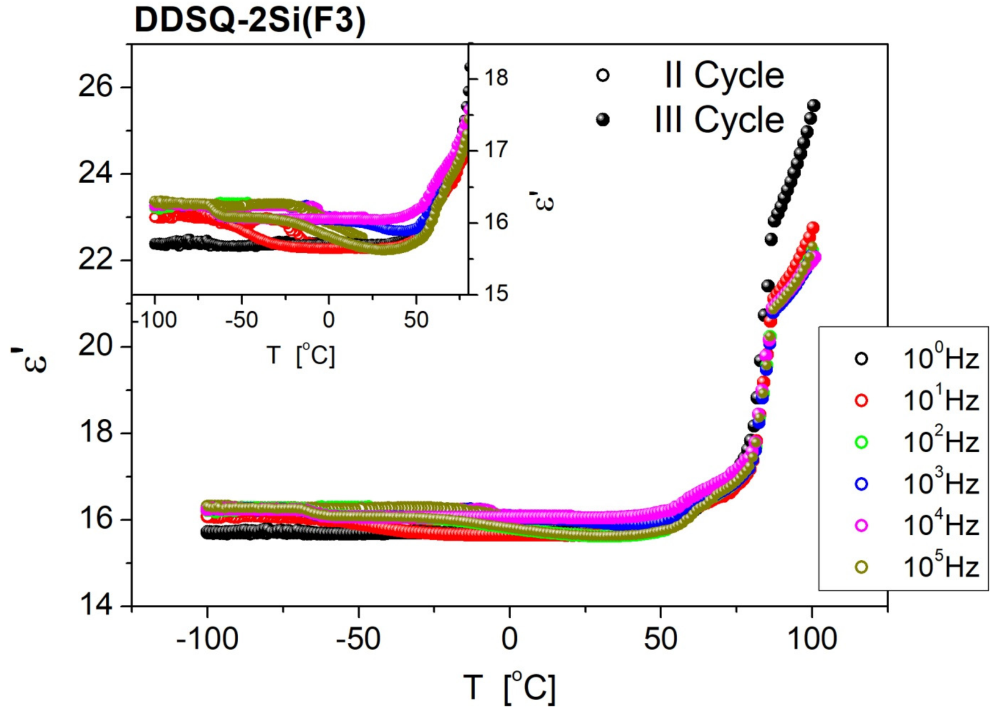

Figure S 50. Temperature dependence of dielectric permittivity, measured for **DDSQ-2Si(F3)** sample.
